# A visible-light-driven molecular motor based on barbituric acid†

**DOI:** 10.1039/d3sc03090c

**Published:** 2023-07-20

**Authors:** Kim Kuntze, Daisy R. S. Pooler, Mariangela Di Donato, Michiel F. Hilbers, Pieter van der Meulen, Wybren Jan Buma, Arri Priimagi, Ben L. Feringa, Stefano Crespi

**Affiliations:** a Stratingh Institute for Chemistry, University of Groningen Nijenborgh 4 9746 AG Groningen The Netherlands b.l.feringa@rug.nl stefano.crespi@kemi.uu.se; b Faculty of Engineering and Natural Sciences, Tampere University FI-33101 Tampere Finland; c European Laboratory for Non Linear Spectroscopy (LENS) via N. Carrara 1 50019 Sesto Fiorentino Italy; d ICCOM-CNR via Madonna del Piano 10 50019 Sesto Fiorentino FI Italy; e Van't Hoff Institute for Molecular Sciences, University of Amsterdam Science Park 904 1098 XH Amsterdam The Netherlands; f Institute for Molecules and Materials, FELIX Laboratory, Radboud University Toernooiveld 7c 6525 ED Nijmegen The Netherlands; g Department of Chemistry, Ångström Laboratory, Uppsala University Box 523 751 20 Uppsala Sweden

## Abstract

We present a class of visible-light-driven molecular motors based on barbituric acid. Due to a serendipitous reactivity we observed during their synthesis, these motors possess a tertiary stereogenic centre on the upper half, characterised by a hydroxy group. Using a combination of femto- and nanosecond transient absorption spectroscopy, molecular dynamics simulations and low-temperature ^1^H NMR experiments we found that these motors operate similarly to push–pull second-generation overcrowded alkene-based molecular motors. Interestingly, the hydroxy group at the stereocentre enables a hydrogen bond with the carbonyl groups of the barbituric acid lower half, which drives a sub-picosecond excited-state isomerisation, as observed spectroscopically. Computational simulations predict an excited state “lasso” mechanism where the intramolecular hydrogen bond pulls the molecule towards the formation of the metastable state, with a high predicted quantum yield of isomerisation (68%) in gas phase.

## Abstract 2

Yeehaw! The rotation of a visible-light-driven molecular motor based on barbituric acid is tamed by a hydrogen bonding “lasso” mechanism between its serendipitously-formed tertiary hydroxy stereocentre and its lower half carbonyl groups.

## Graphical abstract

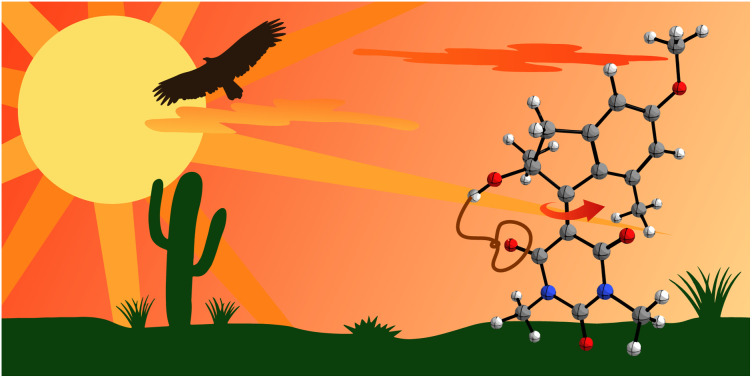

## Introduction

**Fig. 1:**
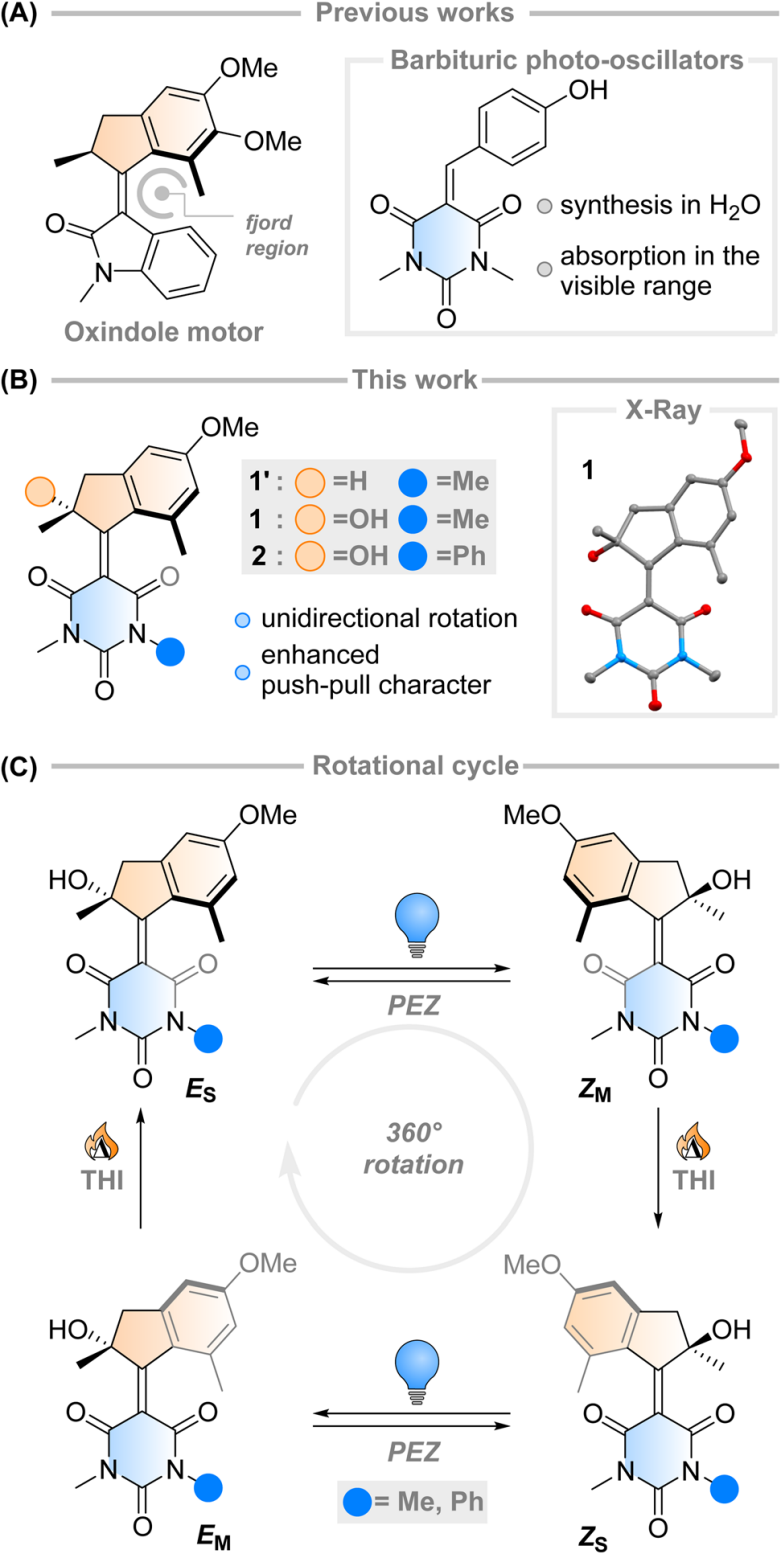
(A) Previous literature examples that led to the Feringa-type light-driven molecular motor presented in this work. The molecule is based on a charge-transfer oxindole structure and barbituric acid-based photo-oscillators. (B) This work, an investigation of barbituric acid-based molecular motors 1 and 2 (X-ray structure of 1: non-H atoms represented as 50% probability ellipsoids, H-bond evidenced for clarity). (C) Proposed rotational cycle of motors 1 and 2.

The fast (<200 fs)^1^ and efficient (quantum yield, *φ* = 67%)^2^ light-triggered nanoscale motion of the protonated Schiff base of 11-*cis*-retinal (PSBR) represents a continuous inspiration for chemists developing photoactive molecules based on *E*–*Z* isomerisation which can harvest light as effectively as the ones designed by Nature.^3–6^ Among these, light-driven rotary molecular motors have been developed (Fig. 1A), which absorb photons to fuel the unidirectional rotation of a rotator moiety about a stator moiety through successive photochemical *E*–*Z* isomerisations and thermal ratcheting steps.^7,8^ These motors can undergo repetitive, unidirectional rotary motion,^8–13^ can be integrated into larger artificial molecular machines,^14,15^ and are the key components to drive complex systems out of equilibrium.^16–18^ Their ability to control dynamic movement at the molecular level is a marvellous prospect, with potential applications ranging from biotechnology^19–21^ to materials science.^22–24^ Further adaptation of these molecular motors towards structures similar to PSBR stimulated the development of an oxindole-based molecular motor (Fig. 1A), exploiting an engineered electronic charge-transfer effect which drives the decay from the excited state in a similar manner to PSBR.^25^

Taking direct inspiration from the molecular structure of a natural product and translating it into a synthetic molecule is a powerful biomimetic strategy, which has been employed successfully by Olivucci and co-workers with respect to PSBR, giving rise to the positively charged *N*-alkylated indanylidene-pyrrolinium (NAIP) photoswitches that demonstrate similar photoreaction dynamics to PSBR.^26–29^ As another example, the biomimetic strategy has also been applied to the chromophore of the Green Fluorescent Protein (GFP), another natural product with impressive photoactivity,^30^ to synthesise *p*-hydroxydimethylindanylidene-oxopyrroline (*p*-HDIOP) anionic photoswitches.^31^ In both NAIP and *p*-HDIOP photoswitches, the *E*–*Z* isomerisation is driven by biomimetic charge-transfer character in S_1_, a result echoed in the aforementioned oxindole-based molecular motor (Fig. 1A).^25^

Barbituric acid is a readily available chemical building block with a strongly electron-withdrawing core. For this reason, it has remarkably acidic methylene protons (p*K*_a_ ≈ 4) enabling Knoevenagel condensations with various electrophiles under mild conditions.^32^ Its electron-withdrawing nature has also been exploited in the design of dyes and photoswitches with push–pull character, most notably donor–acceptor Stenhouse adducts.^33^ Recently, a family of photo-oscillators based on barbituric acid were introduced, featuring high molar absorptivity between 350–400 nm and ultrafast photodynamics (Fig. 1A).^34^ They can be readily obtained in high yields by an easily accessible synthetic route *via* a Knoevenagel condensation in water. However, the possibility of utilising barbituric acid derivatives in photoswitches or molecular motors has not been explored yet.

Intrigued by this work, we envisioned that an overcrowded alkene-based design featuring barbituric acid in the lower half could operate as a second-generation Feringa-type molecular motor with high visible light absorptivity (1′, Fig. 1B). Surprisingly, when attempting to synthesise motor 1′ we consistently obtained motor 1, with a stereocentre featuring a tertiary alcohol. Mechanistic experiments support the presence of a peroxide as an intermediate in the formation of the motor and quantum chemical calculations show that 1 is 2.4 kcal mol^−1^ more stable than its hydrogenated counterpart 1′ (see ESI† for further details). These results hint towards the formation of a thermodynamically favoured product in the dynamic covalent chemistry of barbituric acid condensation.^32^

Inspired by this serendipitous discovery, we report the detailed investigation of the photochemical *E*–*Z* (PEZ) steps that convert the stable states (*E*_S_ or *Z*_S_) into the respective isomerised metastable states (*Z*_S_ or *E*_M_), and thermal helix inversion (THI) steps of the novel motor 1 (Fig. 1C).^25^ We show that the OH group present in 1 plays a crucial role in the S_1_ photoisomerisation step: as the lower half rotates, the OH group forms hydrogen bonds with the carbonyl groups on the barbituric acid half, actively pulling the rotation forwards. The absence of a strong solvent effect and computational insights suggest that 1 may act as a motor in apolar solvents, with its unidirectional efficiency decreasing in more polar ones.^35^

## Results and discussion

### Synthesis

**Scheme 1:**
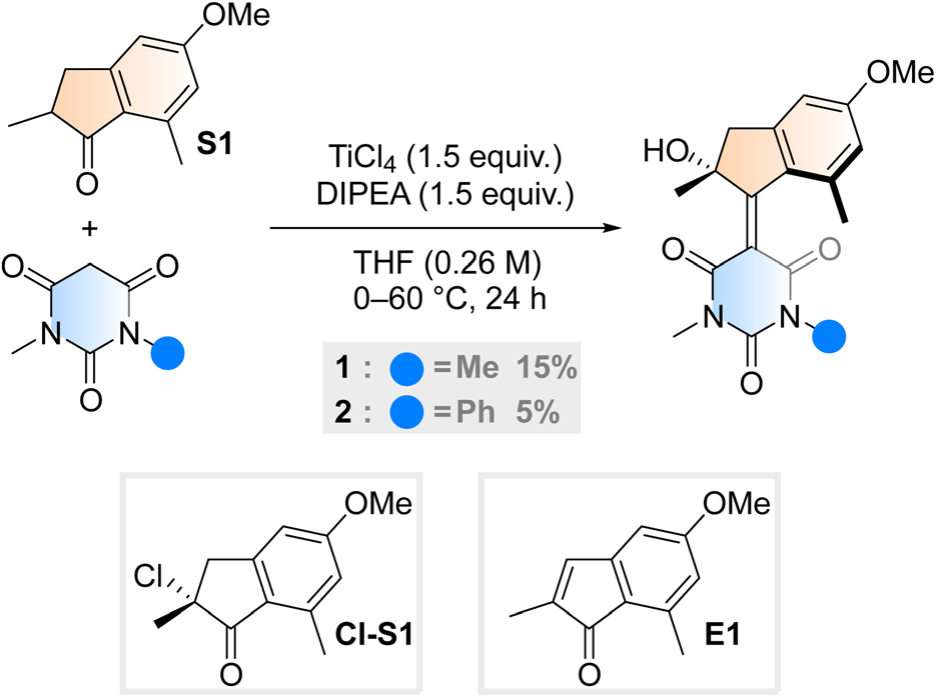
Synthetic procedure for motor 1 and 2, initially hypothesised alpha-chlorinated intermediate Cl-S1 and elimination side product, E1.

Aiming to further expand the scope of motors operating continuously at room temperature,^36–38^ we reacted the barbituric acid moiety with an upper half featuring a five-membered ring because the combination of the latter with six-membered lower halves typically provides motors with low THI barriers.^4^ The ketone precursor is synthesised in one step from 3-methylanisole and methacrylic acid in a Friedel–Crafts acylation followed by a Nazarov cyclisation, facilitated by polyphosphoric acid (PPA). To attain our motor design, we tested typical Knoevenagel conditions reported for barbituric acid condensations with ketones.^39^ Contrary to previously published reactions involving less hindered ketones or aldehydes,^34^ we only observed reactivity when both a Lewis acid (TiCl_4_) and a non-nucleophilic base (diisopropylethylamine, DIPEA) were used^40^ (Scheme 1). Even though barbituric acid is more enolisable than oxindole,^25,40^ the reaction was markedly slower than in our previous studies on oxindole-based motors: after 24 hours at 60 °C, 65% of the ketone precursor was recovered. Most surprisingly, instead of the typical tertiary stereogenic centre at the allylic position of overcrowded alkene-based motors (1′, Fig. 1B), we isolated exclusively compound 1, featuring a stereocentre with a hydroxy group at the tertiary carbon (Scheme 1). To the best of our knowledge, this alpha-hydroxylation during a Knoevenagel condensation is unprecedented in the literature. In addition to 1 and recovered starting materials, the reaction yielded varying amounts of an elimination product E1 (see Scheme 1) as well as side products originating from the reaction between TiCl_4_ and tetrahydrofuran.^41^

To gain insight into the mechanism of the unexpected reaction, we explored how various parameters affected its outcome. The product composition was unaffected by ambient light, residual moisture, atmospheric oxygen and whether the reaction was quenched with water, deuterium oxide or methanol (see ESI, Table S1†). Our initial hypothesis of an alpha-chlorinated intermediate was refuted when no product was acquired starting from the alpha-chloroketone Cl-S1 (Scheme 1). We also ran the reaction with an ^18^O-labelled S1, yielding 1 in an identical yield to a control reaction run in parallel, and with no trace of ^18^O in the structure (see ESI, Fig. S1†). Thus, we concluded that the unexpected oxygen originates from outside the starting materials and not from a rearrangement reaction. Probing the reaction mixture directly to record any reactive intermediates was attempted by running the reaction in THF-d_8_, but NMR on the unquenched reaction mixture could not be measured possibly due to paramagnetic species forming during the reaction.

However, some hints to the mechanism were obtained by studying the reaction mixture with UPLC-MS immediately after quenching. Interestingly, instead of the expected [M + H]^+^ and [M + Na]^+^ signals for 1, we observed for each *m*/*z* values 16 mass units larger. After full work-up, the expected signals for 1 were observed. This result might suggest that the reaction proceeds through the addition of an O_2_ molecule forming a peroxide intermediate, and that the O–O bond is broken during the work-up. However, such a mechanism seems to be at odds with the observation that the reaction appears to proceed similarly under ambient conditions and in carefully degassed solutions under an argon atmosphere.

Single crystals of 1 suitable for X-ray diffraction were grown from a saturated solution of the compound in an EtOAc/pentane mixture (see Fig. 1 and ESI†). Compound 1 crystallised with two molecules of 1 in the unit cell, both possessing the same chirality at the methyl stereocentre. The molecules have a C=C bond length of 1.38 Å, a value which is relatively elongated compared to typical overcrowded alkenes (∼1.35 Å).^40,42^ This result may allude to a higher degree of single-bond character in the central alkene bond, possibly due to push–pull nature arising from the highly electron-withdrawing barbituric acid lower half and the electron-donating OMe group on the upper half. The dihedral angles (angle C_A_C_B_C_C_C_D_, see Scheme 1 for labelling) of both motors in the unit cell were 27.35° and 29.70° respectively, consequently showing helical chirality, a key requisite for unidirectional molecular motors.^4^ Additionally, due to the hydroxy group at the stereocentre, compound 1 undergoes hydrogen bonding in the solid state. The preferred intramolecular interaction is reflected in the length of the H-bond between the OH and the C=O group on the barbituric acid moiety, which is either 2.61 or 2.65 Å, while the intermolecular H-bonding between the OH group and the OMe group on a neighbouring molecule is considerably longer (3.03 Å, see Fig. 1 and S7†).

Desymmetrised motor 2 was synthesised using the same method as motor 1 but with 1-methyl-3-phenylbarbituric acid as the lower half reactant. The asymmetric barbituric acid lower half was prepared in two steps from methylamine, phenyl isocyanate and malonic acid. The Knoevenagel step was slower and lower-yielding than for 1, but the same stereocentre bearing an OH group was formed. The product was isolated as a 1 : 1 mixture of the *E* and *Z* isomers. They were separated by supercritical fluid chromatography (SFC, see ESI†) and subsequently concentrated by freeze-drying in the dark, to mitigate unwanted thermal and photochemical isomerisation processes. After purification, the 1 : 1 mixture could be enriched to contain a 73 : 27 ratio of both stable isomers (2a : 2c). Unfortunately, the exact stereochemistry of these isomers could not be determined, due to fast thermal and photochemical isomerisation at ambient conditions.

### Photochemical isomerisation

**Fig. 2:**
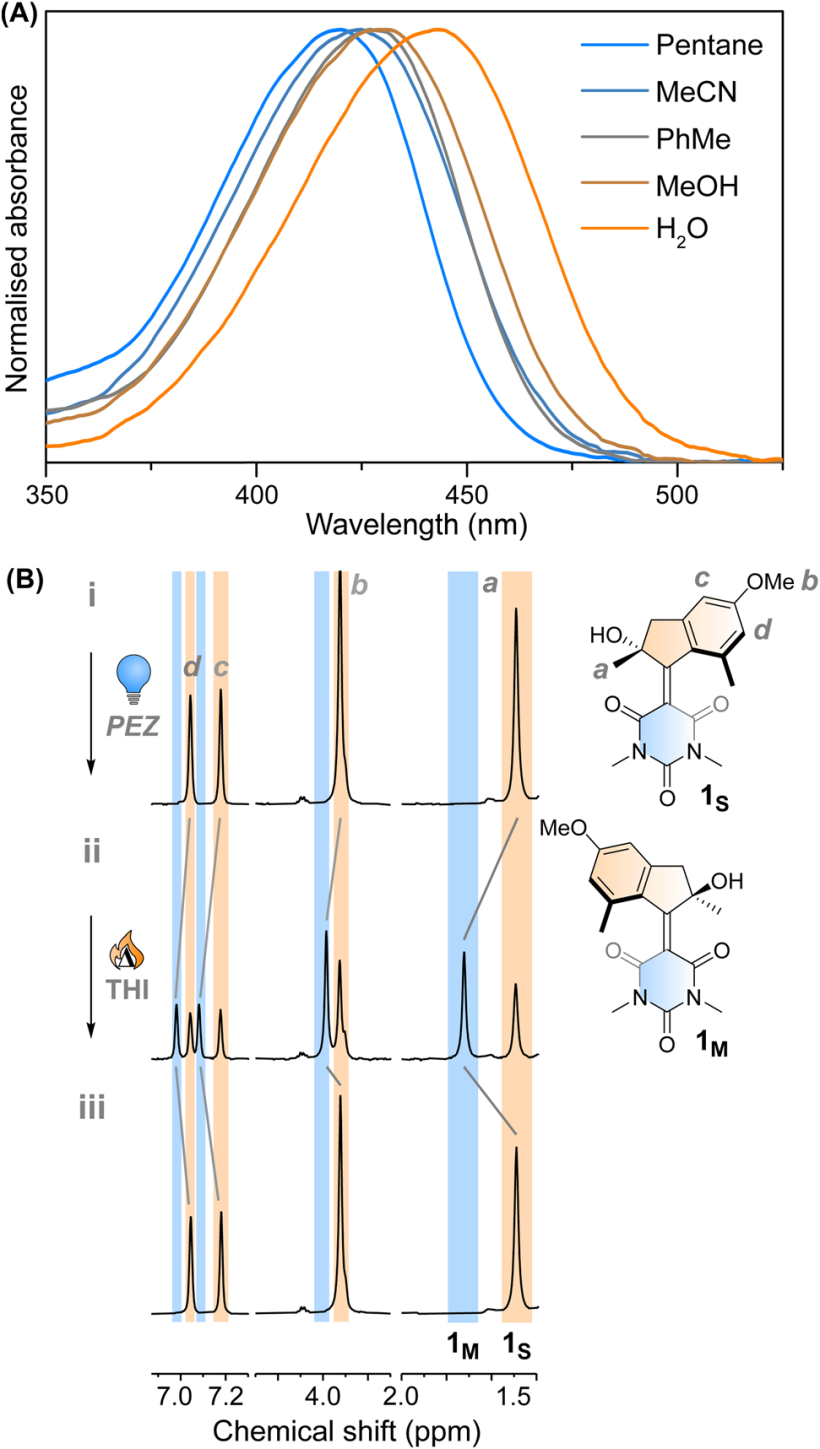
(A) Normalised UV-vis absorption spectra of 1_S_ in different solvents. (B) ^1^H NMR irradiation studies of 1_S_ in CD_3_OD (*c* = 1 × 10^−3^ M) at −90 °C. (i) 1_S_ before irradiation; (ii) PSS_420_, 63 : 37 (1_M_ : 1_S_); (iii) THI, −45 °C, 15 min.

Motor 1 shows an absorption maximum between approximately 420–445 nm in multiple solvents (Fig. 2A and Table 1) with a solvatochromic effect, reflecting the push–pull nature of the molecule. The molar absorption coefficients reflect the ππ* nature of the first absorption band. Since 1 has an asymmetric centre alpha to the central alkene axle, the structure presents strong similarities with typical molecular motors.^43^ Due to the symmetrical nature of the lower half, we predict that 1 will act similarly to a second-generation motor with only two isomers: a stable isomer, 1_S_ and a metastable isomer, 1_M_. By extension, the asymmetric structure 2 will operate in a 4-step process, as described by Fig. 1C.

**Table 1:** Photophysical parameters of motor 1 in different solvents, excited state lifetimes of 1_s_ and thermal lifetimes of 1_m_ as measured by laser flash photolysis. The solvents are ordered according to increasing polarity

	*λ*_max_ (nm)	*ε* (M^−1^ cm^−1^)	*τ*_1_1_s_* (ps)	*τ*_2_1_s_* (ps)	*τ*_3_1_s_* (ps)	*τ*1_m_ → 1_s_ (10^−2^ s)
Pentane	425	19 200				1.23
Hexane			0.5	10.5		
1,4-Dioxane	421	18 900				1.97
Toluene	441	18 500	0.6	8.5	38.2	2.88
DCM	432	20 400	0.7	6.6	30.3	7.47
THF	428	18 300				1.17
Decanol	433	18 100				1.46
*i*PrOH	435	19 100				0.588
EtOH	428	20 100				0.181
MeOH	432	19 100	0.7	3.3	44.4	0.134
MeCN	424	18 600	0.6	1.5	13.7	3.42
Glycerol	430	15 300				0.181
DMSO	432	14 400	0.7	6.8	170.9	7.55
H_2_O	444	12 800				7.87 × 10^−4^

We studied the ultrafast excited state behaviour of compounds 1 and 2 by measuring their transient absorption spectra upon excitation at 400 nm in multiple solvents. The ultrafast behaviour of the two compounds is similar, so we will describe the behaviour of compound 1 and refer the reader to the ESI† for compound 2.

**Fig. 3:**
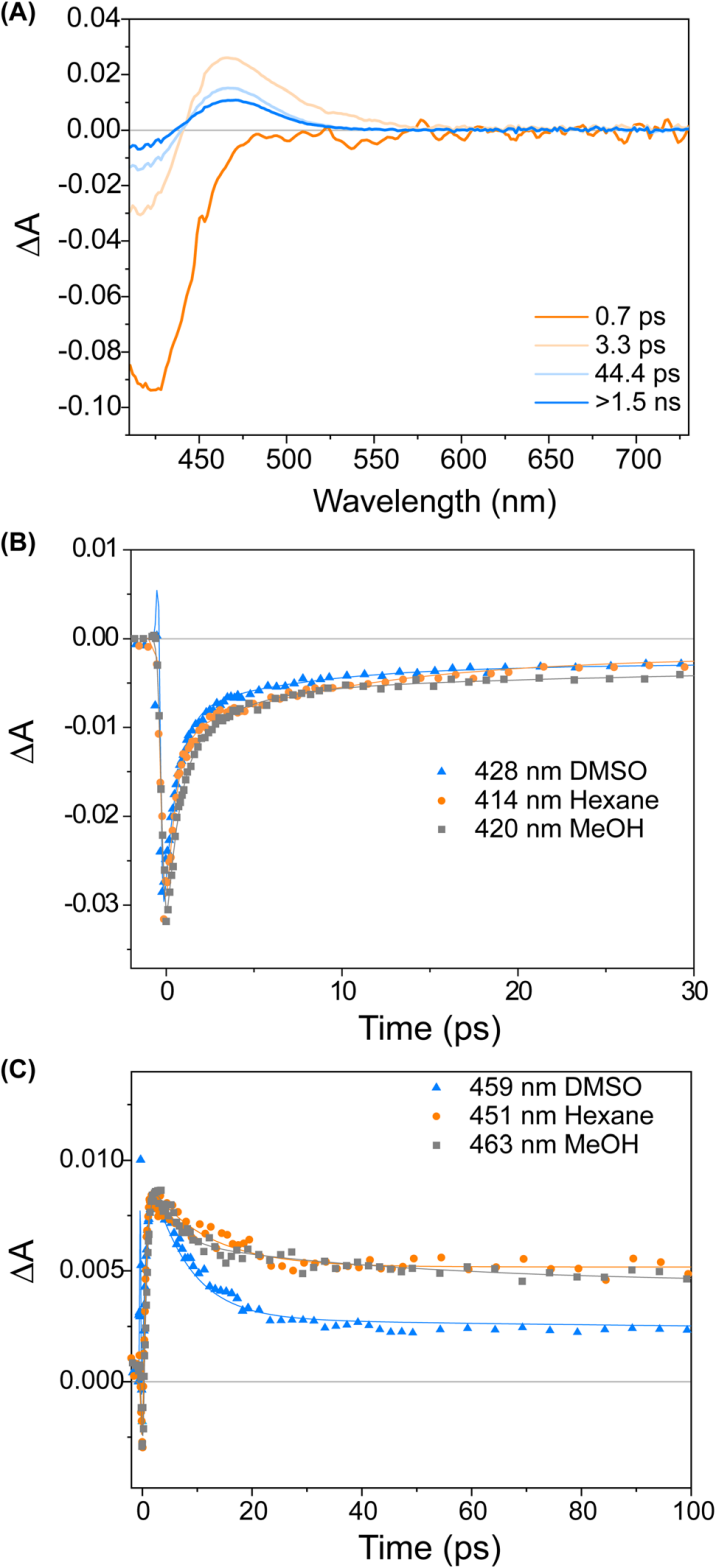
(A) Evolution Associated Difference Spectra (EADS) obtained from global analysis of the transient absorption data of compound 1 recorded in MeOH. (B) Comparison of kinetic traces recorded on the ground state bleach band of compound 1 in various solvents. (C) Comparison of kinetic traces recorded on the product absorption band of compound 1 in various solvents. The solid lines represent the global fit of the signals.

Fig. 3 displays a selection of transient absorption spectra recorded upon 400 nm excitation of solution of 1 in MeOH. As noticed by looking at the time/wavelength map (Fig. 3A), an intense negative band peaking at ∼430 nm appears immediately after excitation. Considering the steady-state absorption of the samples, this band is attributed to ground state bleaching. A very broad and less intense negative signal is also noticed, extending up to 650 nm, associated with a very weak stimulated emission (see ESI†). This transient signal is a fingerprint of the emissive Franck–Condon state in molecular motors.^25,44^ In less than 1 ps, the ground state bleaching signal recovers significantly and a red-shifted positive band develops, which we assign to the absorption of the photoisomerisation product. This band indicates that once excited, the sample rapidly reaches a conical intersection (CInt)^45^ and subsequently returns to the ground state, S_0_. The excited sub-ps dynamics is similar to the one observed in the barbituric acid photo-oscillators.^34^ During the ensuing evolution occurring on the picosecond time scale, the intensity of the differential signal decreases without a significant change of the bandshape.

We attribute these dynamics to vibrational and solvent-induced relaxation of the photoproduct in the ground state. We assign the final signal to the metastable state (1_M_), which is characterised by a red-shifted absorption band for molecular motors.^25,44^

This assignment is also confirmed by ^1^H NMR at −90 °C in CD_3_OD. A new set of signals were formed upon *in situ* irradiation with 420 nm light, which we ascribe to 1_M_, giving a photostationary distribution (PSD) of 63 : 37 1_M_ : 1_S_ (Fig. 2B). The isomerisation can be clearly followed by the substantial shift of the stereogenic group protons, H_a_, from 1.51 ppm to 1.62 ppm. Under these conditions, no photodegradation was observed, proving motor 1 to be photochemically robust. Furthermore, we analysed the solvent dependence of the actinic process by measuring the transient absorption spectra of the compounds in a variety of solvents of different polarity/polarisability. As noticed by the comparison of the kinetic traces recorded at the maximum of the bleaching signal (Fig. 3B), the dynamics of the excited state evolution is almost independent from the solvent properties. Comparing the kinetic traces recorded on the product band, we instead notice a higher solvent dependence (Fig. 3C).

This effect is attributed to the different solvent-induced relaxation dynamics of the 1_M_ that reaches the ground state on a sub-ps scale, in a vibrationally hot state. It appears that the relaxation process is slightly faster in polar solvents, such as DMSO, than in non-polar ones, like hexane, but this may also be attributed to viscosity^46^ and the possibility to interact with the hydroxy group of the upper half. The relaxation dynamics of compound 2 is slightly more sensitive to the solvent nature as compared to compound 1 (see ESI, Fig. S9†).

We performed an excited state molecular dynamics simulation of 1_S_ at the OM2/MRCI level of theory^47–49^ to obtain a more qualitative understanding of the excited state dynamics. Out of the initial 400 initial conditions obtained from a Wigner sampling, 332 were successfully propagated for 2 ps using an NVT ensemble with a Nosé-Hoover thermostat after initial excitation to the S_1_ state. The simulation predicted an excited-state lifetime of 0.53 ps in the gas phase, which is in excellent agreement with the experimental value of 0.5 ps obtained from the femtosecond spectroscopy experiments in hexane (see Table 1 and ESI† for details on data analysis). In our simulation, the ensemble proceeds following a rotational movement of the double bond towards a perpendicular configuration of the upper and lower halves. In this region, characterised by a dihedral angle of rotation about the double bond of 90°, the molecule funnels to the ground state *via* a CInt, populating either the metastable 1_M_ or leading unproductively back to 1_S_.

**Fig. 4:**
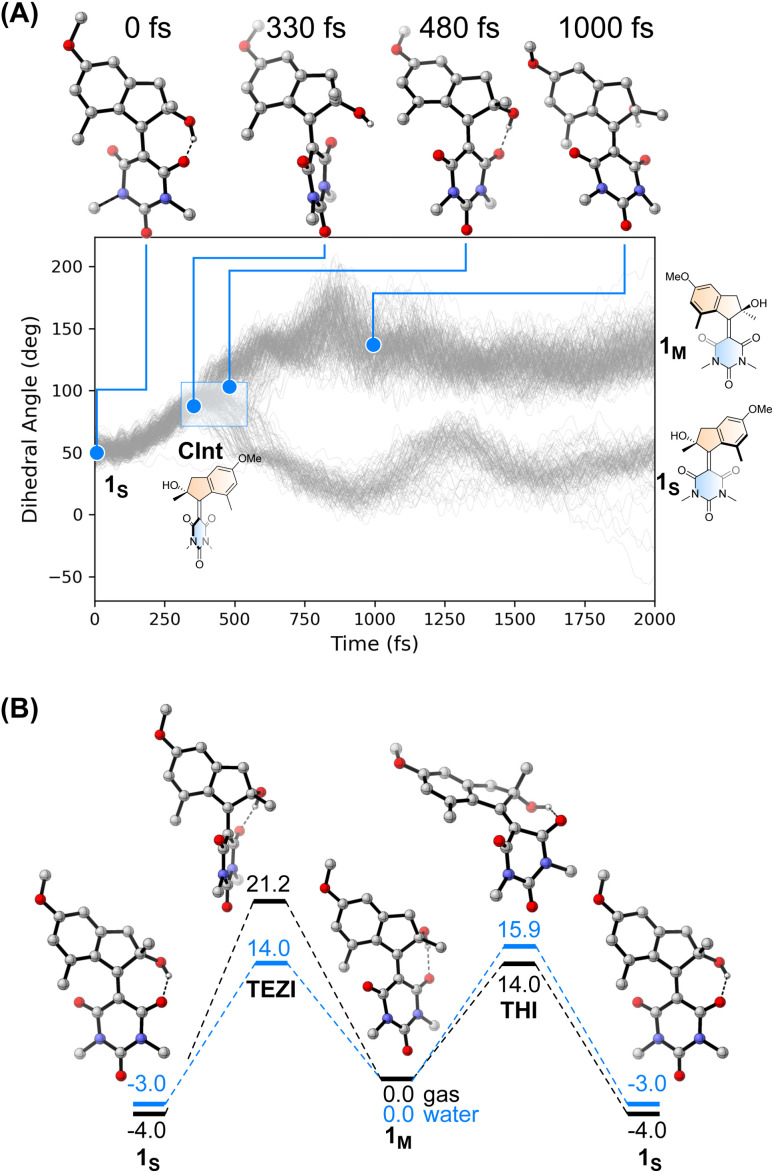
(A) Evolution of the dihedral angle associated with the rotation about the central C=C bond over time obtained at the OM2/MRCI level. It is possible to appreciate the different distribution of 1_M_ and 1_S_ after reaching the Cint region (highlighted in the figure). Top: Snapshots of the isomerisation of 1 selected from a productive MD simulation that led to the formation of 1_M_ underlining the hydrogen bond “lasso” effect. (B) Gibbs free energies (DSD-BLYP-D3BJ/def2-QZVP//r^2^SCAN-3c level, in kcal mol^−1^) for the thermal processes of motor 1 on the ground state surface. The black surface depicts the energies of the structures computed in the gas phase. The blue surface, in contrast, represents the energies of the structures computed using water as implicit solvent (CPCM).

Interestingly, we predicted the excited state of the molecule to be diradical in nature along the entire isomerisation path, explaining the limited effect that the solvent has on the actinic process. The simulation conducted in the gas phase led to a predicted quantum yield of 68% (see Fig. 4A), a high value in which the hydroxy group and the absence of explicit solvents interacting with it could play a crucial role. Indeed, after excitation the hydrogen bond between the OH group and the C=O in its immediate proximity is lost, with the concomitant rotation of the lower half about the double bond. Immediately after the molecule reaches the perpendicular orientation of the Cint and funnels through it to the ground state, the OH starts to interact with the second C=O, leading to a “lasso” effect that pulls the geometry towards the formation of the metastable state (see Fig. 4B). This behaviour is similar to the one predicted by García-Iriepa and coworkers, who designed computationally a retinal-based motor with high rotational speed and absence of thermal steps, thanks to the formation of hydrogen bonds imparting directionality to the motion.^50^ We also underline that the diradical nature of the excited state could be crucial in the sub-ps dynamics observed.^25^ Indeed, the limited pyramidalization at the computed conical intersection geometry could allow a fast rotational movement at the excited state, as hypothesized by Olivucci and Filatov.^5^ It is worth mentioning that the high quantum yield values could be a reflection of the absence of medium in the molecular dynamics simulation. Indeed there are different reports that confirm the fundamental contribution of explicitly treated solvent molecules in lowering the predicted efficiency of the photochemical step.^36^ This observation is further explored in a recent preprint of Olivucci *et al.* focusing on the explicit cavity effect on the computed quantum yields of Rhodopsin and the biomimetic molecular rotor *para*-methoxy *N*-methyl indanylidene-pyrrolinium.^51^

### Thermal isomerisation steps

The thermal pathway of isomerisation of 1 was studied using nanosecond transient spectroscopy. After the pump pulse, we were able to observe the formation of a transient signal red-shifted compared to the ground state bleach (see ESI†), which we assigned to the absorption of the metastable state 1_M_ in accordance with the femtosecond transient spectroscopy data (*vide supra*). In most of the solvent analysed, we noted only a minimal effect of the solvent polarity on the recovery of the metastable state (see Table 1). This finding is expected for the THI process,^52^ and is supported by our calculations at the DSD-BLYP-D3BJ/def2-QZVP//r^2^SCAN-3c level (see Fig. 4D), which predict the THI step to be considerably lower in energy than the transition step associated with the thermal *E*–*Z* isomerisation (TEZI). We found two pathways for the THI with barriers of 19 and 14 kcal mol^−1^ when considering the presence (or absence, respectively) of hydrogen bonding between the OH and the neighbouring C=O group of the lower half.

These values equate to lifetimes of milliseconds at room temperature, well in line with our experimental results (Table 1). However, when attempting to measure exchange spectroscopy (EXSY) in a D_2_O : DMSO-d_*6*_ (9 : 1) mixture, we observed gradual bleaching of the solution and the disappearance of the signals for 1. Hence, it is not possible to clearly assign the process observed in water, due to a competing decomposition pathway (see Fig. S5†).

The different solvent polarisability could have a more peculiar effect. We computed the barriers for TEZI and THI using both gas and water as implicit solvent at the CPCM level.^53^ We observed that the barrier for TEZI is higher in gas phase than THI (21 kcal mol^−1^*vs.* 14 kcal mol^−1^), but this is inverted in a highly polar medium, *viz.* water (14 kcal mol^−1^*vs.* 16 kcal mol^−1^).

This finding is supported by the OM2/MRCI computations which predict a zwitterionic, closed-shell nature for the TEZI transition state, explaining the stabilisation of the thermal C=C bond breaking pathway. Nevertheless, we predict this molecule to be a motor at room temperature in all solvents of different polarisability (although with different degrees of efficiency). Indeed, due to the relatively similar energy barriers between TEZI and THI, in water the thermal *E*–*Z* isomerisation remains a competing event which can be compared to the metastable to stable photochemical back isomerisation competing with the formation of 1_M_. Indeed, upon photochemical population of the metastable state, 1_M_ can revert back to 1_S_*via* TEZI which is slightly more favoured than the THI. However, considering the low values of both barriers (see Fig. 4B, blue lines), also the THI transition state will be accessible. Hence, the ratcheting step of the THI at room temperature will lead to an overall continuous unidirectional motion. Moreover, the similar values of the predicted thermal barriers explain the minimal difference observed with nanosecond transient spectroscopy when evaluating the metastable to stable thermal recovery. In addition, the presence of a solvent that weakens the intramolecular hydrogen bond lowers the barriers (Fig. S25†), but provides qualitative trends similar to the ones predicted in Fig. 4B.

To prove these hypotheses experimentally, photochemical switching of compound 2 was carried out in ^1^H NMR at −90 °C in CD_3_OD, in an effort to see sequential population of all four isomers in the rotation cycle (see ESI†). Upon irradiation of the enriched mixture of the diastereomeric stable isomers (77 : 23, 2a : 2c after SFC separation, dubbed as such due to the nontrivial assignment of the *E*/*Z* configuration to a specific set of NMR signals) with a 420 nm LED, two new sets of signals appeared, which we assign to the metastable isomers 2b and 2d. The PSS ratio at 420 nm was found to be 12 : 19 : 9 : 61 (2a : 2b : 2c : 2d) (Fig. S3†). The THI process was monitored at −90 °C in the NMR, and both metastable states fully converted back to their corresponding stable states after 70 min in the dark (Fig. S4†) to a new population of 73 : 27, 2a : 2c. From this data, it can be determined that both processes are unimolecular, that 2d converts thermally into 2a, and that 2b converts thermally into 2c. While the directionality of rotation cannot be univocally proven with this data, the variation of the distribution of the stable diastereomers hints towards a motor function.

## Conclusions

In summary, we present a novel light-driven rotary molecular motor with a lower half based on barbituric acid. The motor could be obtained in modest yields in a simple synthetic route, the key step being a Knoevenagel condensation. Surprisingly, a hydroxy unit is installed in the stereogenic centre during the condensation. Many modifications of the original synthetic strategy were ventured, however we cannot yet be sure of the mechanistic steps towards motor 1. Due to this unexpected reaction, the stereogenic centre on the upper half becomes a tertiary alcohol. We investigated the photochemical and thermal isomerisation steps with femtosecond- and nanosecond-scale transient absorption spectroscopy and low-temperature NMR spectroscopy. Motor 1 functions as a second-generation Feringa-type molecular motor, exhibiting sub-picosecond *E*–*Z* isomerisation dynamics under visible-light excitation and ultrafast thermal barriers for helix inversion. Compared to typical second-generation motors, 1 has high isomerisation quantum yields (68% estimated computationally), which is attributed to two factors: (i) the strongly electron-withdrawing nature of the barbituric acid lower half leads to efficient axial rotation, and (ii) hydrogen bonding between the fortuitous hydroxy group and the carbonyl groups of the lower half actively promotes the isomerisation.

## Data availability

The datasets supporting this article have been uploaded as part of the ESI.† All cartesian coordinates for all the compounds considered are provided as a separate additional file in a figshare repository with the following DOI: https://figshare.com/articles/dataset/dx_doi_org_10_6084_m9_figshare_6025748/6025748.

## Author contributions

K. K. and D. R. S. P. contributed equally to this work. D. R. S. P. and S. C. conceived the project and designed the molecules. K. K. synthesised all molecules and carried out all synthetic troubleshooting. D. R. S. P. and K. K. carried out NMR experiments and steady state UV-vis experiments. D. R. S. P. measured X-ray diffraction and carried out preparative SFC. S. C. performed computational calculations. M. D. D. and D. R. S. P. carried out femtosecond TA experiments. M. H., S. C. and W. J. B. performed nanosecond TA experiments. K. K., D. R. S. P. and S. C. wrote the manuscript. B. L. F., S. C., M. D. D., A. P. and W. J. B. supervised the work. All authors discussed and commented on the manuscript. S. C., A. P., and B. L. F. acquired funding.

## Conflicts of interest

There are no conflicts to declare.

## Supplementary Material

SC-014-D3SC03090C-s001

SC-014-D3SC03090C-s002

SC-014-D3SC03090C-s003
