# Correction: A visible-light-driven molecular motor based on barbituric acid

**DOI:** 10.1039/d3sc90158k

**Published:** 2023-08-21

**Authors:** Kim Kuntze, Daisy R. S. Pooler, Mariangela Di Donato, Michiel F. Hilbers, Pieter van der Meulen, Wybren Jan Buma, Arri Priimagi, Ben L. Feringa, Stefano Crespi

**Affiliations:** a Stratingh Institute for Chemistry, University of Groningen Nijenborgh 4 9746 AG Groningen The Netherlands b.l.feringa@rug.nl stefano.crespi@kemi.uu.se; b Faculty of Engineering and Natural Sciences, Tampere University FI-33101 Tampere Finland; c European Laboratory for Non Linear Spectroscopy (LENS) via N. Carrara 1 50019 Sesto Fiorentino Italy; d ICCOM-CNR via Madonna del Piano 10, 50019 Sesto Fiorentino FI Italy; e Van't Hoff Institute for Molecular Sciences, University of Amsterdam Science Park 904 1098 XH Amsterdam The Netherlands; f Institute for Molecules and Materials, FELIX Laboratory, Radboud University Toernooiveld 7c 6525 ED Nijmegen The Netherlands; g Department of Chemistry, Ångström Laboratory, Uppsala University Box 523 751 20 Uppsala Sweden

## Abstract

Correction for ‘A visible-light-driven molecular motor based on barbituric acid’ by Kim Kuntze *et al.*, *Chem. Sci.*, 2023, **14**, 8458–8465, https://doi.org/10.1039/D3SC03090C.

The authors regret that the link included in the data availability statement was incorrect in the original article. The corrected data availability statement is shown below:

The datasets supporting this article have been uploaded as part of the ESI. All cartesian coordinates for all the compounds considered are provided as a separate additional file in a fig-share repository with the following DOI: https://figshare.com/articles/dataset/A_Visible-Light-Driven_Molecular_Motor_Based_on_Barbituric_Acid/23276999.

The Royal Society of Chemistry apologises for these errors and any consequent inconvenience to authors and readers.

## Supplementary Material

