# Supplementary figures and images for: A visible-light-driven molecular motor based on barbituric acid
Source: Chem Sci. 2023 Jul 20;14(32):8458–65. doi: 10.1039/d3sc03090c (PMC10430646; doi:10.1039/d3sc03090c)

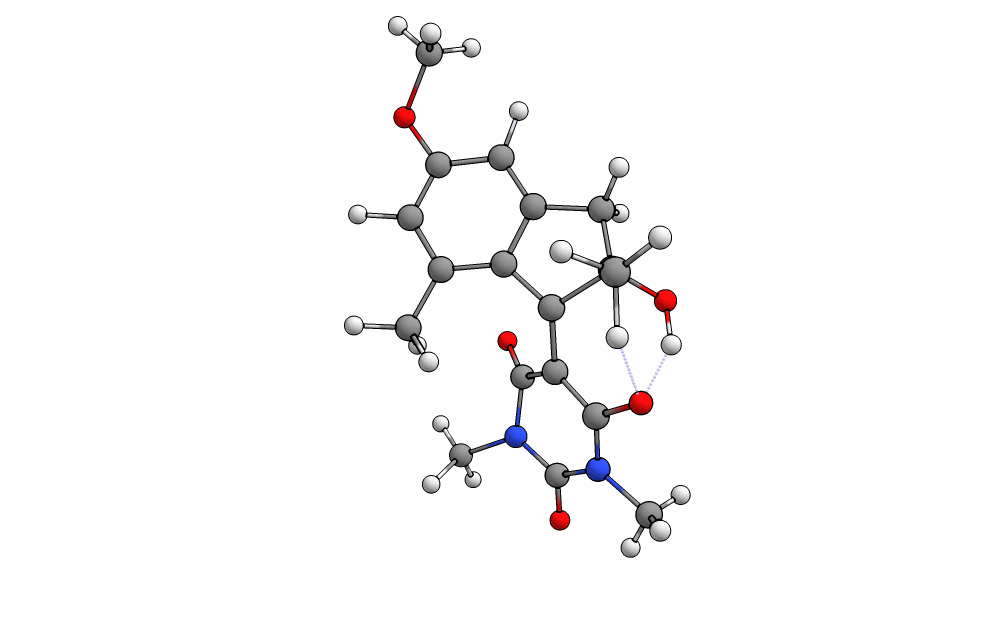

Supplement: SC-014-D3SC03090C-s001 [file SC-014-D3SC03090C-s001.gif]
