# Supplementary material for: A visible-light-driven molecular motor based on barbituric acid
Source: Chem Sci. 2023 Jul 20;14(32):8458–65. doi: 10.1039/d3sc03090c (PMC10430646; doi:10.1039/d3sc03090c)
Supplement: SC-014-D3SC03090C-s002 [file SC-014-D3SC03090C-s002.pdf]

Electronic Supplementary Material (ESI) for Chemical Science.
This journal is © The Royal Society of Chemistry 2023

Supporting Information

A Visible-Light-Driven Molecular Motor Based on Barbituric Acid

Kim Kuntze[†a,b], Daisy R. S. Pooler[†a], Mariangela Di Donato[c,d], Michiel F. Hilbers[e], Pieter van der Meulen[a], Wybren Jan Buma[e,f], Arri Priimagi[b], Ben L. Feringa*[a] and Stefano Crespi*[a,g]

[a] Stratingh Institute for Chemistry, University of Groningen, Nijenborgh 4, 9746 AG Groningen, The Netherlands

[b] Faculty of Engineering and Natural Sciences, Tampere University of Technology, FI-33101 Tampere, Finland

[c] European Laboratory for Non Linear Spectroscopy (LENS) via N. Carrara 1, 50019 Sesto Fiorentino, Italy

[d] ICCOM-CNR, via Madonna del Piano 10, 50019 Sesto Fiorentino (FI), Italy

[e] Van't Hoff Institute for Molecular Sciences, University of Amsterdam, Science Park 904, 1098 XH, Amsterdam, The Netherlands

[f] Institute for Molecules and Materials, FELIX Laboratory, Radboud University, Toernooiveld 7c, 6525 ED Nijmegen, The Netherlands

[g] Department of Chemistry, Ångström Laboratory, Uppsala University, Box 523, 751 20 Uppsala, Sweden

† These authors contributed equally

Email: stefano.crespi@kemi.uu.se; b.l.feringa@rug.nl

Table of Contents

1. General procedures 3
2. Preparation and characterisation of compounds 4
   2.1 Reaction studies 9
3. NMR studies of rotation cycle........ 11
4. X-ray structure of $\mathbf{1_S}$ 14
5. SFC separation of stereoisomers........ 16
6. Transient absorption spectroscopy........ 17
7. Computational details 34
8. References 39
9. Appendix........ 40

1. General procedures

All reactions involving air sensitive reagents were performed under an N_2 atmosphere. Solvents were degassed by purging with N_2 for a minimum of 30 min or by three freeze-pump-thaw cycles. Reagents were purchased from Sigma Aldrich, Fluorochem or TCI Europe and were used without further purification. Solvents were reagent grade and used without prior water removal unless otherwise indicated. Anhydrous solvents were obtained from a solvent purification system (MBraun SPS-800). Flash column chromatography was performed on silica gel (Merck, type 9385, 230–400 mesh) or on a Biotage® Selekt system using Biotage® Sfär column cartridges. Semi-preparative supercritical fluid chromatography (SFC) was carried out on a Thar Technologies Inc. SFC (Waters) system, equipped with fluid delivery module (FDM10-1), an autosampler (a modified Alias 840), a semi-prep column oven, PDA detector, back-pressure regulator (ABPR20), heat-exchanger, and a fraction collector (modified Thar SFC-FC). Thin layer chromatography (TLC) was carried out on aluminium sheets coated with silica gel 60 F254 (Merck). Compounds were visualised with a UV lamp (254 nm) and/or by staining with $KMnO_4$ or CAM.

^{1}H and ^{13}C NMR spectra were recorded on a Varian Mercury-Plus 400 or a Bruker Avance 600 NMR spectrometer at 298 K unless otherwise indicated. PSS studies were performed on a Varian Unity Plus 500 NMR spectrometer. Chemical shifts are given in parts per million (ppm) relative to the residual solvent signal ($CDCl_3$ = δ 7.26 for ^{1}H, δ 77.2 for ^{13}C; CD_2Cl_2 = δ 5.32 for ^{1}H, δ 53.8 for ^{13}C; d_4-methanol = δ 3.31 for ^{1}H; d_8-THF = δ 5.02 for ^{1}H, δ 67.2 for ^{13}C). Multiplets in ^{1}H NMR spectra are designated as follows: s (singlet), d (doublet), t (triplet), q (quartet), p (pentet), m (multiplet), br (broad). High resolution mass spectrometry (HRMS, ESI+) was performed on an LTQ Orbitrap XL spectrometer. Ultra-Performance Liquid Chromatography tandem Mass Spectrometry (UPLC-MS) was performed on a Waters Acquity UPLC-MS with Tandem Quadrupole (TQD) fitted with a BEH (bridged ethyl siloxane/silica hyrid) C_{18} column (1.7 μm, 2.1× 50 mm). The UPLC-MS measurements were taken with ESI+, 50-1000 *m*/*z* using H_2O:MeCN as co-solvents. UV-Vis absorption spectra were recorded on an Agilent 8453 UV-Vis Diode Array System, equipped with a Quantum Northwest Peltier controller, in 10 mm quartz cuvettes. Irradiation experiments were performed using LEDs obtained from Thorlabs Incorporated.

2. Preparation and characterisation of compounds

5-methoxy-2,7-dimethyl-2,3-dihydro-1H-inden-1-one (S1)

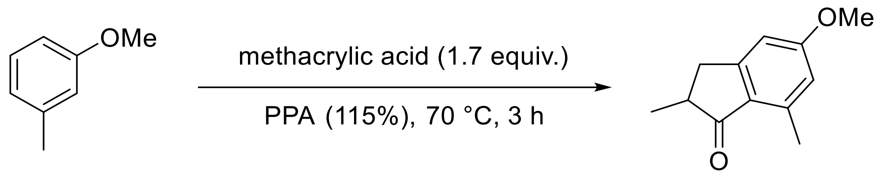

To a wide-necked 1 L recovery flask equipped with a mechanical stirrer, polyphosphoric acid (PPA, 115% H_3PO_4, 60 mL) was added, mechanically stirred and heated to 70 °C. Subsequently, methacrylic acid (25.4 mL, 300 mmol, 1.7 equiv.) and 3-methylanisole (22.4 mL, 178 mmol, 1.0 equiv.) were added, and the reaction mixture was stirred at 70 °C for 3 h. The mixture was allowed to cool to room temperature, and was quenched by adding ice water (~500 mL) and mechanically stirred overnight. The mixture was then filtered, and the filter cake was dissolved in EtOAc (200 mL), then washed with saturated aq. $NaHCO_3$ (2 x 50 mL) and brine (50 mL), dried over $MgSO_4$ and concentrated *in vacuo* to a yellow crude mixture. The crude product was purified by flash column chromatography (SiO_2, pentane:EtOAc 95:5 → 80:20) to yield a yellow oil, to which was added MeOH (~5 mL) and crystallised in the freezer overnight. The crystals were filtered and washed with pentane, to yield **S1** (7.9 g, 42 mmol, 23%) as white crystals. **^{1}H NMR** (300 MHz, $CDCl_3$) δ 6.70 (s, 1H, Ar*H*), 6.62 (s, 1H, Ar*H*), 3.85 (s, 3H, OC*H₃*), 3.41 – 3.09 (m, 1H, C*H*CH₃), 2.65 (m, overlaps ArC*H₃*, 2H, C*H₂*), 2.60 (s, overlaps CH_2, 3H, ArC*H₃*), 1.27 (dd, *J* = 7.3, 0.7 Hz, 3H, CHC*H₃*); **^{13}C NMR** (101 MHz, $CDCl_3$) δ 208.7, 164.6, 157.3, 141.1, 127.5, 116.5, 107.4, 55.6, 42.5, 34.9, 18.6, 16.8; **HRMS** (ESI+, *m/z*) calcd for $C_{12}H_{14}O_2$ $[M+H]^+$ = 191.1067, found 191.1065.

1-methyl-3-phenylurea (S2)

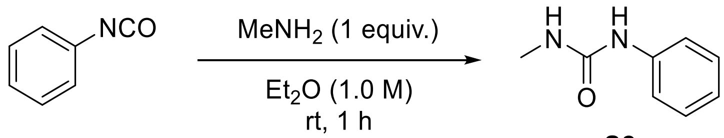

S2

Methylamine (2 M in THF, 10 mL, 20 mmol, 1 equiv.) was dissolved in diethyl ether (20 mL, 1.0 M) under ambient conditions. Phenyl isocyanate (2.2 mL, 20 mmol, 1 equiv.) was added, causing immediate white precipitation. The reaction mixture was stirred at room temperature for 1 h, filtered and the precipitate washed with diethyl ether to yield **S2** (2.17 g, 72%) as a white crystalline product. **^{1}H NMR** (400 MHz, DMSO-d_6) δ 8.45 (s, 1H, ArCN*H*), 7.41 – 7.34 (m, 2H, NHCAr*H*), 7.24 – 7.15 (m, 2H, ArAr*H*), 6.87 (tt, *J* = 7.3, 1.2 Hz, 1H, Ar*H*), 5.97 (q, *J* = 4.6 Hz, 1H, CH_3NH), 2.63 (d, J = 4.6 Hz, 3H, NHCH_3); **^{13}C NMR** (101 MHz, DMSO-d_6) δ 155.8, 140.6, 128.6, 120.9, 117.6, 26.2.

1-methyl-3-phenylpyrimidine-2,4,6(1H,3H,5H)-trione (S3)

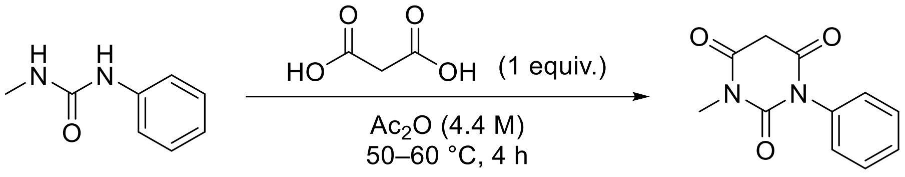

S2 **S3**

To a 100 mL round-bottom flask, 1-methyl-3-phenylurea (**S2**, 1.00 g, 6.66 mmol, 1 equiv.), malonic acid (693 mg, 6.66 mmol, 1 equiv.) and acetic anhydride (1.5 mL, 4.4 M) were added. The flask was subsequently attached to a rotary evaporator, and was stirred and heated at 50 °C in until all solids had dissolved. Keeping the same temperature, the solution was then subjected to a pressure of 25 mbar. After 2 h, the temperature was raised to 60 °C. After 4 h in total, most of the acetic anhydride had evaporated. The crude reaction mixture was purified by column chromatography (SiO_2, 0–10% MeOH in CH_2Cl_2) to yield **5** (665 mg, 46%) and unreacted **S2** (270 mg, 27%) as white solids. **^{1}H NMR** (400 MHz, $CDCl_3$) δ 7.55 – 7.41 (m, 3H, NHCAr*H* + Ar*H*), 7.23 – 7.15 (m, 2H, ArAr*H*), 3.85 (s, 2H, CH_2), 3.36 (s, 3H, NCH_3); **^{13}C NMR** (101 MHz, $CDCl_3$) δ 164.6, 164.4, 151.5, 134.0, 129.5, 129.3, 128.3, 77.3, 77.0, 76.7, 39.9, 28.7.

5-(2-hydroxy-5-methoxy-2,7-dimethyl-2,3-dihydro-1H-inden-1-ylidene)-1,3-dimethyl pyrimidine-2,4,6(1H,3H,5H)-trione (1_S)

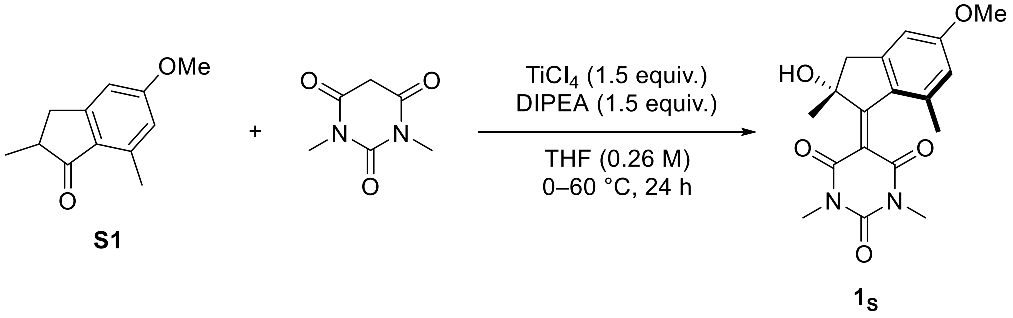

An oven-dried 10 mL crimp-top vial under N_2 atmosphere was charged with **S1** (200 mg, 1.05 mmol, 1.0 equiv.), which was dissolved in dry THF (2 mL, 0.53 M) and then cooled down to 0 °C in an ice bath. $TiCl_4$ (0.17 mL, 1.58 mmol, 1.5 equiv.) was then added dropwise and stirred for 5 min during which the solution turned yellow. Meanwhile, barbituric acid (246 mg, 1.58 mmol, 1.5 equiv.) was dissolved in dry THF (2 mL, 0.79 M), which was subsequently added to the previous solution and stirred for 30 min at 0 °C (0.26 M). The solution turned red. DIPEA (0.28 mL, 1.58 mmol, 1.5 equiv.) was then added dropwise, turning the mixture dark red. The reaction mixture was heated to 60 °C and was stirred for 24 h under nitrogen. It was then quenched with saturated aq. NH_4Cl (10 mL) and extracted with EtOAc (3 x 20 mL). The yellow organic phase was washed with saturated aq. $NaHCO_3$ (2 x 20 mL), water, and brine, dried on $MgSO_4$ and concentrated *in vacuo*. The crude product was then purified by flash column chromatography (SiO_2, 0–40% EtOAc in pentane) and crystallised from EtOAc/pentane to yield **1** (31 mg, 14%) as orange crystals and recovered **S1** (130 mg, 65%) as well as traces of an elimination product **E1** (see section 2.1). **^{1}H NMR** (400 MHz, $CDCl_3$) δ 6.70 (t, *J* = 1.8 Hz, 1H), 6.68 (d, *J* = 2.4 Hz, 1H), 5.57 (s, 1H), 3.88 (s, 3H), 3.50 (d, *J* = 14.7 Hz, 1H), 3.35 (s, 3H), 3.06 (d, *J* = 14.7 Hz, 1H), 2.20 (s, 3H), 1.55 (s, 3H). **^{13}C NMR** (101 MHz, $CDCl_3$) δ 191.4, 165.6, 163.8, 160.7, 153.0, 151.2, 145.3, 131.5, 116.4, 110.7, 107.4, 87.7, 55.7, 49.4, 28.8, 28.7, 26.5, 21.6; **HRMS** (ESI–, *m/z*) calcd for $C_{18}H_{20}N_2O_5$ $[M–H]^-$ = 343.1300, found 343.1293.

5-methoxy-2,7-dimethyl-1H-inden-1-one (E1)

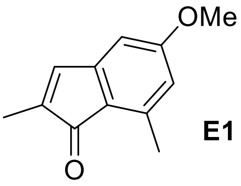

E1

^{1}H NMR (400 MHz, $CDCl_3$) δ 6.92 (q, *J* = 1.8 Hz, 1H, CH_3C=C*H*), 6.35 (d, *J* = 2.2 Hz, 1H, Ar*H*), 6.25 (d, *J* = 2.2 Hz, 1H, Ar*H*), 3.80 (s, 3H, OC*H*$_3$), 2.46 (s, 3H, ArC*H*$_3$), 1.84 (d, *J* = 1.8 Hz, 3H, C*H*$_3$C=CH); **^{13}C NMR** (101 MHz, $CDCl_3$) δ 198.4, 164.0, 148.1, 140.1, 139.8, 138.1, 120.5, 111.9, 108.5, 55.6, 17.6, 10.3.; **HRMS** (ESI+, *m/z*) calcd for $C_{12}H_{12}O_2$ $[M+H]^+$ = 189.0910, found 180.0910.

5-(2-hydroxy-5-methoxy-2,7-dimethyl-2,3-dihydro-1H-inden-1-ylidene)-1-methyl-3-phenyl pyrimidine-2,4,6(1H,3H,5H)-trione (2)

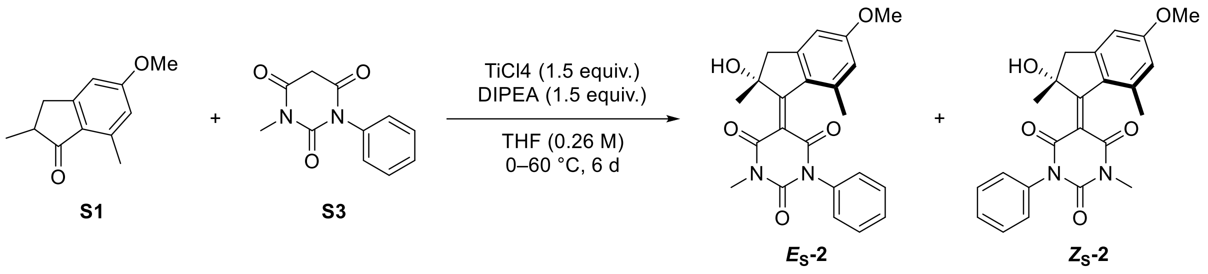

An oven-dried 10 mL crimp-top vial under N_2 atmosphere was charged with **S1** (200 mg, 1.05 mmol, 1.0 equiv.), dissolved in dry THF (2 mL, 0.53 M) under nitrogen and cooled down to 0 °C in an ice bath. $TiCl_4$ (0.173 mL, 1.58 mmol, 1.5 equiv.) was then added dropwise and stirred for 5 min during which the solution turned yellow. Meanwhile, **S3** (252 mg, 1.16 mmol, 1.1 equiv.) was dissolved in dry THF (2 mL, 0.58 M), which was subsequently added to the previous solution and stirred for 30 min at 0 °C. The solution turned red. DIPEA (0.28 mL, 1.58 mmol, 1.5 equiv.) was then added dropwise, turning the mixture dark red. The reaction mixture was heated to 60 °C and was stirred for 6 d under nitrogen. It was then quenched with saturated aq. NH_4Cl (10 mL) and extracted with EtOAc (3 x 20 mL). The yellow organic phase was washed with saturated aq. $NaHCO_3$ (2 x 20 mL), water, and brine, dried on $MgSO_4$ and concentrated *in vacuo*. The crude product was then purified by flash column chromatography (SiO_2, 0–40% EtOAc in pentane) and crystallised from EtOAc/pentane to yield **2** (20 mg, 5%) as yellow crystals, and as a 1:1 mixture of ***E***$_S$**-2** and ***Z***$_S$**-2**. **^{1}H NMR** (600 MHz, $CDCl_3$) δ 7.54 (t, *J* = 7.7 Hz, 2H, **2a** + **2c**), 7.51 – 7.3 (m, 4H, **2a** + **2c**), 7.32 – 7.28 (m, 2H, **2a** + **2c**), 7.19 (d, *J* = 7.6 Hz, 2H, **2a** + **2c**), 6.69 – 6.65 (m, 2H, **2a** + **2c**), 6.60 (d, *J* = 2.3 Hz, 2H, **2a** + **2c**), 5.57 (s, 1H, **2c**), 5.27 (s, 1H, **2a**), 3.90 (s, 3H, **2a**), 3.84 (s, 3H, **2c**), 3.51 (dd, *J* = 14.7, 3.7 Hz, 2H, **2a** + **2c**), 3.47 (s, 3H, **2c**), 3.39 (s, 3H, **2a**), 3.07 (dd, *J* = 14.7, 8.9 Hz, 2H, **2a** + **2c**), 2.29 (s, 3H, **2a**), 2.23 (s, 3H, **2c**), 1.60 (s, 3H, **2c**), 1.51 (s, 3H, **2a**). **HRMS** (ESI+, *m*/*z*) calcd for $C_{18}H_{20}N_2O_5$ $[M+Na]^+$ = 429.1421, found 429.1417.

2-chloro-5-methoxy-2,7-dimethyl-2,3-dihydro-1H-inden-1-one (S4)

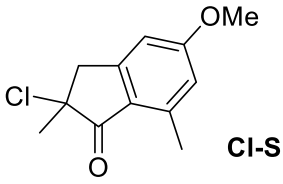

Cl-S1

Diisopropylamine (DIPA, 265 µL, 1.90 mmol, 1.2 equiv.) was dissolved in dry THF (2 mL,) under a nitrogen atmosphere and cooled down to –78 °C. *n*-BuLi (1.6 M in hexane, 1.09 mL, 1.74 mmol, 1.1 equiv.) was then added and the reaction mixture was stirred for 40 minutes. Meanwhile, **S1** (300 mg, 1.58 mmol, 1.0 equiv.) was separately dissolved in dry THF (3 mL, 0.53 M) under a nitrogen atmosphere and cooled down to –78 °C. The first solution, now containing the freshly prepared LDA, was subsequently added to this solution, followed by the addition of sulphuryl chloride (SO_2Cl_2, 140 µL, 1.74 mmol, 1.1 equiv.). Gas evolution was observed. The reaction mixture was slowly allowed to reach room temperature and stirred until no gas evolution was observed. The reaction was then quenched by the addition of water and extracted with EtOAc. The organic layer was washed with water, brine, dried with $MgSO_4$, filtered and concentrated *in vacuo*. The crude mixture was purified by flash column chromatography (SiO_2, 0–50% CH_2Cl_2 in pentane) to yield **Cl-S1** (49 mg, 14%) as a colourless oil. **^{1}H NMR** (400 MHz, $CDCl_3$) δ 6.69 (s, 1H, Ar*H*), 6.67 (s, 1H, Ar*H*), 3.87 (s, 3H, OC*H*$_3$), 3.55 (d, *J* = 17.8 Hz, 1H, C*H*$_2$), 3.34 (d, *J* = 17.8 Hz, 1H, C*H*$_2$), 2.62 (s, 3H, ArC*H*$_3$), 1.76 (s, 3H, CClC*H*$_3$). **^{13}C NMR** (101 MHz, $CDCl_3$) δ 199.3, 165.7, 153.4, 143.0, 124.0, 117.3, 107.5, 67.8, 55.8, 45.3, 26.8, 18.7.; **HRMS** (ESI+, *m*/*z*) calcd for $C_{12}H_{13}O_2Cl$ $[M+H]^+$ = 225.0677, found 225.0677.

5-methoxy-2,7-dimethyl-2,3-dihydro-1H-inden-1-one-^{18}O (^{18}O-S1)

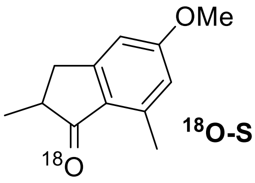

^{18}O-S1

S1 (50 mg, 0.263 mmol), $^{18}OH_2$ (50 µL, 2.75 mmol) and hydrogen chloride (2 M in diethyl ether, 15 µL, 0.030 mmol) were dissolved in dry tetrahydrofuran (250 µL) under a nitrogen atmosphere and stirred at 65 °C overnight. The conversion was followed with UPLC-MS. After 16 hours sodium bicarbonate and sodium sulphate were added, the reaction mixture was diluted with EtOAc, filtered and concentrated under reduced pressure to yield an oxygen-18-enriched batch of **S1** containing ca. 75% of ^{18}O (based on peak intensity ratios in MS). The NMR spectrum was identical to that measured before for non-labelled **S1**. **^{1}H NMR** (400 MHz, $CDCl_3$) δ 6.70 (t, *J* = 1.8 Hz, 1H), 6.68 (d, *J* = 2.4 Hz, 1H), 5.57 (s, 1H), 3.88 (s, 3H), 3.50 (d, *J* = 14.7 Hz, 1H), 3.35 (s, 3H), 3.06 (d, *J* = 14.7 Hz, 1H), 2.20 (s, 3H), 1.55 (s, 3H). **^{13}C NMR** (101 MHz, $CDCl_3$) δ 191.4, 165.6, 163.8, 160.7, 153.0, 151.2, 145.3, 131.5, 116.4, 110.7, 107.4, 87.7, 55.7, 49.4, 28.8, 28.7, 26.5, 21.6; **HRMS** (ESI+, *m*/*z*) calcd for $C_{12}H_{14}O^{18}O$ $[M+H]^+$ = 193.1109, found 193.1107.

2.1 Reaction studies

The Knoevenagel condensation between **S1** and barbituric acid was studied to gain insight into the mechanism. When standard conditions were used, we isolated 15% of **1**, traces of the elimination product **2** and mostly unreacted **S1** (65%). In addition, side products resulting from dimerisation of THF[1] were prominent. Because of these impurities, isolating pure **1** required crystallisation after column chromatography. In addition, the reaction was far from complete even after long reaction times. Thus, isolated or otherwise determined absolute yields would not be a reasonable way to study the effect of different reaction parameters. Instead, we studied the reaction by carrying out parallel reactions, one of them having the standard conditions and changing one parameter at a time in the other(s). The yield was then estimated from the crude ^{1}H NMR for each reaction (comparing the OH signal of **1** and one of the Ar–H signals of **S1**) and compared between the reference reaction and the other(s). Although we acknowledge the error in NMR integrals due to shimming differences, we deemed this approach most viable and to be accurate enough to determine whether there is a significant change in the reaction outcome or not. The results are compiled in Table S1.

Table S1: Synthetic reaction studies towards motor **1**. [a]**S1** (1 eq.) and 1,3-dimethylbarbituric acid (1.5 equiv.) in dry THF (0.25 M with respect to S1) at 0 °C before sequential additions of TiCl4 (1.5 equiv.) and DIPEA (1.5 equiv.). Then stirred at 60 °C under a N_2 atmosphere for 24 hours, quenched with an aqueous NH_4Cl solution, extracted with EtOAc, washed with $NaHCO_3$, H_2O and brine, dried with $MgSO_4$ and concentrated at 40 °C under reduced pressure. [b]Lesser side reactions may have occurred but no products could be isolated. [c]No differences in ^{1}H NMR or MS spectra.

Entry	Deviation from standard conditions (s.c.)[a]	Outcome
1	No $TiCl_4$	No reaction[b]
2	No DIPEA	No reaction[b]
3	$TiCl_4$ 5 eq.	More THF impurities
4	DIPEA 5 eq.	More **E1**
5	BCl_2 instead of $TiCl_4$, DCM, -78 to 0 °C	Traces of **1**
6	BF_3•OEt_2 instead of $TiCl_4$, DCM, -78 to 0 °C	No reaction[b]
7	DCM as solvent	No reaction[b]
8	Et_2O as solvent	Traces of **1**
9	Added activated molecular sieves (4 Å)	No difference to s.c.
10	Freeze-pump-thaw-degassed solvent, argon atmosphere	No difference to s.c.
11	Quenched with distilled H_2O	No difference to s.c.
12	Quenched with D_2O	No difference to s.c.
13	Quenched with MeOH	No difference to s.c.
14	Ambient lighting blocked	No difference to s.c.
15	**Cl-S1** instead of **S1**	No reaction[b]
16	**^{18}O-S1** instead of **S1**	No difference to s.c.[c]
17	No work-up	Mass +16 cf. **1**
18	**^{18}O-S1** instead of **S1**, no work-up	Mass +16 cf. **1**

The reactions featuring **^{18}O-S1** (entries 16 and 18) were carried out using a mixture containing **^{18}O-S1** and **S1** in ca. 3:1 ratio. The mass spectra obtained directly after quenching with aqueous NH_4Cl and after full work-up, starting both from **S1** and the ^{18}O-enriched mixture, are shown in Figure S1.

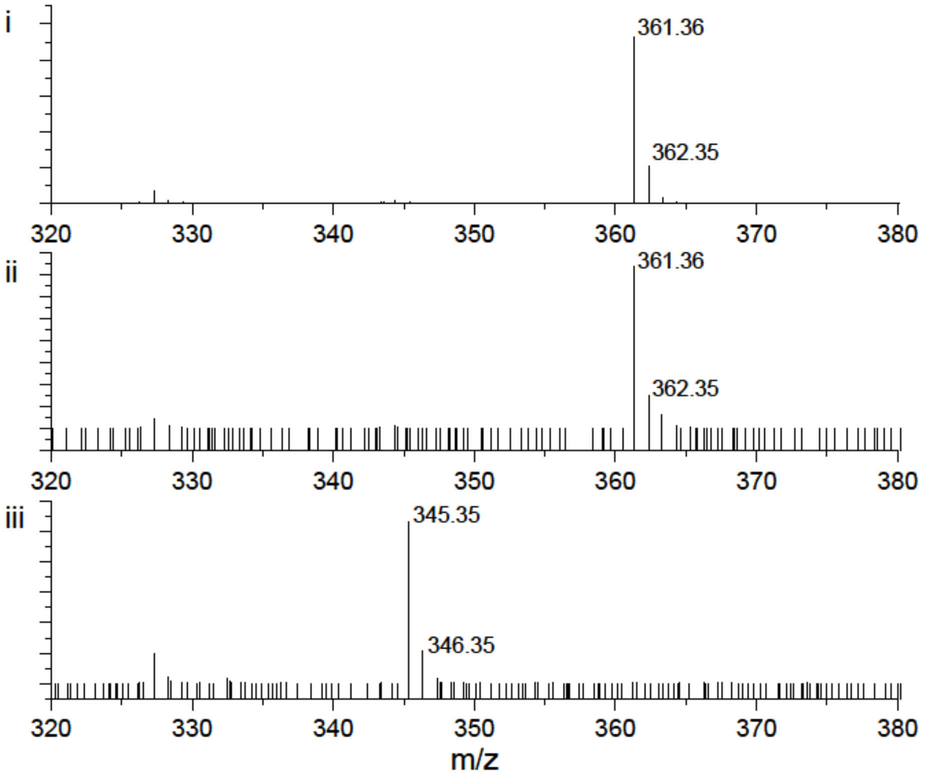

Figure S1: UPLC-MS data of reactions entries (16–18) featuring **^{18}O-S1**. i) Reaction mixture with **S1** after quenching with aq. NH_4Cl (entry 17); ii) reaction mixture with **^{18}O-S1** after quenching aq. NH_4Cl (entry 18); iii) reaction mixture with **^{18}O-S1** after full work-up (entry 16).

3. NMR studies of rotation cycle

A solution (2 x 10^{-3} M) of ***E*$_S$-1** isomer of motor **1** in degassed CD_3OD (sparged with N_2 for 30 min) were prepared and transferred into an NMR tube which was subsequently fitted with a glass optic fibre for *in situ* irradiation studies. The sample was place in a Varian Unity Plus 500 MHz NMR and cooled to –90 °C. ^{1}H NMR spectra were recorded before irradiation, while irradiating at –90 °C, and at –90 °C after heating up to –45 °C for 15 min (for the THI step).

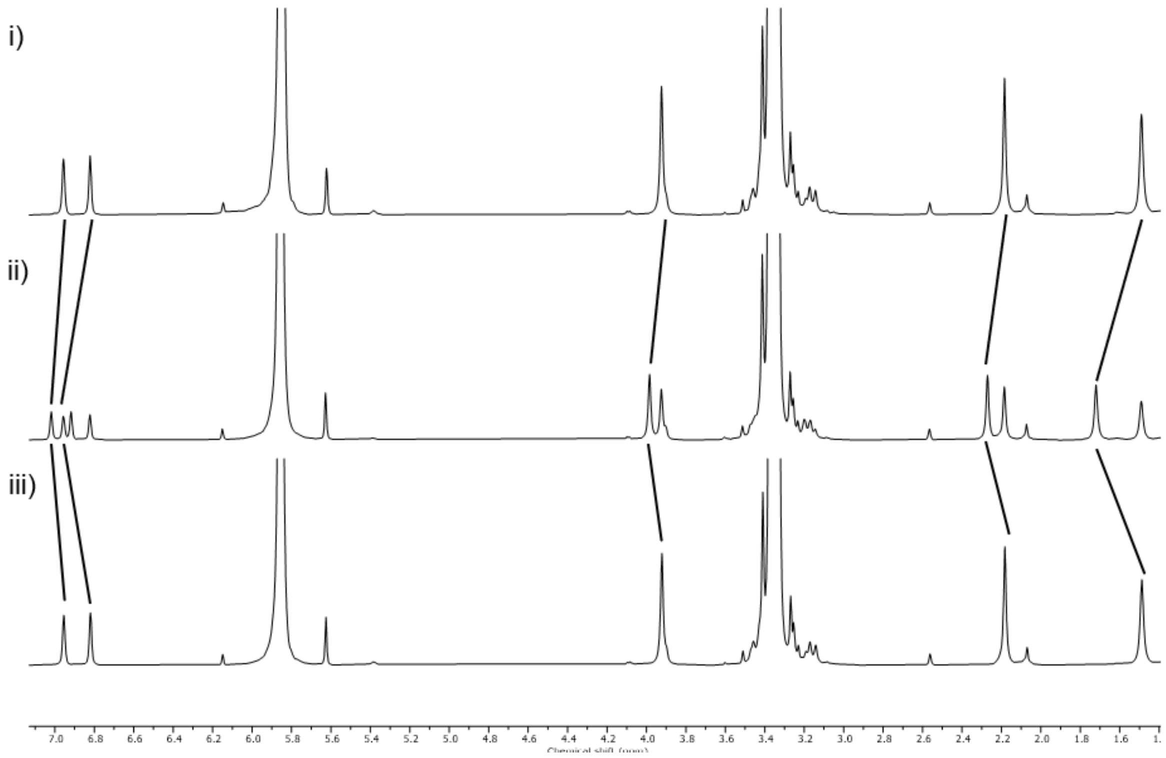

Figure S2: ^{1}H NMR irradiation studies of **1** in CD_3OD (c = 2.0 x 10^{-3} M). i) **1$_S$** before irradiation; ii) PSS$_{420}$ (63:37 **1$_S$** : **1$_M$**); iii) THI, –45 °C, 15 min

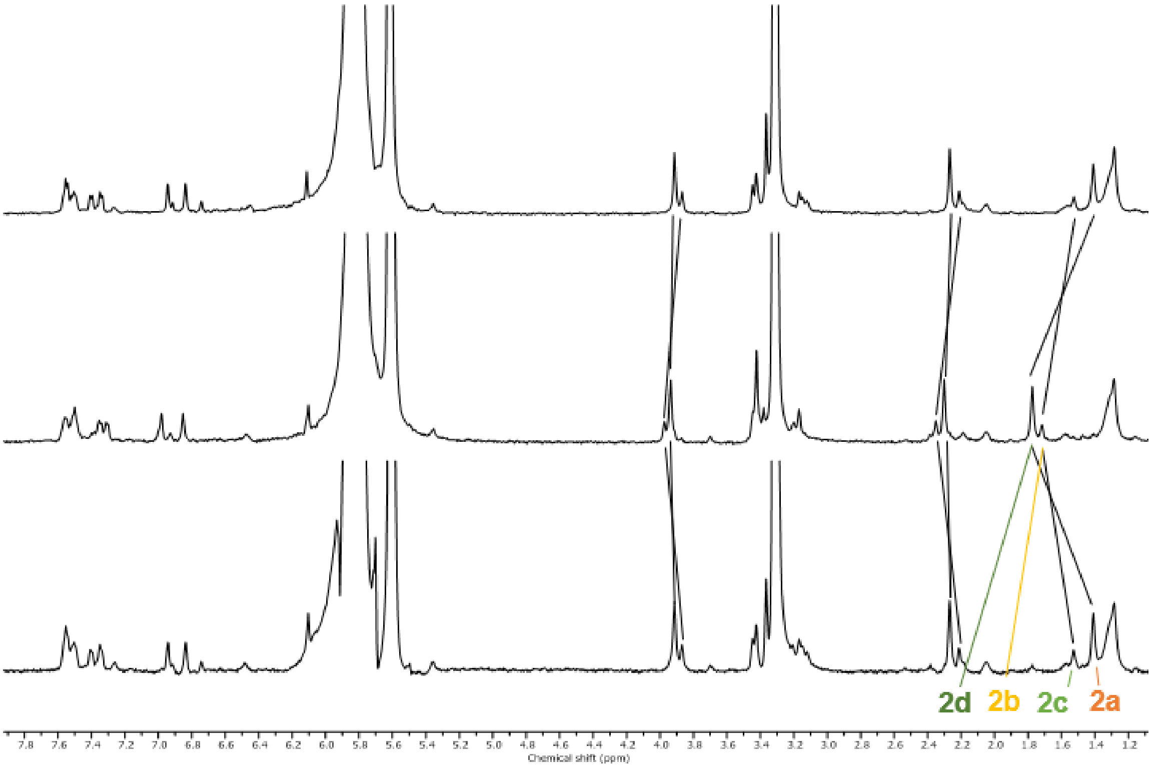

Figure S3: ^{1}H NMR irradiation studies of **2** in CD_3OD (c = ~1.0 x 10^{-3} M). i) **2a** and **2c** before irradiation; ii) PSS_{420} (8:5:22:65, **2a**:**2b**:**2c**:**2d**); iii) THI, –90 °C, 70 min (73:27, **2a**:**2c**).

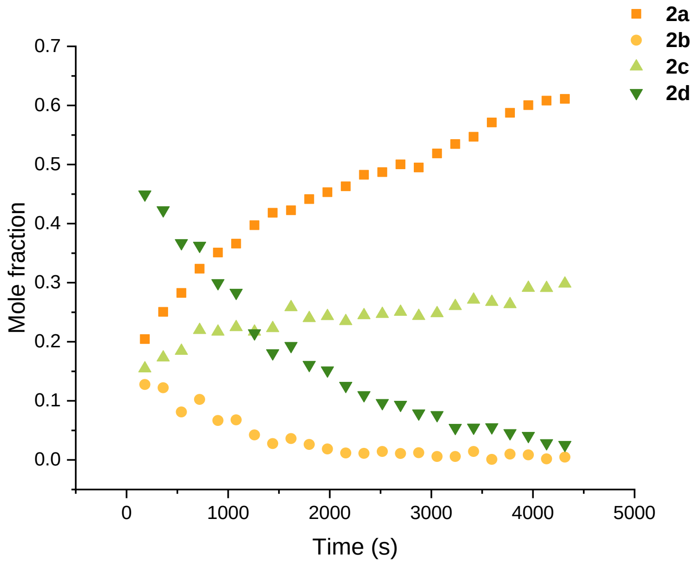

Figure S4: Kinetic traces of the THI of **2** at –90 °C.

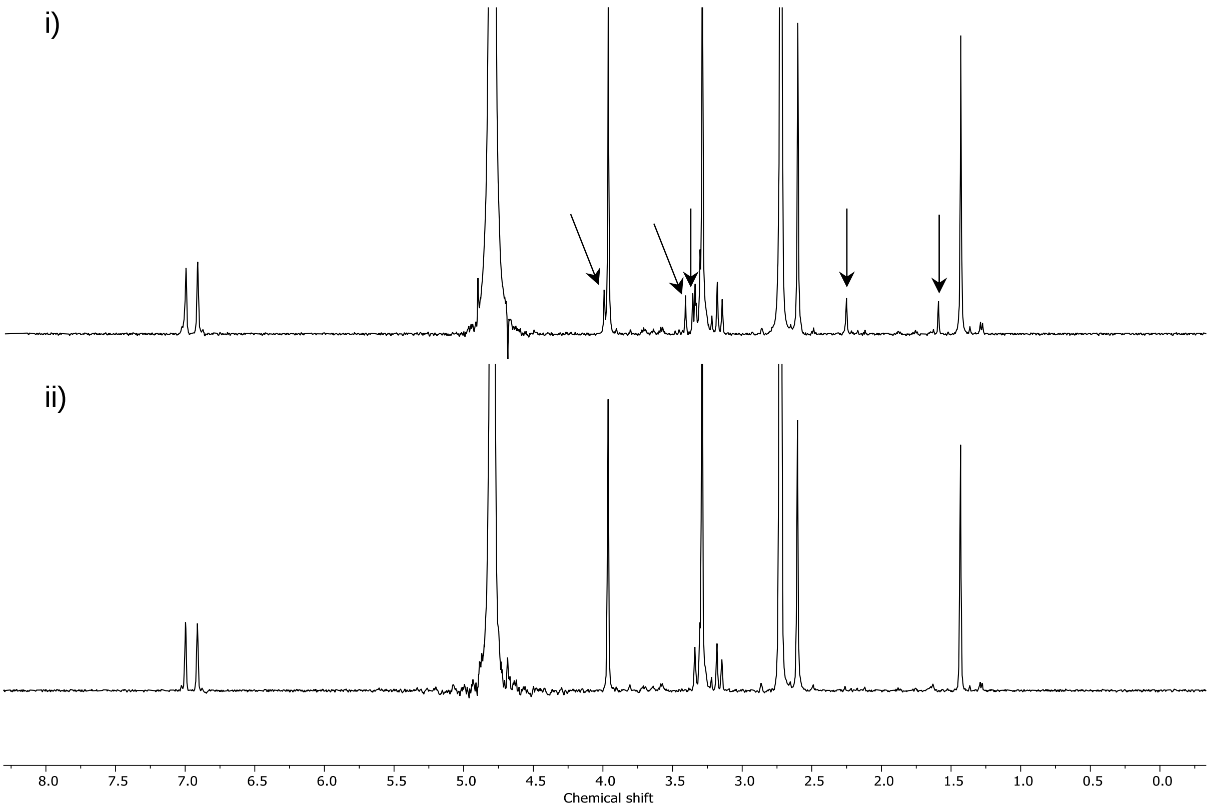

Figure S5: ^{1}H NMR spectra of compound **1**$_S$ in D_2O:DMSO-d_6 (9:1) showing degradation of the compound in water. i) After 1 h, signals of compound **1**$_S$ shown with black arrows; ii) after 24 h, full degradation of compound **1**$_S$.

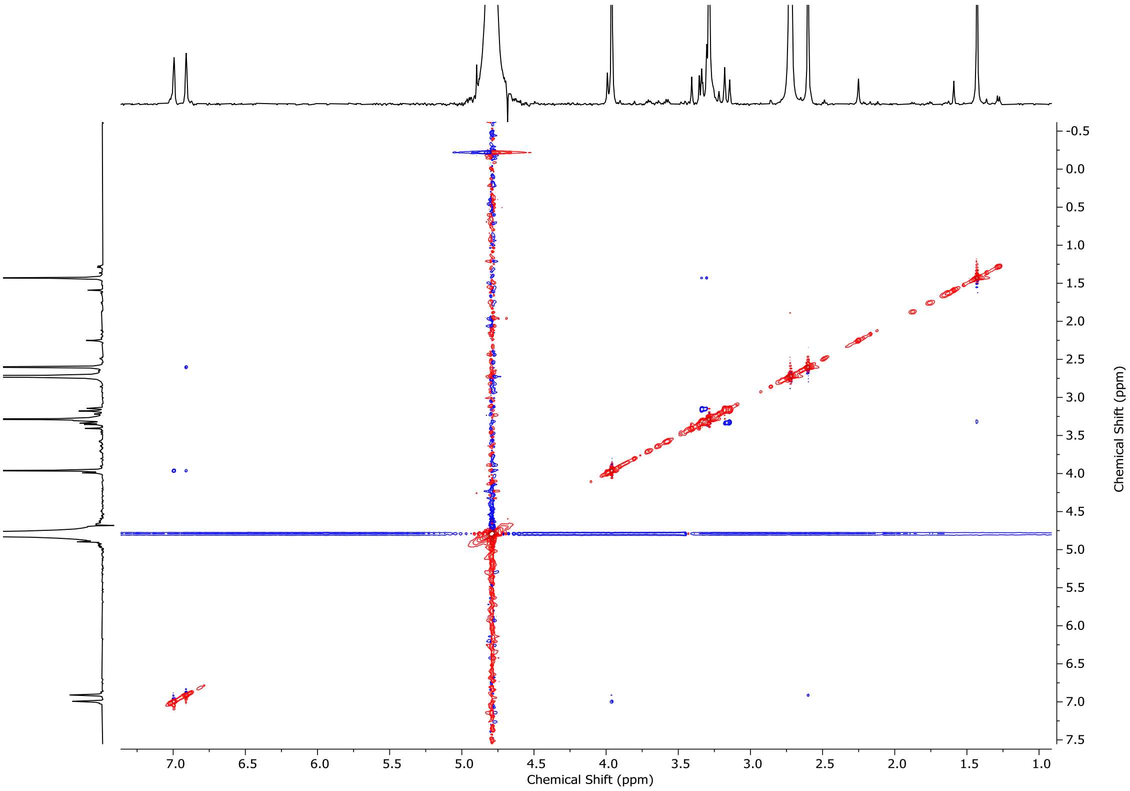

Figure S6: 2D NOESY of compound **1**$_S$ in D_2O:DMSO-d_6 (9:1) showing degradation of the compound in water.

4. X-ray structure of **1**$_S$

A single-crystal of **1**$_S$ was mounted on a cryoloop and placed in the nitrogen stream (100 K) of a Bruker-AXS D8 Venture diffractometer. Data collection and processing was carried out using the Bruker APEX4 software.[2] A multi-scan absorption correction was applied, based on the intensities of symmetry-related reflections measured at different angular settings (*SADABS*).[3] The structure was solved using *SHELXT*[4] and refinement was performed using *SHELXL*.[5] The hydrogen atoms were generated by geometrical considerations, constrained by idealised geometries and allowed to ride on their carrier atoms with an isotropic displacement parameter related to the equivalent displacement parameter of their carrier atoms. No A- or B-level alerts were raised by CheckCIF for the fully refined structure.

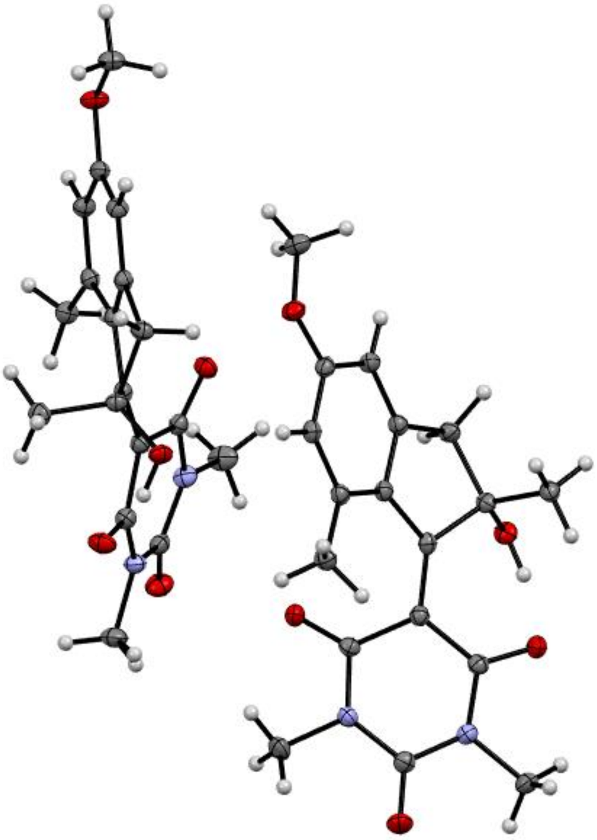

Figure S7: ORTEP image (ellipsoid at 50% probability) of **1**$_S$.

Table 1: Crystal data and structure refinement for **1s**.

Empirical formula	$C_{18}H_{20}N_2O_5$
Formula weight	344.36
Temperature/K	100.00
Crystal system	monoclinic
Space group	$P2_1/n$
a/Å	10.1319(7)
b/Å	25.5792(18)
c/Å	12.5674(9)
α/°	90
β/°	90.459(2)
γ/°	90
Volume/Å^3	3256.9(4)
Z	8
ρ_{calc}g/cm^3	1.405
μ/mm^{-1}	0.859
F(000)	1456.0
Crystal size/mm^3	0.31 × 0.281 × 0.2
Radiation	CuKα (λ = 1.54178)
2Θ range for data collection/°	6.912 to 144.43
Index ranges	-10 ≤ h ≤ 12, -31 ≤ k ≤ 31, -15 ≤ l ≤ 15
Reflections collected	85279
Independent reflections	6416 [R_{int} = 0.0864, R_{sigma} = 0.0408]
Data/restraints/parameters	6416/0/362
Goodness-of-fit on F^2	1.018
Final R indexes [I>=2σ (I)]	R_1 = 0.0512, wR_2 = 0.1196
Final R indexes [all data]	R_1 = 0.0619, wR_2 = 0.1223
Largest diff. peak/hole / e Å^{-3}	0.55/-0.56

5. SFC separation of stereoisomers

The stereoisomers of motor **2** were separated by semi-preparative supercritical fluid chromatography (SFC) on a Thar Technologies Inc. SFC (Waters) system, equipped with fluid delivery module (FDM10-1), an autosampler (a modified Alias 840), a semi-prep column oven, PDA detector, back-pressure regulator (ABPR20), heat-exchanger, and a fraction collector (modified Thar SFC-FC).

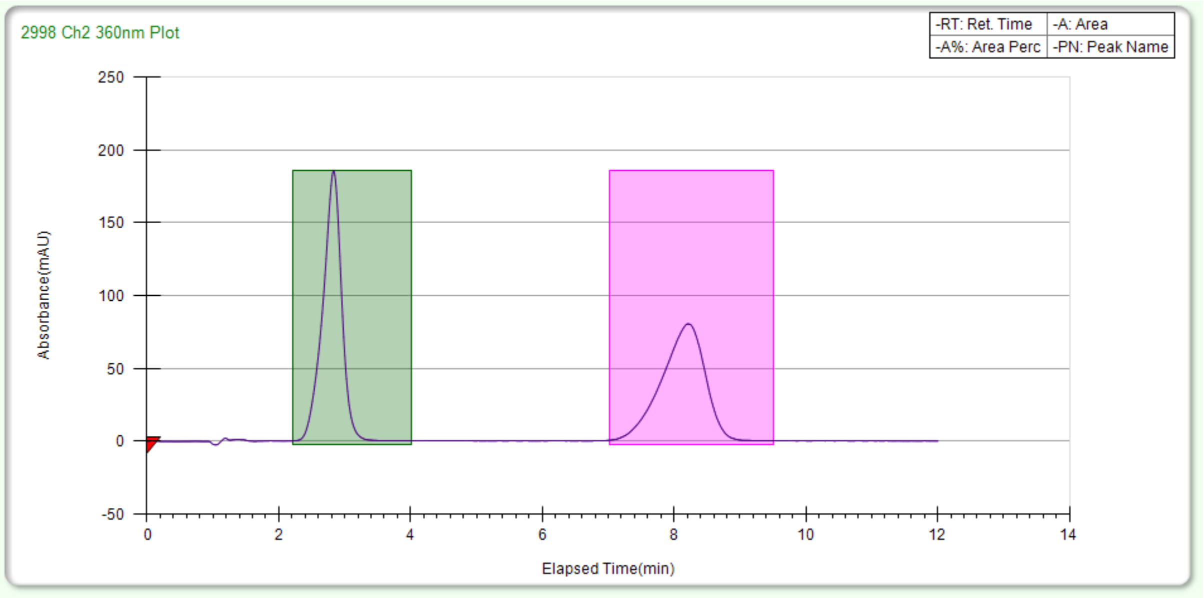

Figure S8: SFC trace of motor ***E*S-2**/***Z*S-2** monitored at 360 nm. Conditions: 10% propan-2-ol, 4.0 mL min^{-1}, 12 min, 40 °C. t_R^1 = 2.8 min; t_R^2 = 8.2 min.

6. Transient absorption spectroscopy

Femtosecond transient absorption spectroscopy

Pump-probe experiments have been performed on a setup based on a regenerative amplifier Ti:sapphire laser (Legend, Coherent), pumped by a Ti:sapphire oscillator (Micra, Coherent). The system produces 40 fs pulses at 800 nm, at 1 KHz repetition rate with an average 3.2 W power. The pump pulses at 400 nm were obtained by second harmonic generation of the fundamental laser output using a 2 mm thick BBO crystal.

The probe beam has been obtained by focusing a small portion of the 800 nm laser radiation on a 3 mm thick calcium fluoride window, generating a white light continuum covering the 375–750 nm spectral window. The withe light is then split in a probe and reference beam using a 50% beam splitter. Pump-probe delays are introduced by sending the fraction of the pulse used to generate the white light to a motorized translation stage. Both pump and probe are spectrally overlapped at the sample position, and after crossing into the sample, the probe and reference beams are sent through a spectrograph coupled to a home-made detector. The sample solution was contained in a 2 mm quartz cuvette, mounted on a movable stage to avoid photodegradation and multiple excitations. The data were analysed by means of singular value decomposition and global analysis, employing the software Glotaran.[6]

The ultrafast excited state behavior of compounds **1** and **2** has been studied by measuring their transient absorption spectra upon excitation at 400 nm. The data have been analyzed using global analysis, employing a linear unidirectional decay scheme and four decay constants, obtaining the EADS (Evolution Associated Difference Spectra) reported in Figure 1 b) and 1 d) for the two compounds.

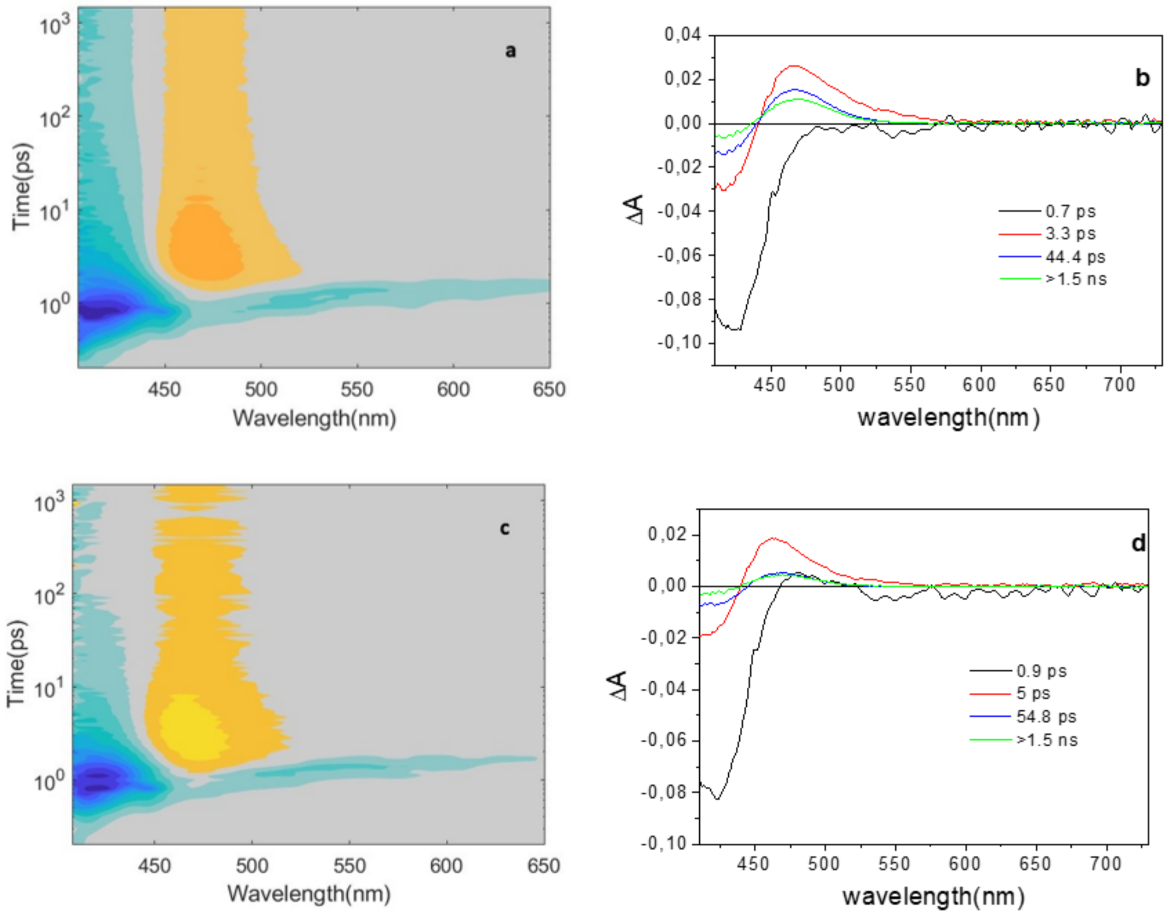

Figure S9: A) Time/wavelength map of the transient data recorded for compound **2** in MeOH upon excitation at 400 nm. B) EADS obtained from global analysis of the transient absorption data of compound **2** recorded in MeOH. C) Time/wavelength map of the transient data recorded for compound **1** in MeOH upon excitation at 400 nm. D) EADS obtained from global analysis of the transient absorption data of compound **1** recorded in MeOH.

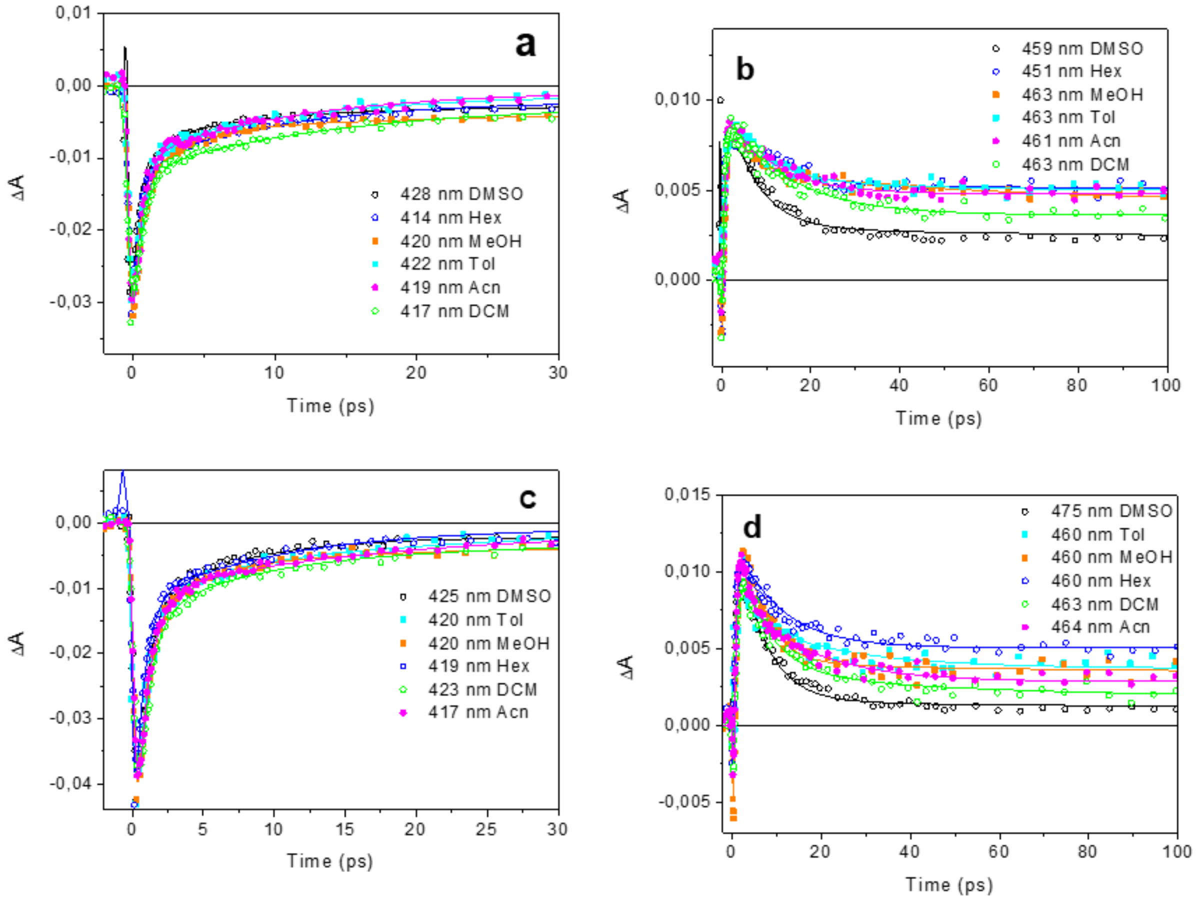

Figure S10: Comparison of kinetic traces recorded on the bleaching band (panels A) and C)) and on the product band (panel B) and D)) for compound **2** (panels A) and B)) and compound **1** (panels C) and D)) in different solvents.

Nanosecond transient absorption spectroscopy

Nanosecond transient absorption spectra were recorded with an in-house assembled setup. To generate the excitation wavelength at 425 nm, a tunable Nd:YAG-laser system (NT342B, Ekspla) consisting of the pump laser (NL300) with harmonics generators (SHG, THG) producing 355 nm light to pump an optical parametric oscillator (OPO) with SHG connected into a single device was used. The laser system was operated with a pulse length of 5 ns and a repetition rate of 10 Hz. The probe light running at 20 Hz was generated by a high-stability short arc xenon flash lamp (FX-1160, Excelitas Technologies) employing a modified PS302 controller (EG&G). The probe light was split equally into a signal and a reference beam by means of a 50/50 beam splitter and focused (bi-convex lens 75 mm) on the entrance slit of a spectrograph (SpectraPro-150, Princeton Instruments) with a grating of 150 $ln \cdot mm^{-1}$, blaze at 500 nm. The probe beam ($A = 1\ mm^2$) was set to pass through the sample cell and orthogonally overlapped with the excitation beam on a 1 mm × 1 cm area. The excitation energy was determined by recording the excitation power at the back of an empty sample holder. To correct for fluctuations in the spectral intensity of the flash lamp, the signal was normalized using the reference. The two beams were recorded at the same time using a gated intensified CCD camera (PI-MAX3, Princeton Instruments) with an adjustable gate of minimal 2.9 ns. Under standard settings a gate of 20 ns and software binning was used to improve the dynamic range and signal to noise ratio. Two delay generators (DG535 and DG645, Stanford Research Systems, Inc.) were used to trigger the excitation and to change the delay of the flash lamp together with the gate of the camera during the experiment. An in-house-written Labview program was used to control the setup.

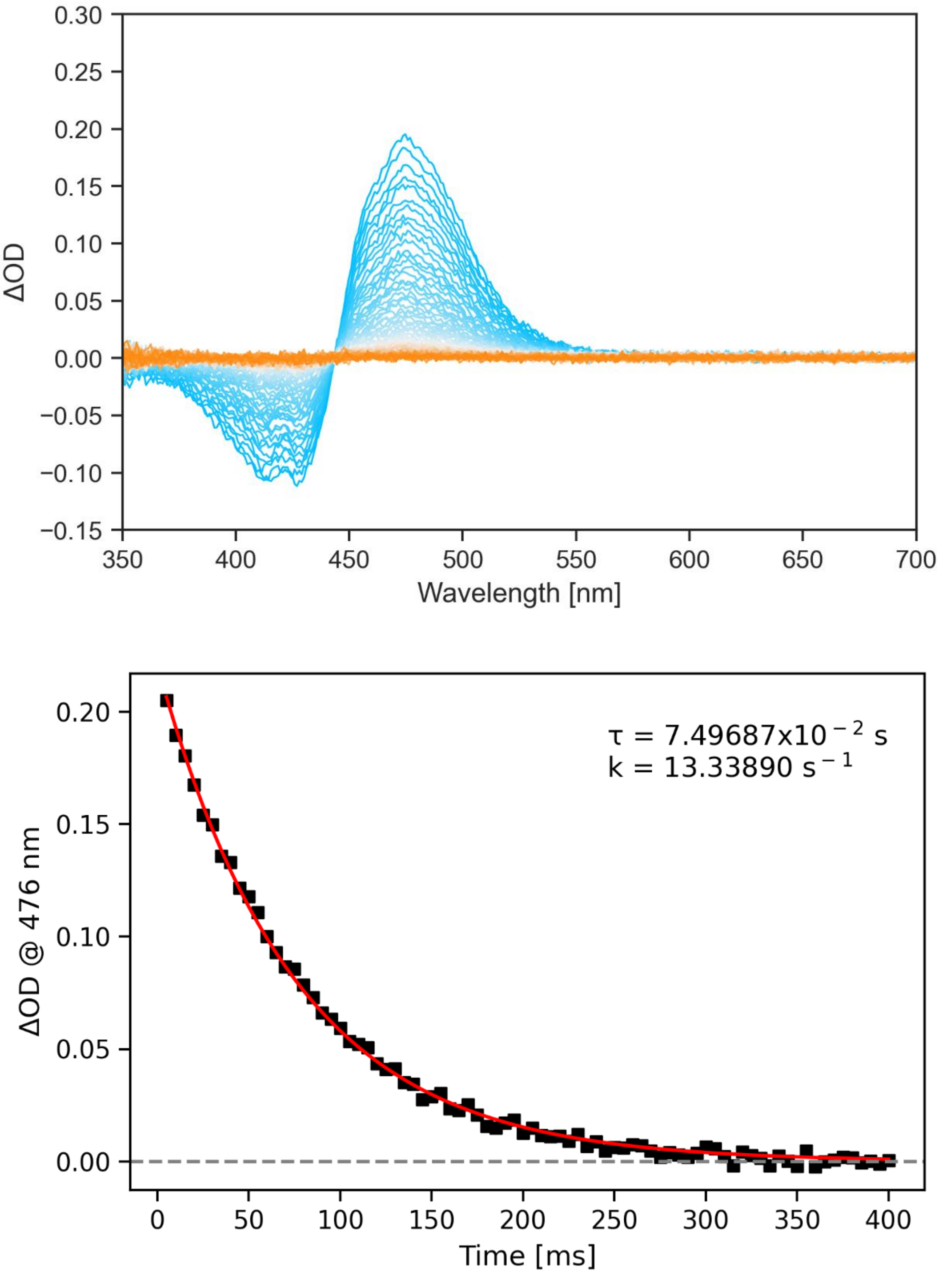

Figure S11: Nanosecond transient absorption of **1** in DCM at room temperature. The sample was irradiated at 425 nm (1.45 OD, 1.2 mJ) upon which the spectrum was recorded in steps of increasing delay. Bottom: Fit of the decay of the absorption maximum of the transient signal (red line) as obtained from a global analysis of the transient absorption spectra in the top panel.

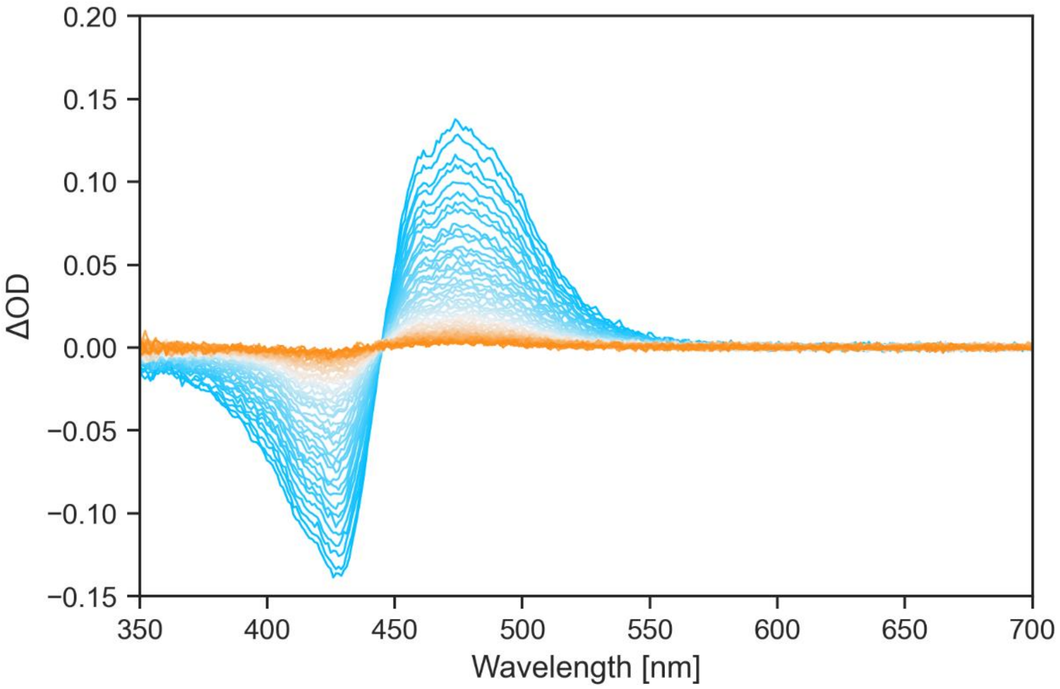

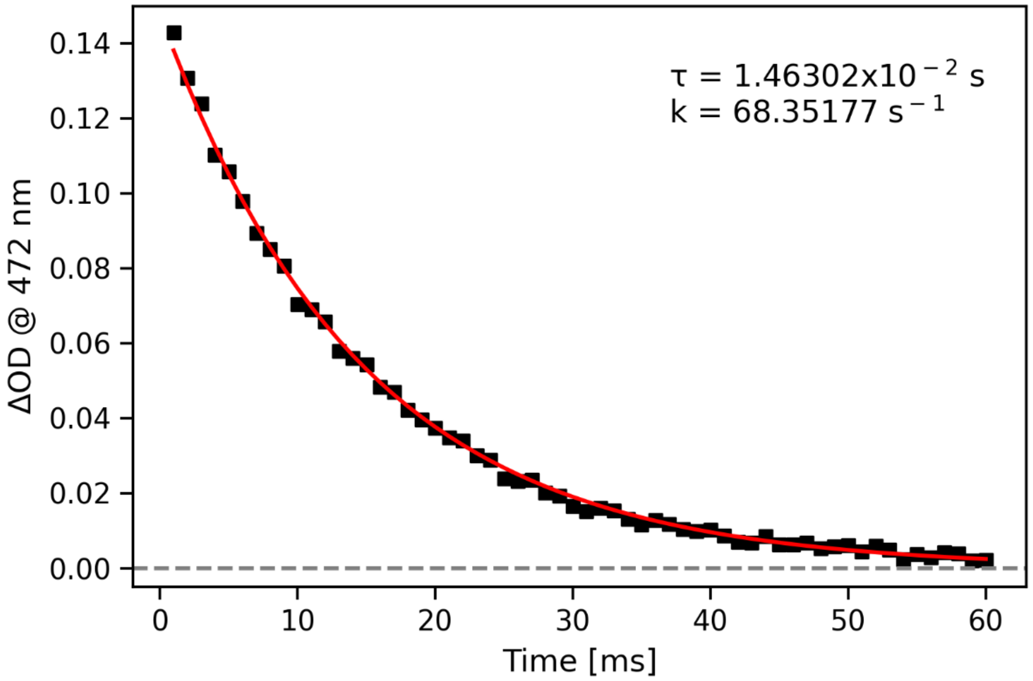

Figure S12: Nanosecond transient absorption of **1** in decanol at room temperature. The sample was irradiated at 425 nm (1.44 OD, 1.2 mJ) upon which the spectrum was recorded in steps of increasing delay. Bottom: Fit of the decay of the absorption maximum of the transient signal (red line) as obtained from a global analysis of the transient absorption spectra in the top panel.

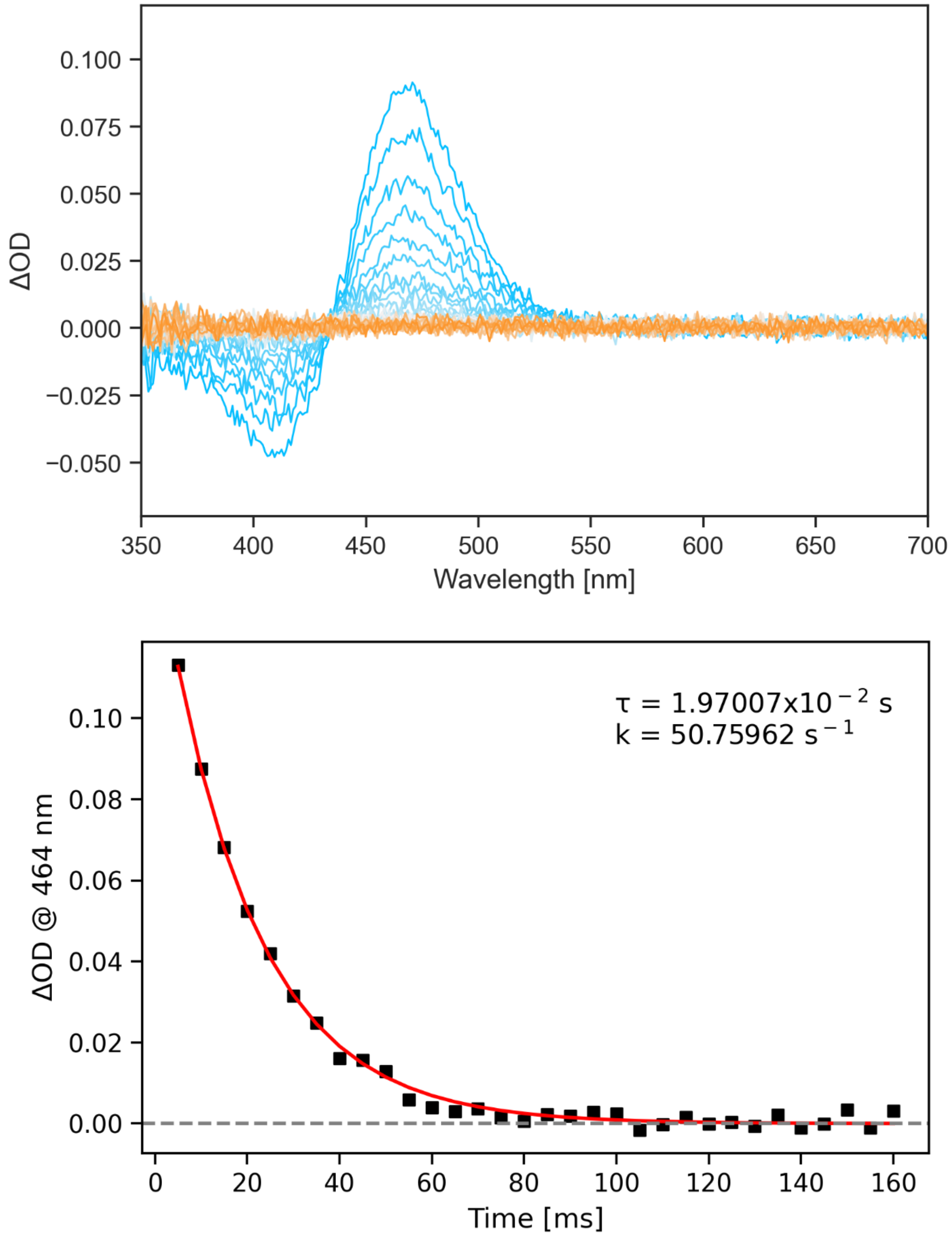

Figure S13: Nanosecond transient absorption of **1** in 1,4-dioxane at room temperature. The sample was irradiated at 425 nm (1.40 OD, 1.2 mJ) upon which the spectrum was recorded in steps of increasing delay. Bottom: Fit of the decay of the absorption maximum of the transient signal (red line) as obtained from a global analysis of the transient absorption spectra in the top panel.

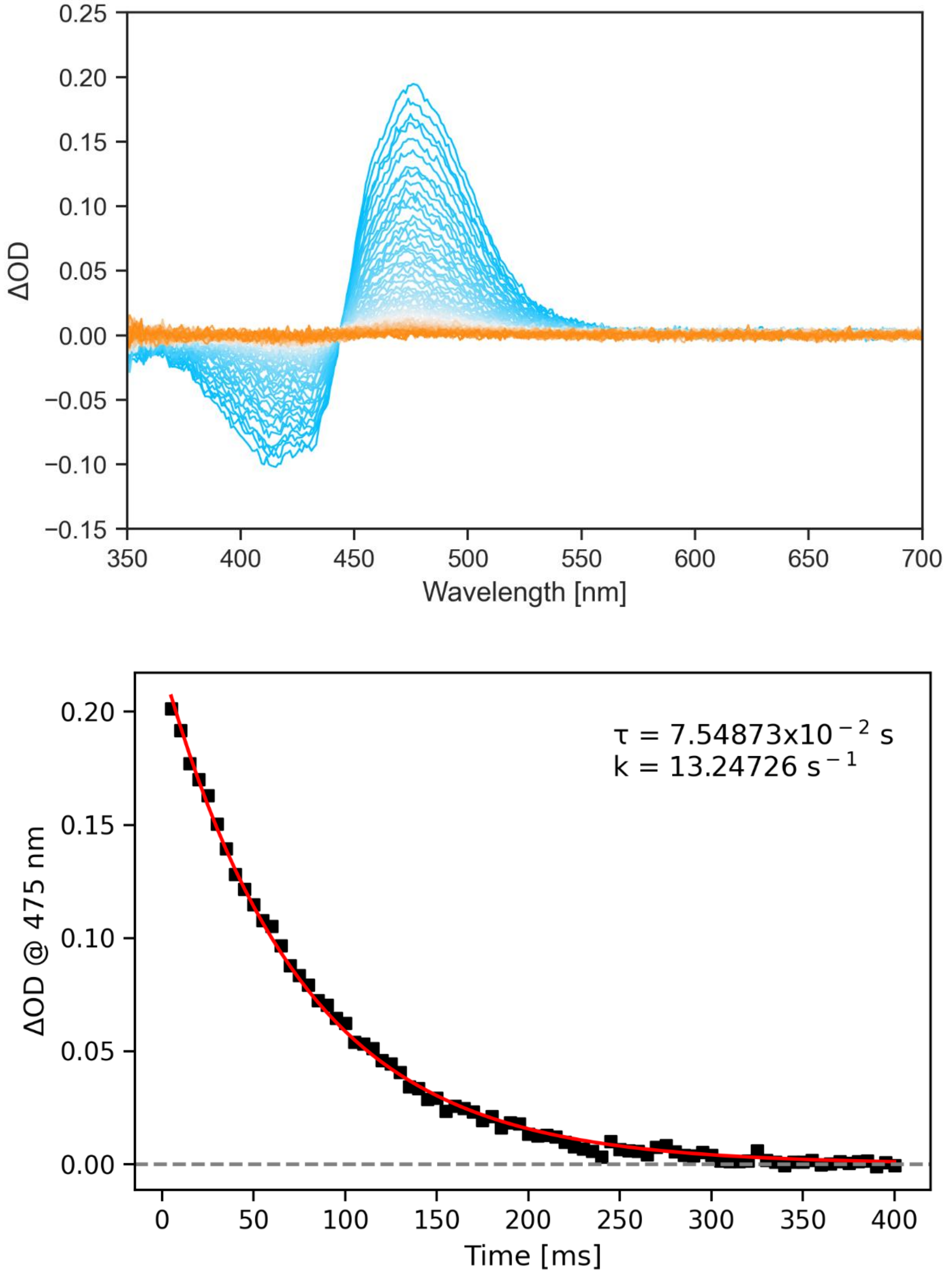

Figure S14: Nanosecond transient absorption of **1** in DMSO at room temperature. The sample was irradiated at 425 nm (1.12 OD, 1.2 mJ) upon which the spectrum was recorded in steps of increasing delay. Bottom: Fit of the decay of the absorption maximum of the transient signal (red line) as obtained from a global analysis of the transient absorption spectra in the top panel.

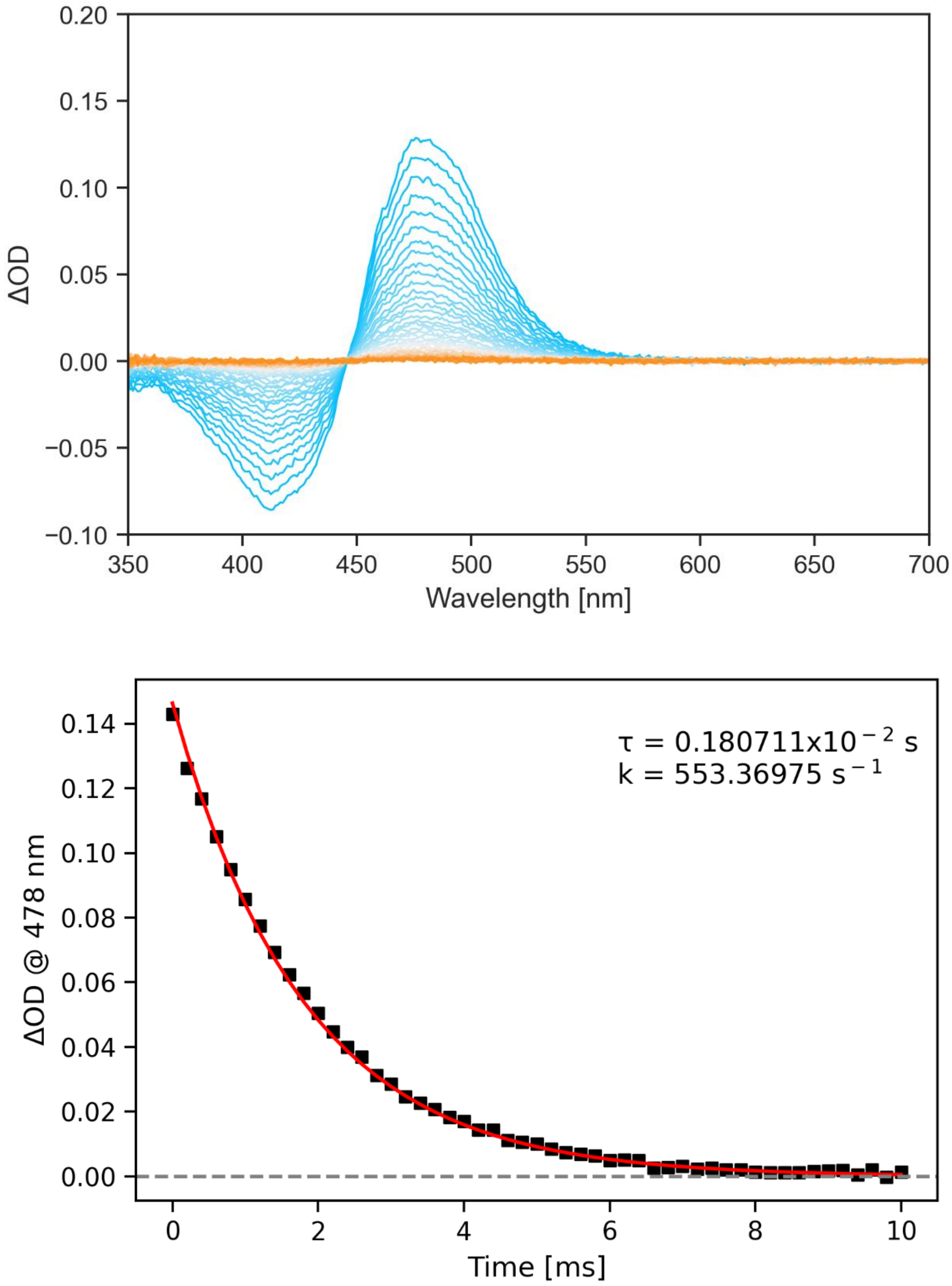

Figure S15: Nanosecond transient absorption of **1** in EtOH at room temperature. The sample was irradiated at 425 nm (1.30 OD, 1.2 mJ) upon which the spectrum was recorded in steps of increasing delay. Bottom: Fit of the decay of the absorption maximum of the transient signal (red line) as obtained from a global analysis of the transient absorption spectra in the top panel.

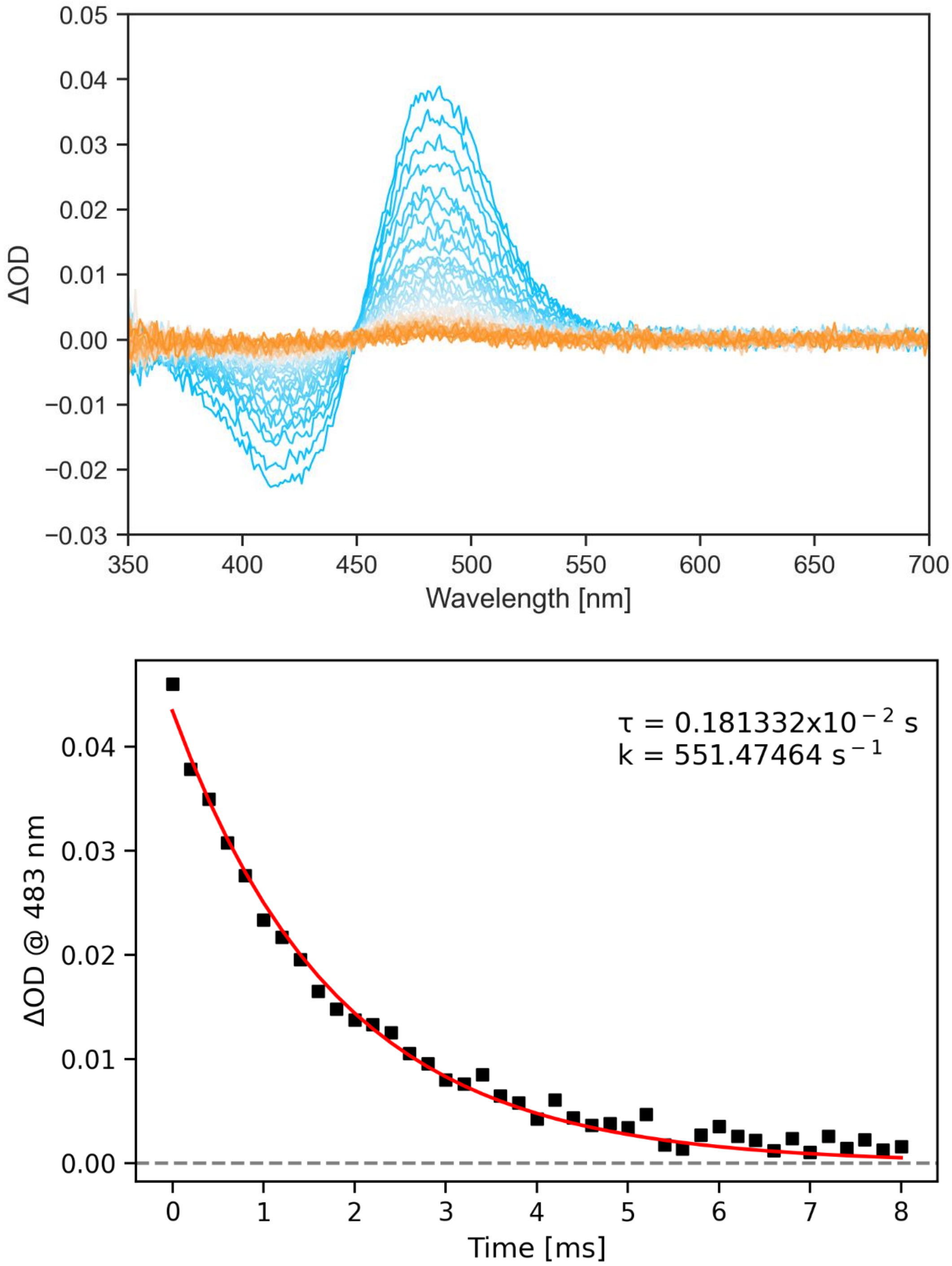

Figure S16: Nanosecond transient absorption of **1** in glycerol at room temperature. The sample was irradiated at 425 nm (1.25 OD, 1.2 mJ) upon which the spectrum was recorded in steps of increasing delay. Bottom: Fit of the decay of the absorption maximum of the transient signal (red line) as obtained from a global analysis of the transient absorption spectra in the top panel.

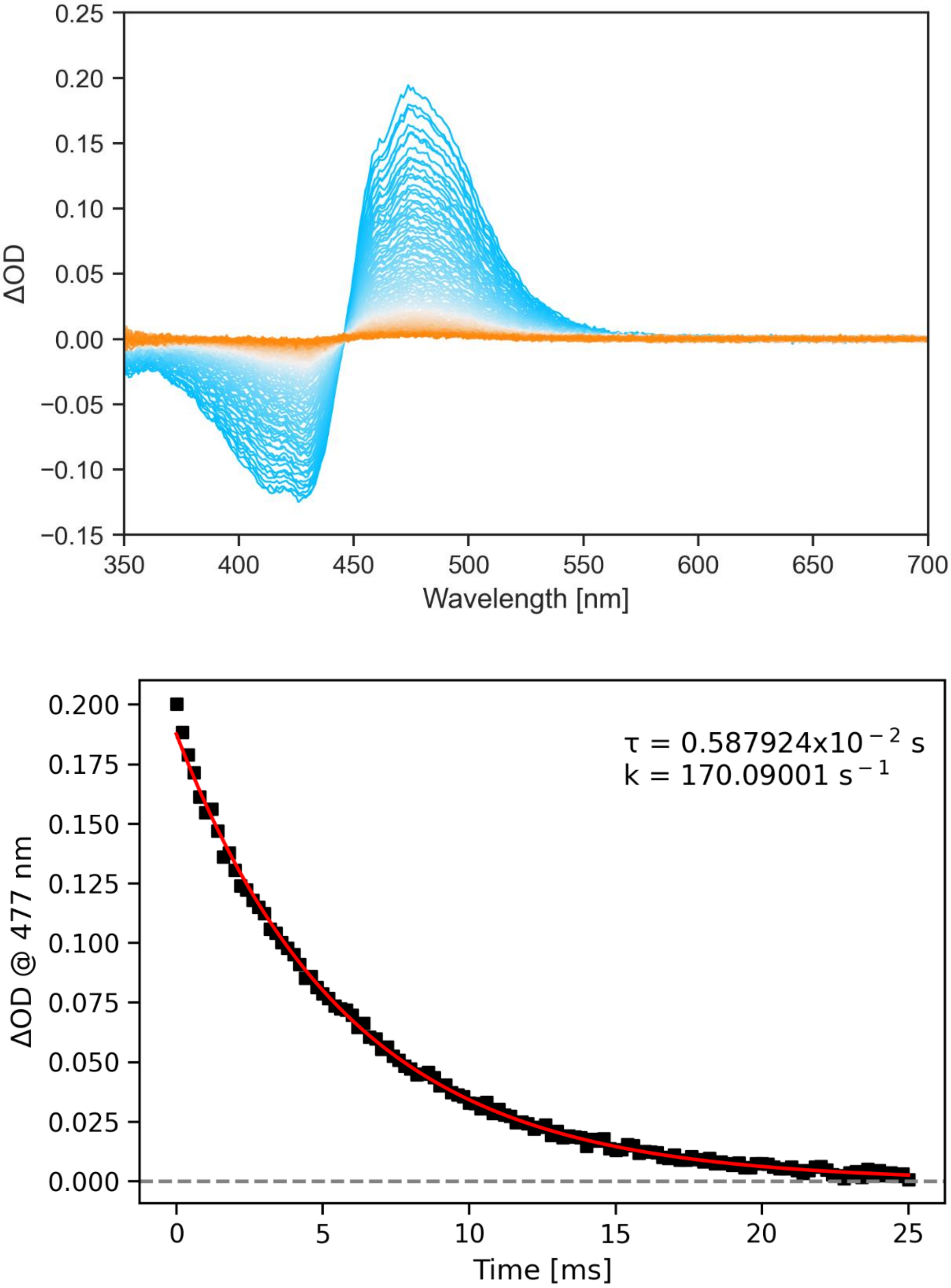

Figure S17: Nanosecond transient absorption of **1** in isopropanol at room temperature. The sample was irradiated at 425 nm (1.36 OD, 1.2 mJ) upon which the spectrum was recorded in steps of increasing delay. Bottom: Fit of the decay of the absorption maximum of the transient signal (red line) as obtained from a global analysis of the transient absorption spectra in the top panel.

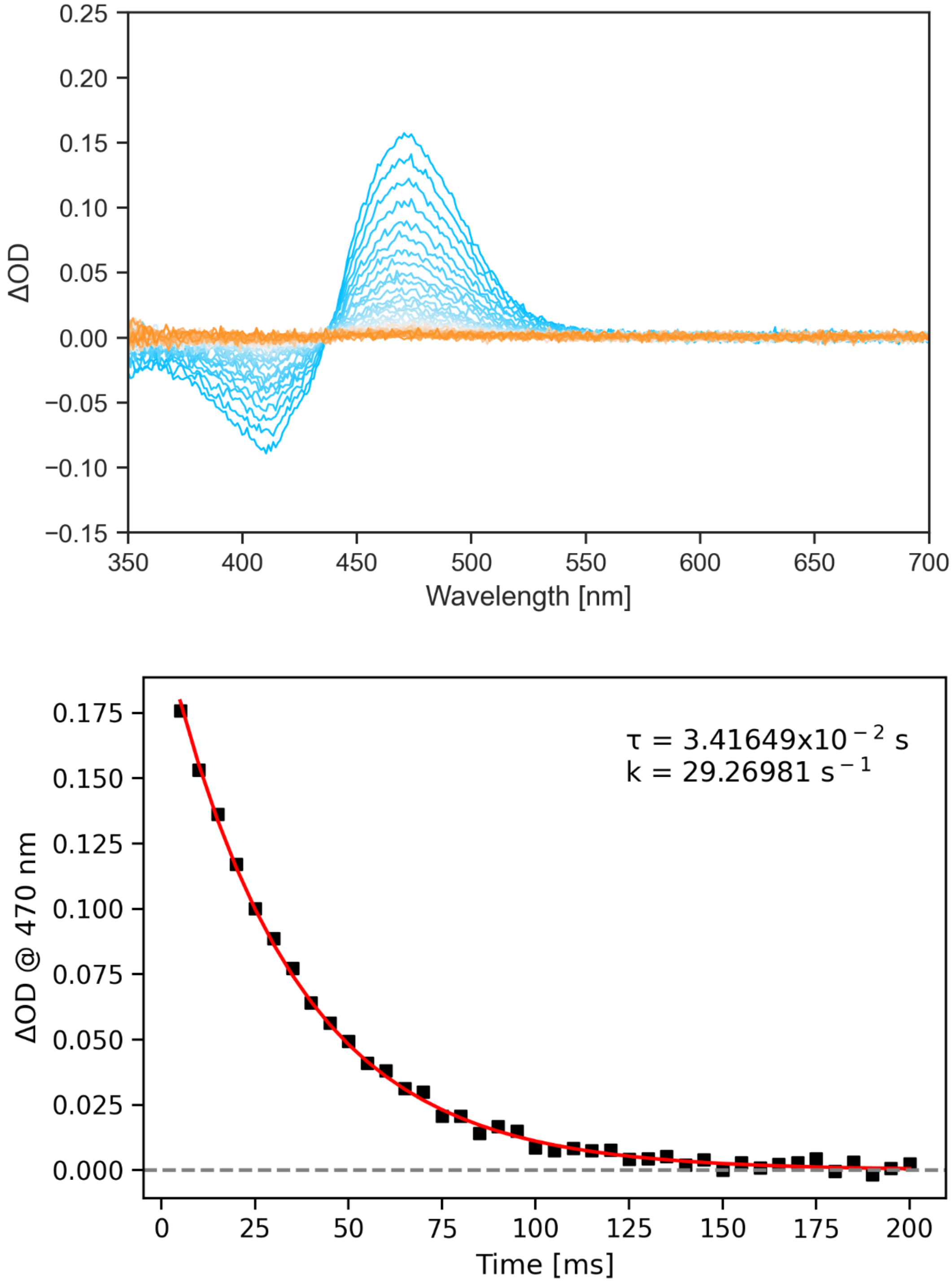

Figure S18: Nanosecond transient absorption of **1** in MeCN at room temperature. The sample was irradiated at 425 nm (1.36 OD, 1.2 mJ) upon which the spectrum was recorded in steps of increasing delay. Bottom: Fit of the decay of the absorption maximum of the transient signal (red line) as obtained from a global analysis of the transient absorption spectra in the top panel.

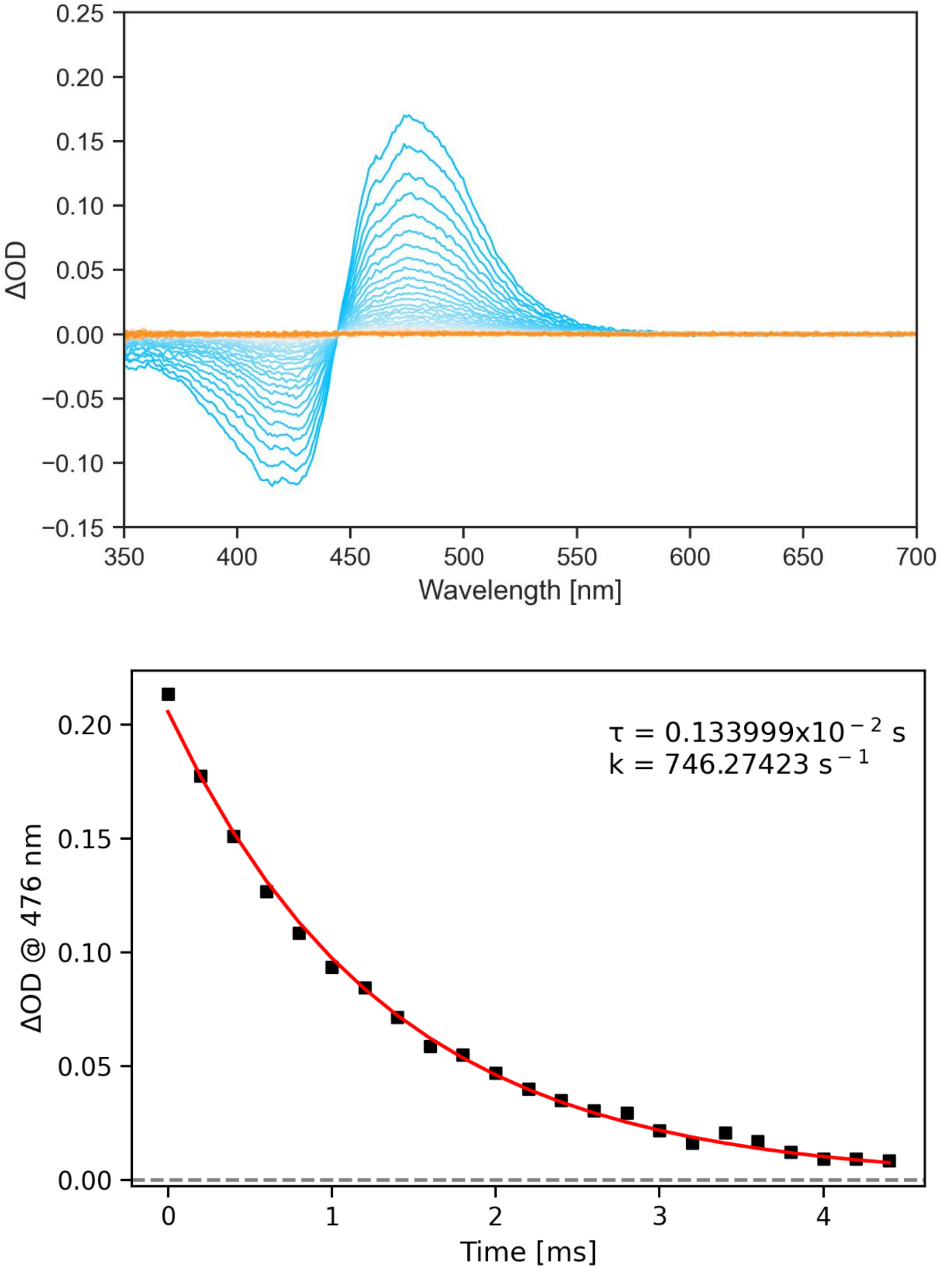

Figure S19: Nanosecond transient absorption of **1** in MeOH at room temperature. The sample was irradiated at 425 nm (1.60 OD, 1.2 mJ) upon which the spectrum was recorded in steps of increasing delay. Bottom: Fit of the decay of the absorption maximum of the transient signal (red line) as obtained from a global analysis of the transient absorption spectra in the top panel.

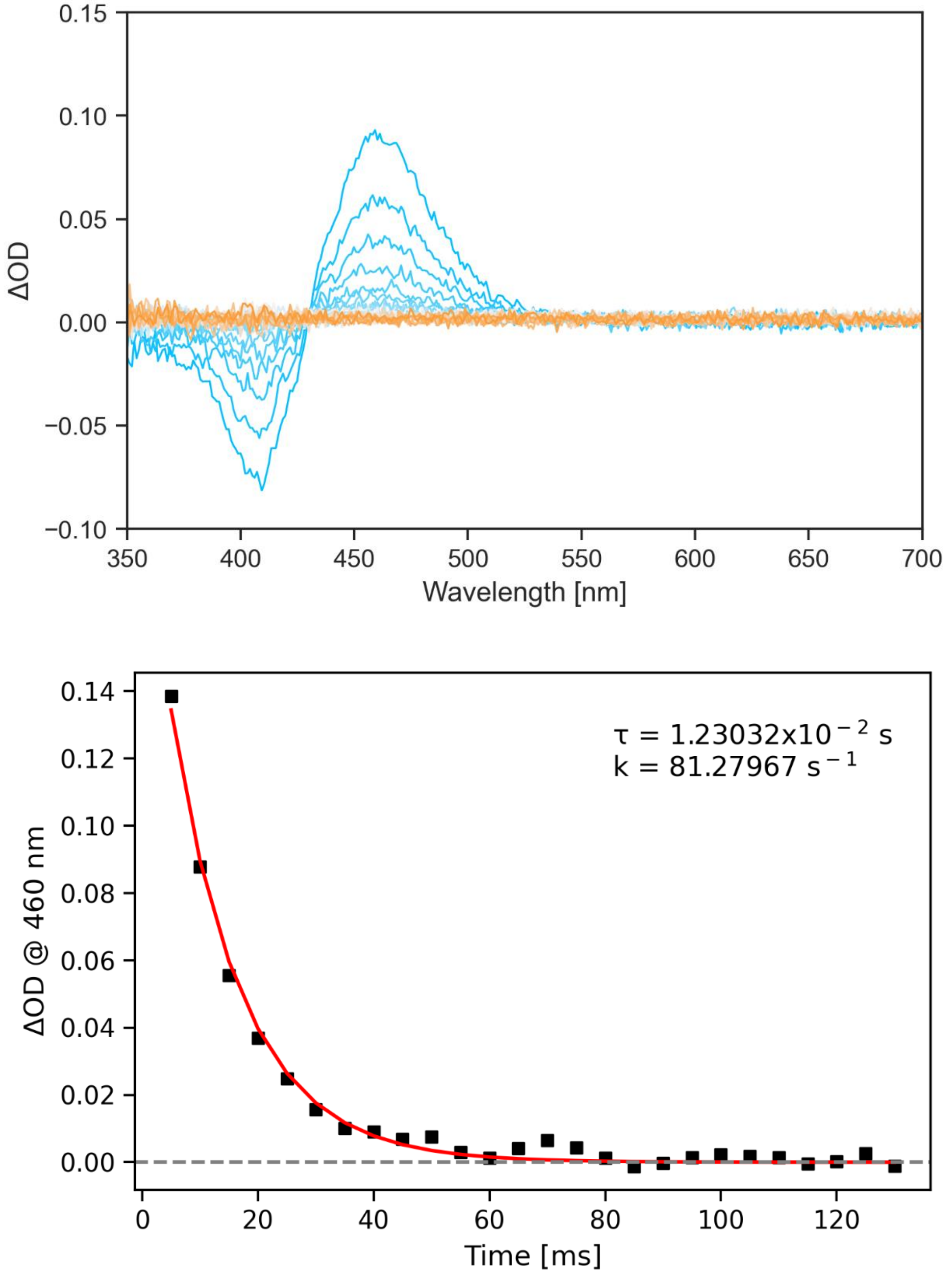

Figure S20: Nanosecond transient absorption of **1** in pentane at room temperature. The sample was irradiated at 425 nm (1.38 OD, 1.2 mJ) upon which the spectrum was recorded in steps of increasing delay. Bottom: Fit of the decay of the absorption maximum of the transient signal (red line) as obtained from a global analysis of the transient absorption spectra in the top panel.

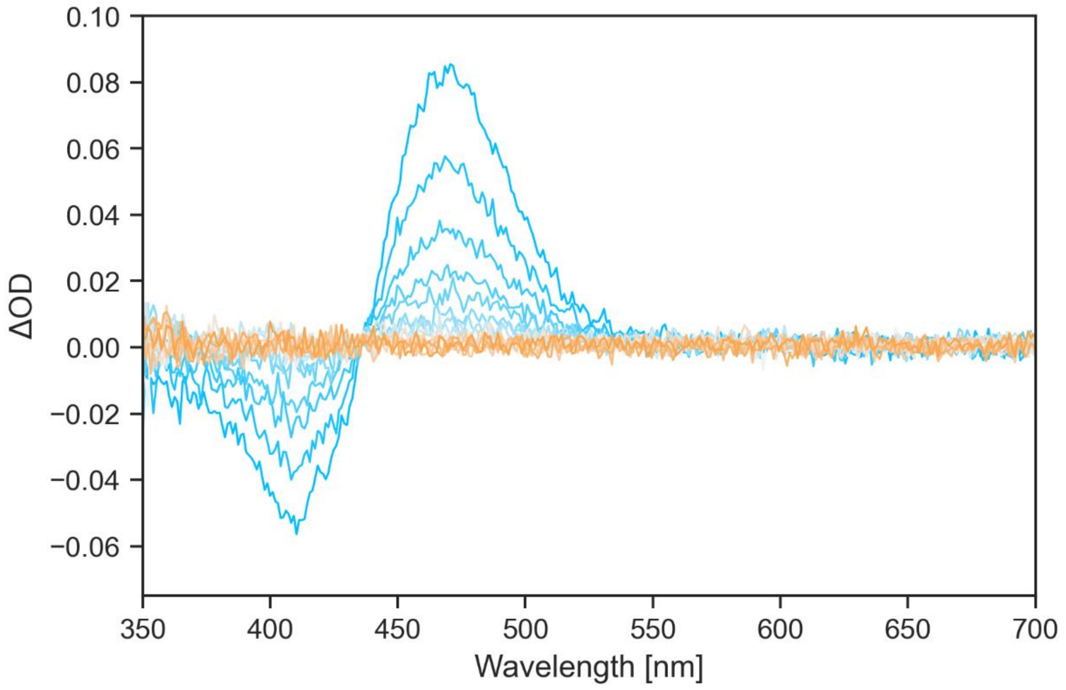

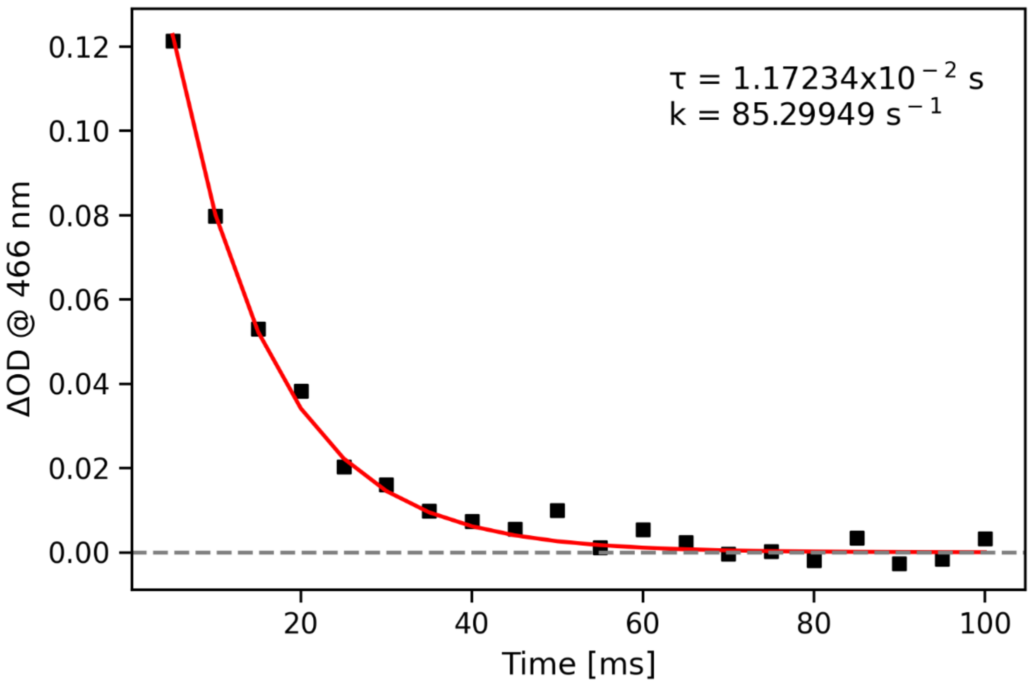

Figure S21: Nanosecond transient absorption of **1** in THF at room temperature. The sample was irradiated at 425 nm (1.40 OD, 1.2 mJ) upon which the spectrum was recorded in steps of increasing delay. Bottom: Fit of the decay of the absorption maximum of the transient signal (red line) as obtained from a global analysis of the transient absorption spectra in the top panel.

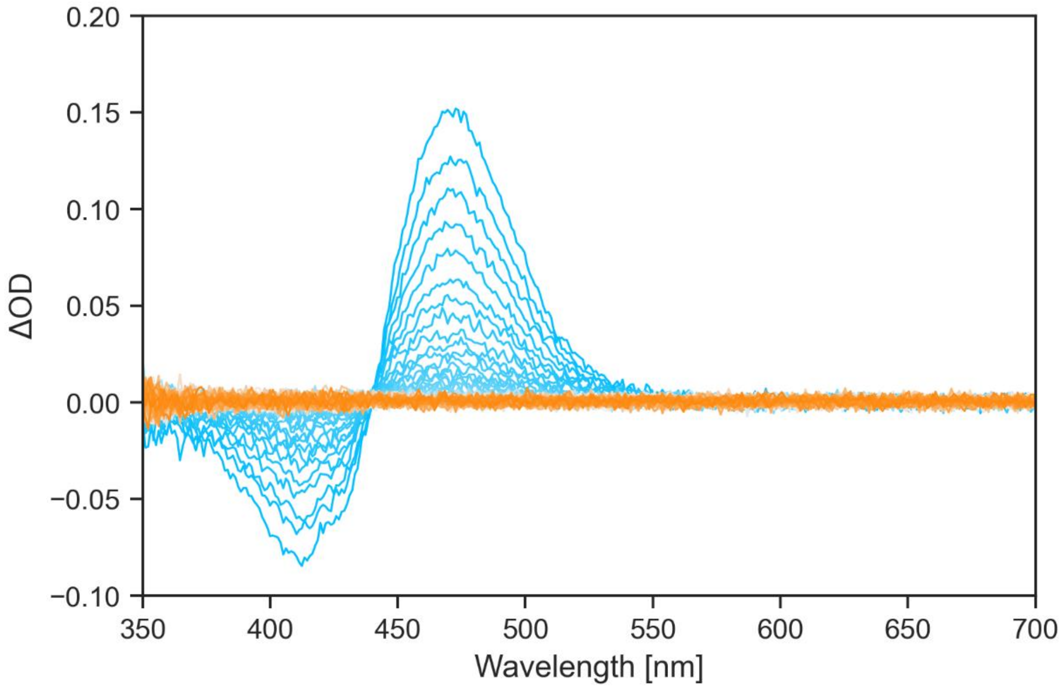

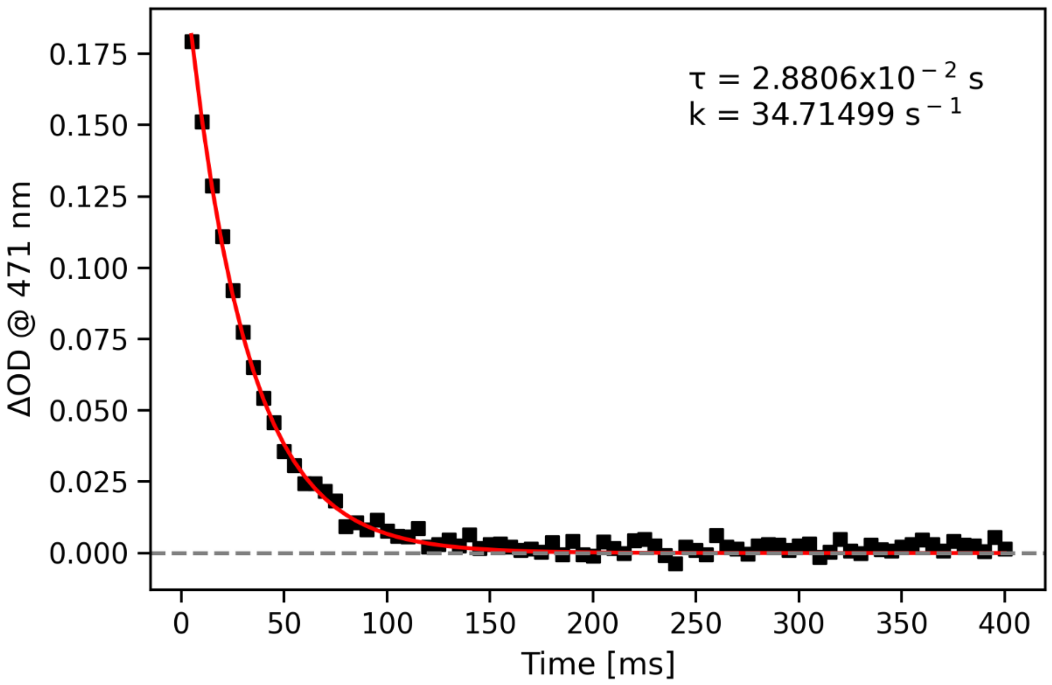

Figure S22: Nanosecond transient absorption of **1** in toluene at room temperature. The sample was irradiated at 425 nm (1.47 OD, 1.2 mJ) upon which the spectrum was recorded in steps of increasing delay. Bottom: Fit of the decay of the absorption maximum of the transient signal (red line) as obtained from a global analysis of the transient absorption spectra in the top panel.

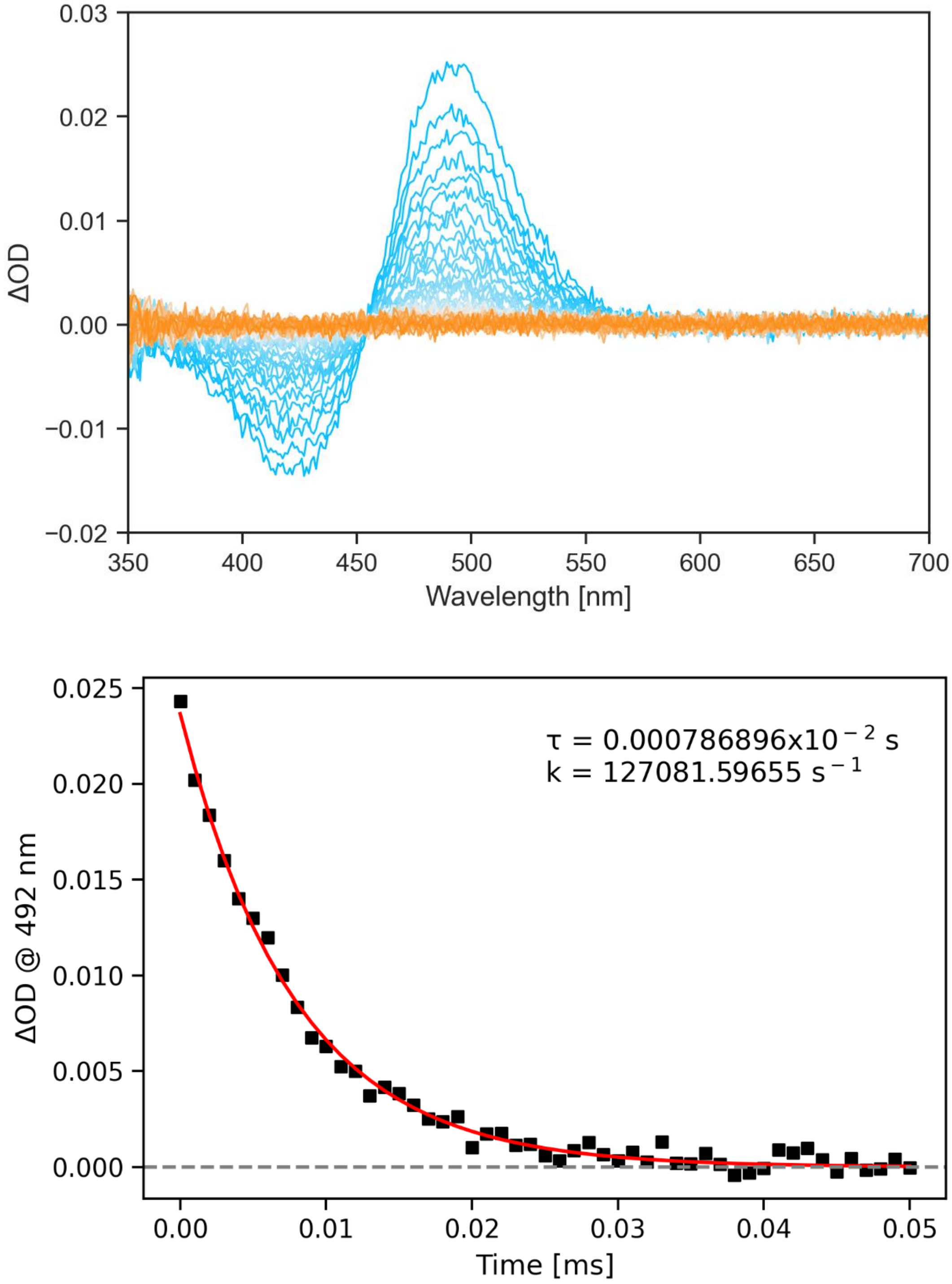

Figure S23: Nanosecond transient absorption of **1** in water at room temperature. The sample was irradiated at 425 nm (1.30 OD, 1.2 mJ) upon which the spectrum was recorded in steps of increasing delay. Bottom: Fit of the decay of the absorption maximum of the transient signal (red line) as obtained from a global analysis of the transient absorption spectra in the top panel.

7. Computational details

For the analysis of the processes at the ground state, the geometries were optimized at the r^2SCAN-3c level of theory[7], using no solvent or water in the implicit CPCM solvent method as implemented in the ORCA 5.0.3 software.[8] The nature of the stationary point found was confirmed computing the hessian matrix and inspecting the number of imaginary frequencies found (0 for the minima, 1 for the transition states). The nature of the *TEZI* transition state was checked with a broken-symmetry calculation and by performing stability tests of the optimized solution. In all cases, a closed-shell species was found. Single point corrections were run at the DSD-BLYP/def2-QZVP[9,10] level of theory, taking into account the implicit solvent (water, CPCM) when necessary.

Semiempirical calculations and nonadiabatic molecular dynamics simulations were conducted at the OM2/MRCI level of theory,[11,12] as implemented in the MNDO program.[13] The active space in the MRCI calculations included twelve electrons in twelve orbitals (12,12). All the orbitals were of π and n character. An antibonding orbital mostly localized on the MeO oxygen was chosen to stabilise the active space (see Figure S26).

For the MRCI treatment, three configuration state functions were chosen as references, namely the leading configuration with two singly occupied orbitals (which defines the ROHF formalism) and the two closed-shell configurations derived therefrom (i.e., the singlet configurations with doubly occupied HOMO or LUMO of the closed-shell ground state). The MRCI wavefunction was built by allowing all single and double excitations from these three references (CISD).

All the optimizations were conducted including the lowest three singlet states. The geometry optimization of minima employed the BFGS update. The nature of the minima and transition states was checked inspecting the number of imaginary frequencies after the calculation of the force constants at the optimized geometry (i.e., 0 for minima, 1 for transition states). Transition states were located using the eigenvector following algorithm, while the default optimizer was employed for the minima. The conical intersection was optimized using a modified version[14] of the Lagrange-Newton algorithm proposed by Manaa and Yarkony.[15]

Nonadiabatic molecular dynamics (NAMD) simulations on the E geometry were performed using the Tully surface-hopping (TSH) method as implemented in the MNDO program, with an analytical evaluation of the nonadiabatic coupling vectors.[16] All simulations were in a canonical ensemble maintained by a Nosé-Hoover thermostat (T=300 K). The sampling of the initial structures and relative initial velocities was obtained via a preliminary Wigner sampling on the geometry optimised at the r^2SCAN-3c level. The two lowest singlet states were included

in the NAMD runs. The initial state chosen for starting the dynamics was S1. 400 runs of 2000 fs with time step of 0.1 fs were evaluated. The default empirical decoherence correction of 0.1 Hartree was used.[17]

All xyz coordinates for all the compounds considered are provided as a separate additional file in a figshare repository with the following DOI: 10.6084/m9.figshare.23276999.

A video of a productive E-Z NAMD trajectory computed at the OM2/MRCI level is provided as a separate file.

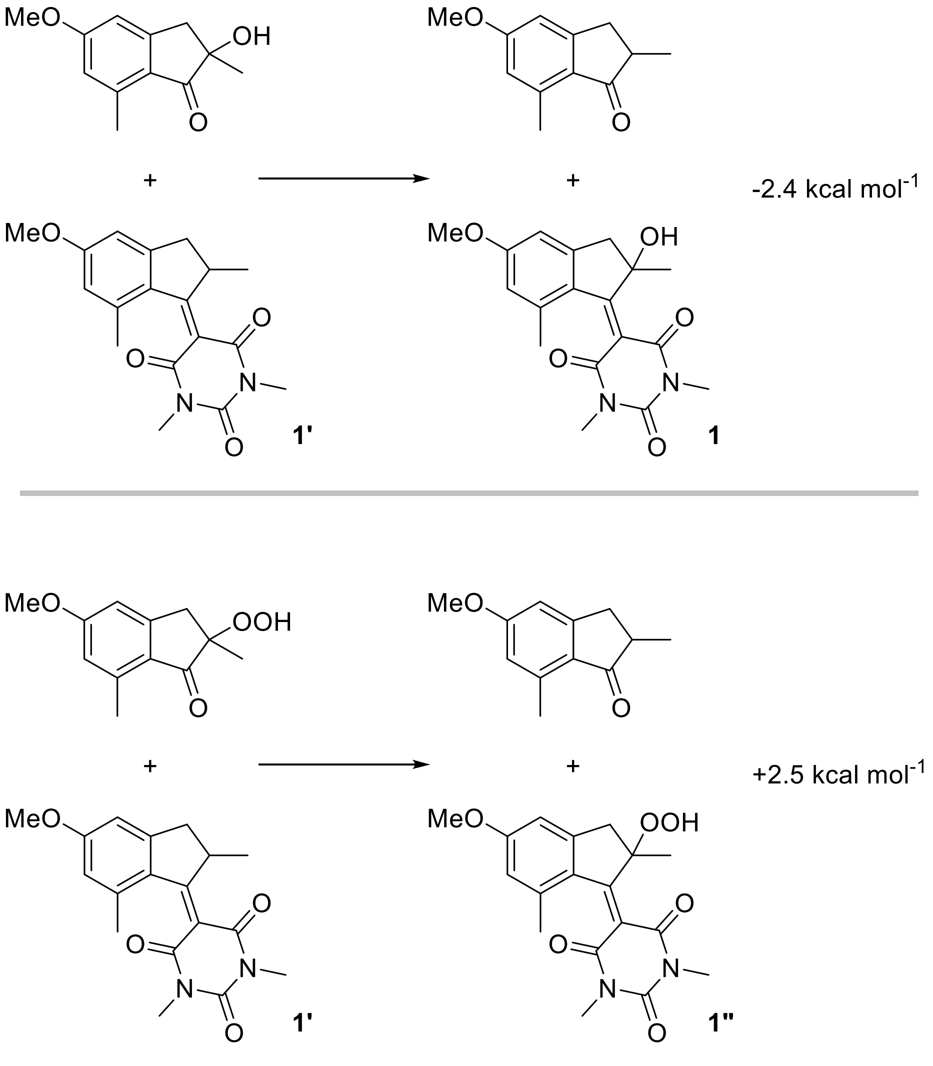

Figure S24: Isodesmic reactions used to evaluate the relative stability of the hydroxylated (**1**) and peroxy (**1''**) motors versus **1'** evaluated at the DSD-BLYP/def2-QZVP//r^{2}SCAN-3c (gas phase). The product **1** is considerably more stable than **1'** and of the intermediate **1''**. It is possible that additional solvation stabilize the peroxy intermediate that was experimentally observed (see Figure S1).

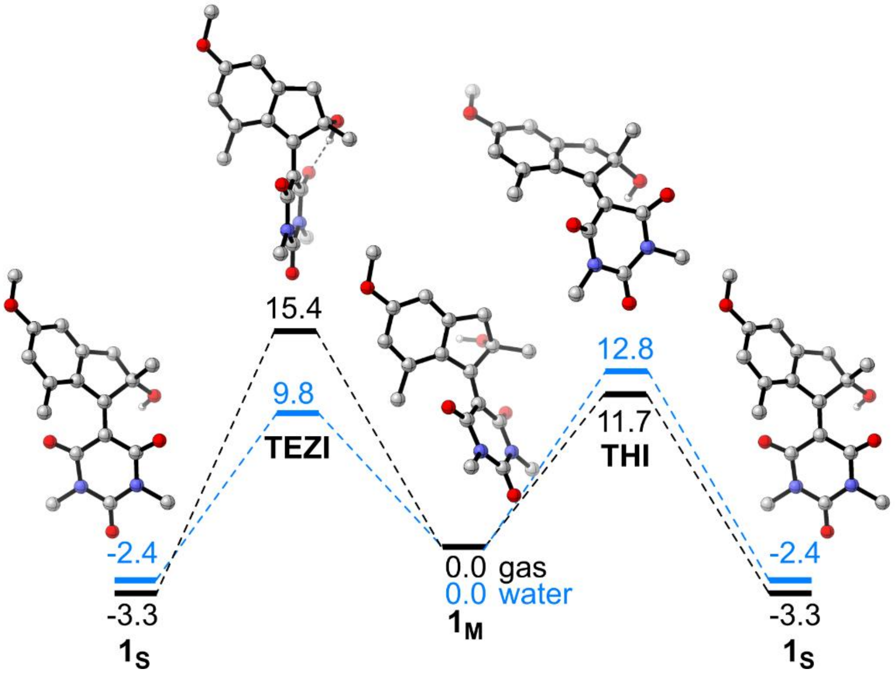

Figure S25: Gibbs free energies (DSD-BLYP-D3BJ/def2-QZVP//r2SCAN-3c level, in kcal mol^{-1}) for the thermal processes of motor **1** on the ground state surface, when the intramolecular H bond is not present. The black surface depicts the energies of the structures computed in the gas phase. The blue surface, in contrast, represents the energies of the structures computed using water as implicit solvent (CPCM). The results are qualitatively similar to the ones obtained in the presence of the H bond, but with overall lower thermal barriers.

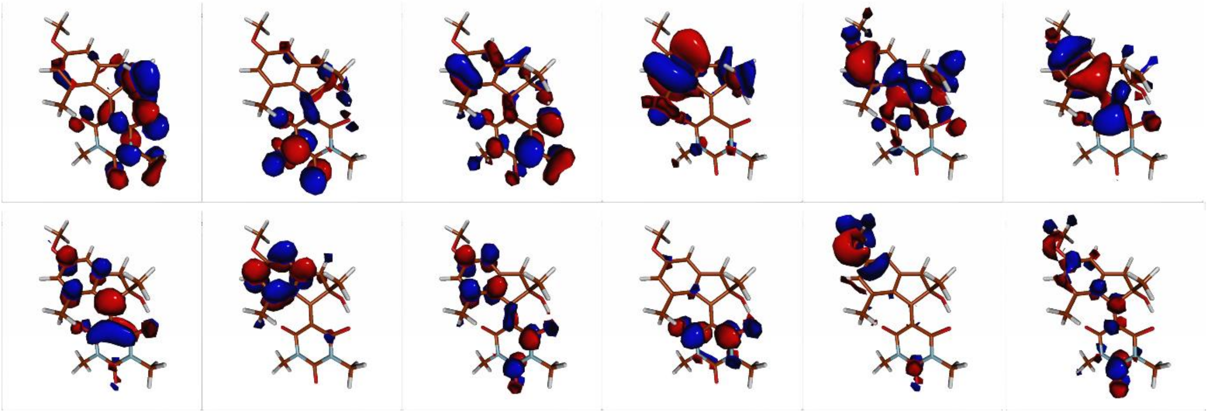

Figure S26: Active space orbitals employed in the OM2/MRCI calculations.

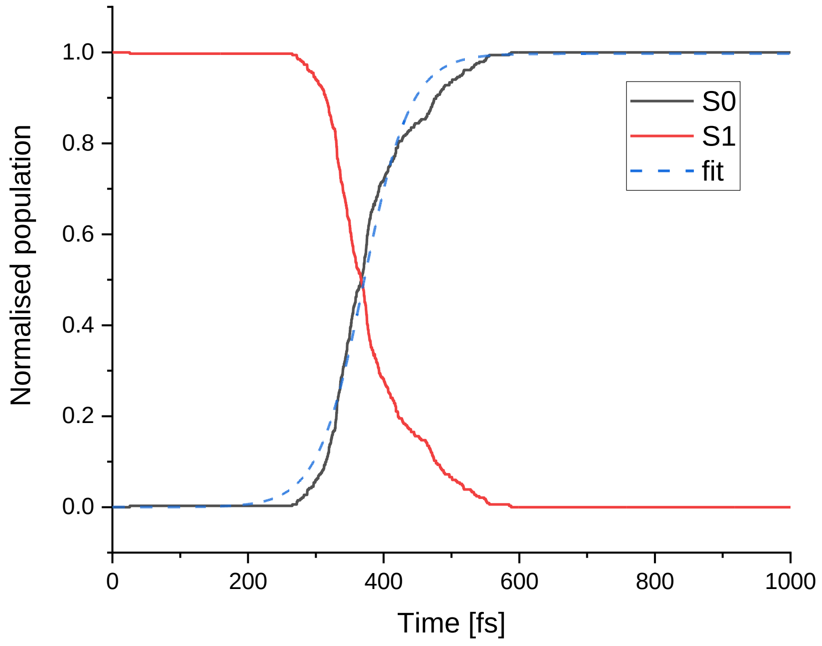

Figure S27: Averaged S0 and S1 states population as a function of time (for the first ps; the population does not change afterwards). The fit to extract the halflife (t) was obtained minimizing the function y=A/(1+exp(-B*(x-t))). The value obtained was a halflife of 371 fs and a lifetime of 536 fs.

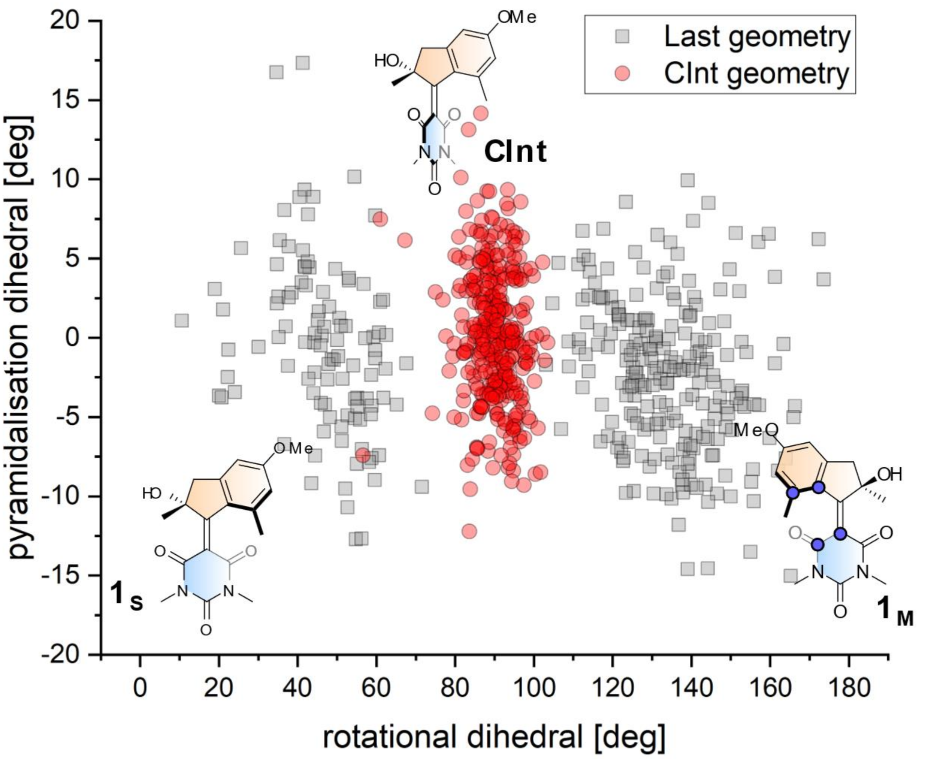

Figure S28: Values of selected dihedral angle values defining the geometries of the hopping points (in red) and evolution of each trajectory at the time step of 2000 fs (in grey, the ones with a dihedral angle of ca. 50° are geometries corresponding to **1**$_S$, the ones at 140° are **1**$_M$). The rotational dihedral angle is defined using the atoms highlighted in **1**$_M$ in the figure. The carbon atom used to define the pyramidalization is the quaternary carbon of the C=C on the barbiturate moiety side. The hopping points (CInt) are mainly distributed around a region defining the so-called "rotation" movement in the excited state with a 90° dihedral angle formally breaking the C=C bond. The estimated quantum yield for the EZ isomerization (68%) is obtained by dividing the total number of trajectories that populated the **1**$_M$ by the total number of trajectories. It can be noted that the pyramidalization at Cint is minimal, a property typical of excited states for motor characterised by a high degree of charge transfer.

8. References

1 P. A. Delaney, R. A. W. Johnstone and I. D. Entwistle, *J. Chem. Soc. Perkin Trans. 1*, 1986, 1855.

2 *Bruker, APEX3 (V2019.1-0), SAINT (Version 8.40A) SADABS (Version 2016/1). Bruker AXS Inc., Madison, Wisconsin, USA.*

3 L. Krause, R. Herbst-Irmer, G. M. Sheldrick and D. Stalke, *J. Appl. Crystallogr.*, 2015, **48**, 3–10.

4 G. M. Sheldrick and IUCr, *Acta Crystallogr. Sect. A Found. Adv.*, 2015, **71**, 3–8.

5 G. M. Sheldrick, *Acta Crystallogr Sect. A*, 2008, **A64**, 112–122.

6 J. J. Snellenburg, S. Laptenok, R. Seger, K. M. Mullen and I. H. M. van Stokkum, *J. Stat. Softw.*, 2012, **49**, 1–22.

7 S. Grimme, A. Hansen, S. Ehlert and J. M. Mewes, *J. Chem. Phys.*, 2021, **154**, 064103.

8 F. Neese, F. Wennmohs, U. Becker and C. Riplinger, *J. Chem. Phys.*, 2020, **152**, 224108.

9 F. Weigend and R. Ahlrichs, *Phys. Chem. Chem. Phys.*, 2005, **7**, 3297–3305.

10 S. Kozuch, D. Gruzman and J. M. L. Martin, *J. Phys. Chem. C*, 2010, **114**, 20801–20808.

11 W. Weber and W. Thiel, *Theor. Chem. Acc.*, 2000, **103**, 495–506.

12 W. Thiel, *Wiley Interdiscip. Rev. Comput. Mol. Sci.*, 2014, **4**, 145–157.

13 W. Thiel, *MNDO Ver. 7.0*.

14 T. W. Keal, A. Koslowski and W. Thiel, *Theor. Chem. Acc.*, 2007, **118**, 837–844.

15 M. R. Manaa and D. R. Yarkony, *J. Chem. Phys.*, 1993, **99**, 5251–5256.

16 E. Fabiano, T. W. Keal and W. Thiel, *Chem. Phys.*, 2008, **349**, 334–347.

17 G. Granucci, M. Persico and A. Zoccante, *J. Chem. Phys.*, 2010, **133**, 134111.

9. Appendix

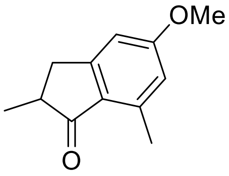

S1

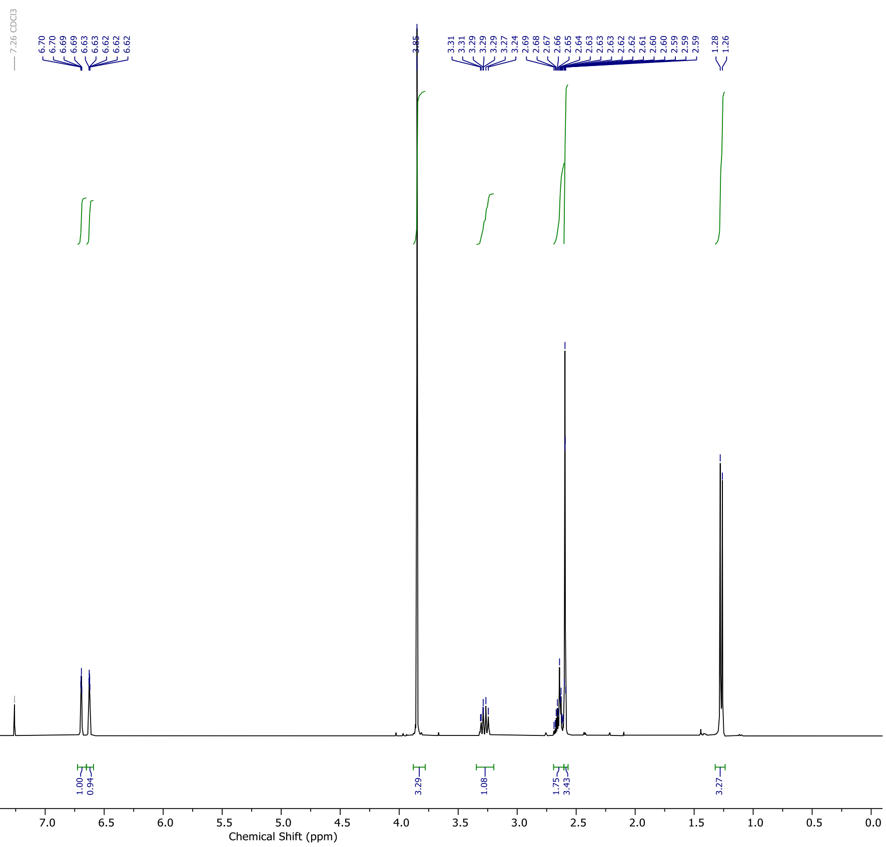

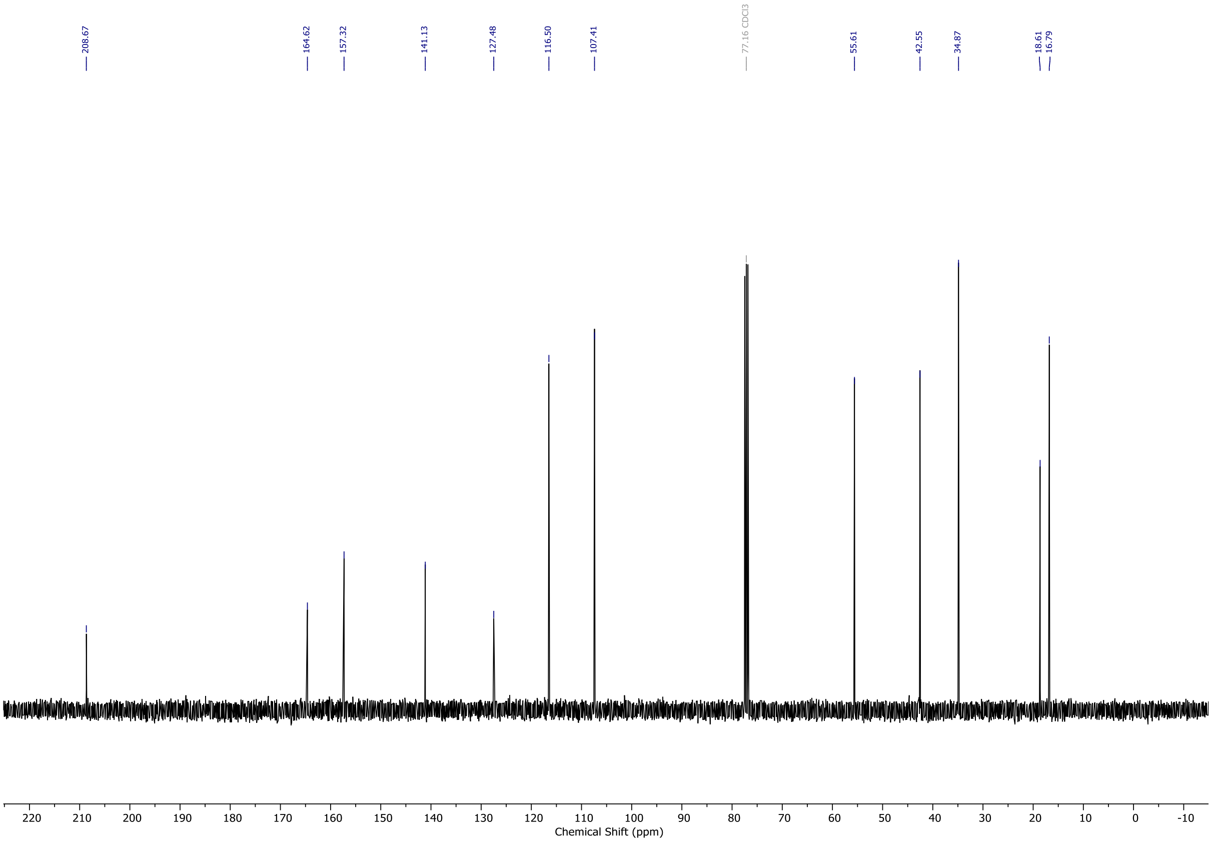

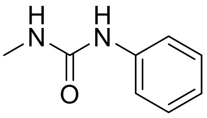

S2

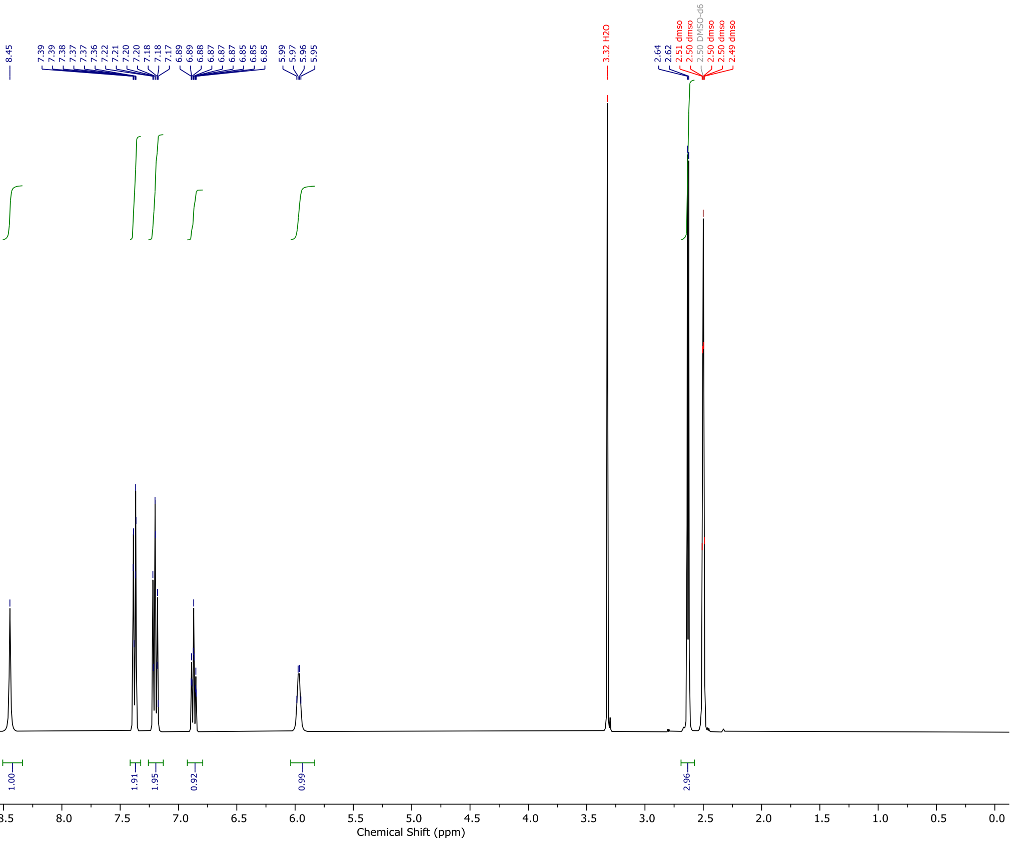
8.45
7.39 7.39 7.38 7.37 7.37 7.36 7.22 7.21 7.20 7.20 7.18 7.18 7.17
6.89 6.89 6.88 6.87 6.87 6.85 6.85 6.85
5.99 5.97 5.96 5.95
3.32 H2O
2.64 2.62 2.51 dmso 2.50 dmso 2.50 DMSO-d6 2.50 dmso 2.50 dmso 2.49 dmso
1.00 1.91 1.95 0.92 0.99 2.96
Chemical Shift (ppm)

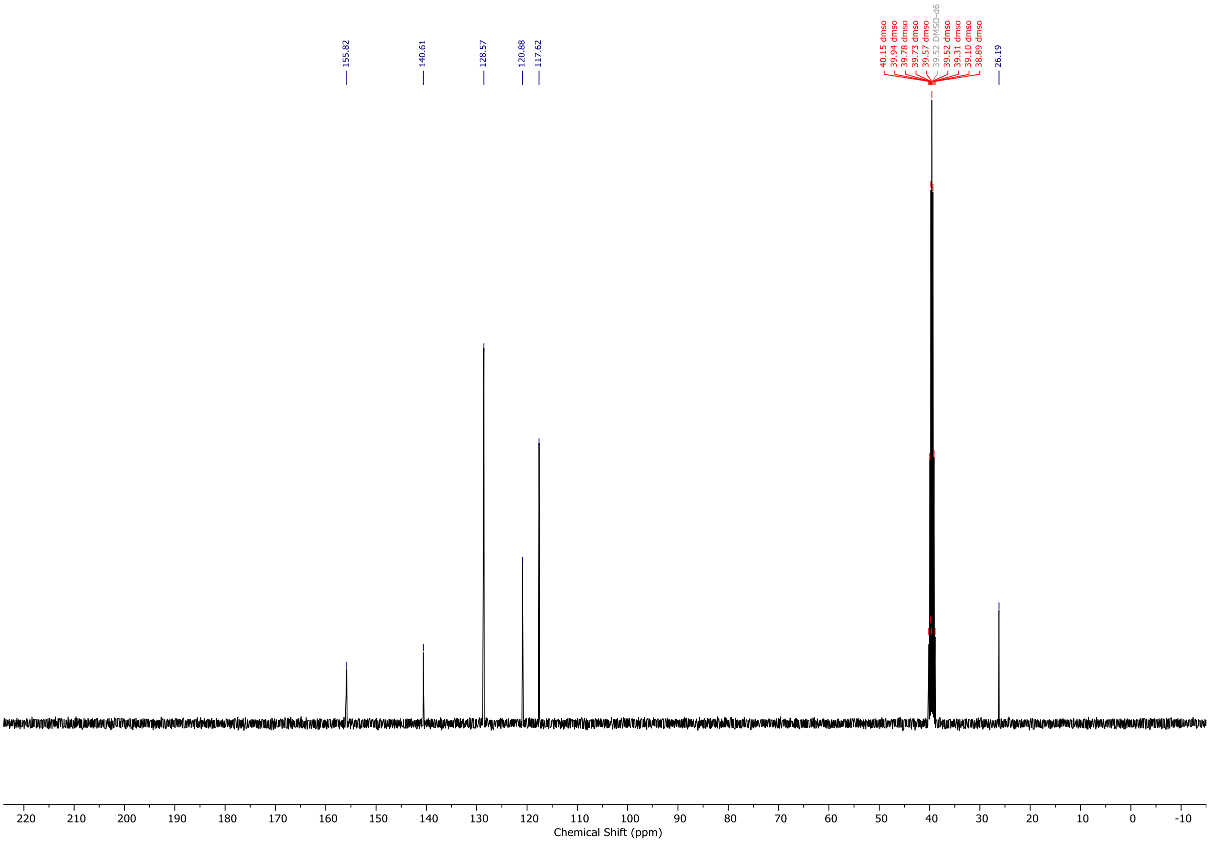
155.82
140.61
128.57
120.88
117.62
40.15 dmso 39.94 dmso 39.78 dmso 39.73 dmso 39.57 dmso 39.52 DMSO-d6 39.52 dmso 39.31 dmso 39.10 dmso 38.89 dmso
26.19
Chemical Shift (ppm)

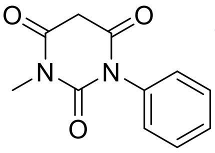

S3

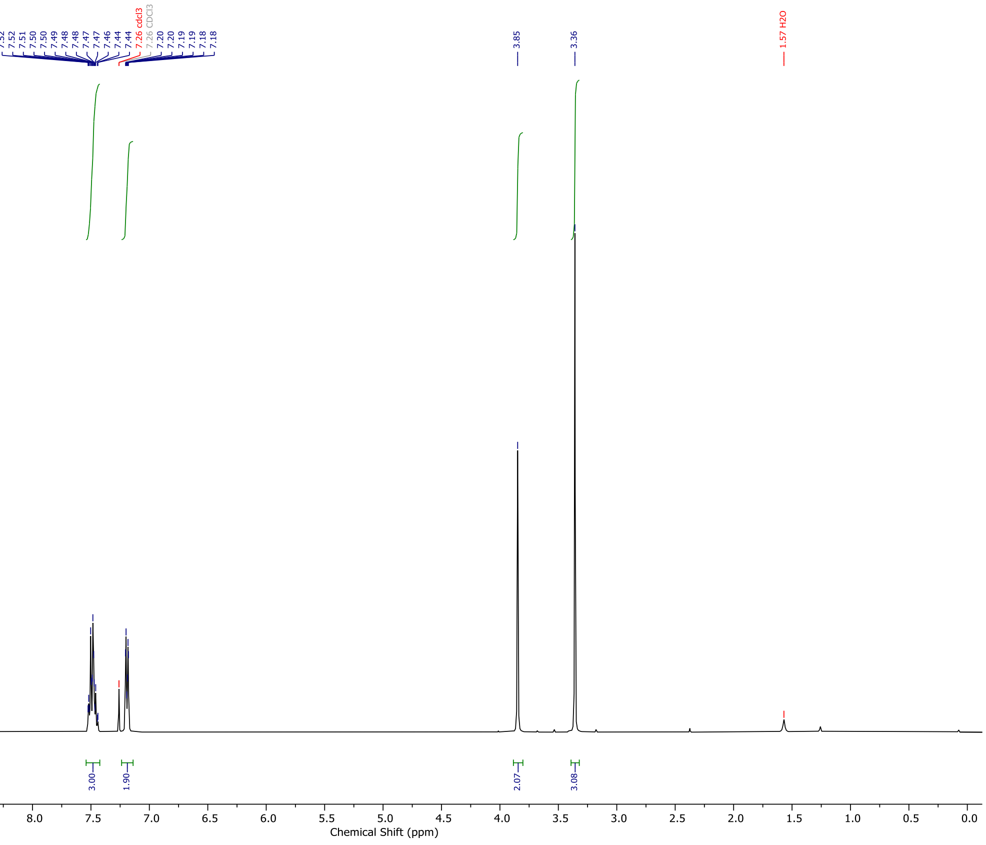
7.52 7.52 7.51 7.50 7.50 7.49 7.48 7.48 7.47 7.47 7.46 7.44 7.44
7.26 cdcl3
7.26 CDCl3
7.20 7.20 7.20 7.19 7.19 7.18 7.18
3.85
3.36
1.57 H2O
3.00
1.90
2.07
3.08
10.0 9.5 9.0 8.5 8.0 7.5 7.0 6.5 6.0 5.5 5.0 4.5 4.0 3.5 3.0 2.5 2.0 1.5 1.0 0.5 0.0
Chemical Shift (ppm)

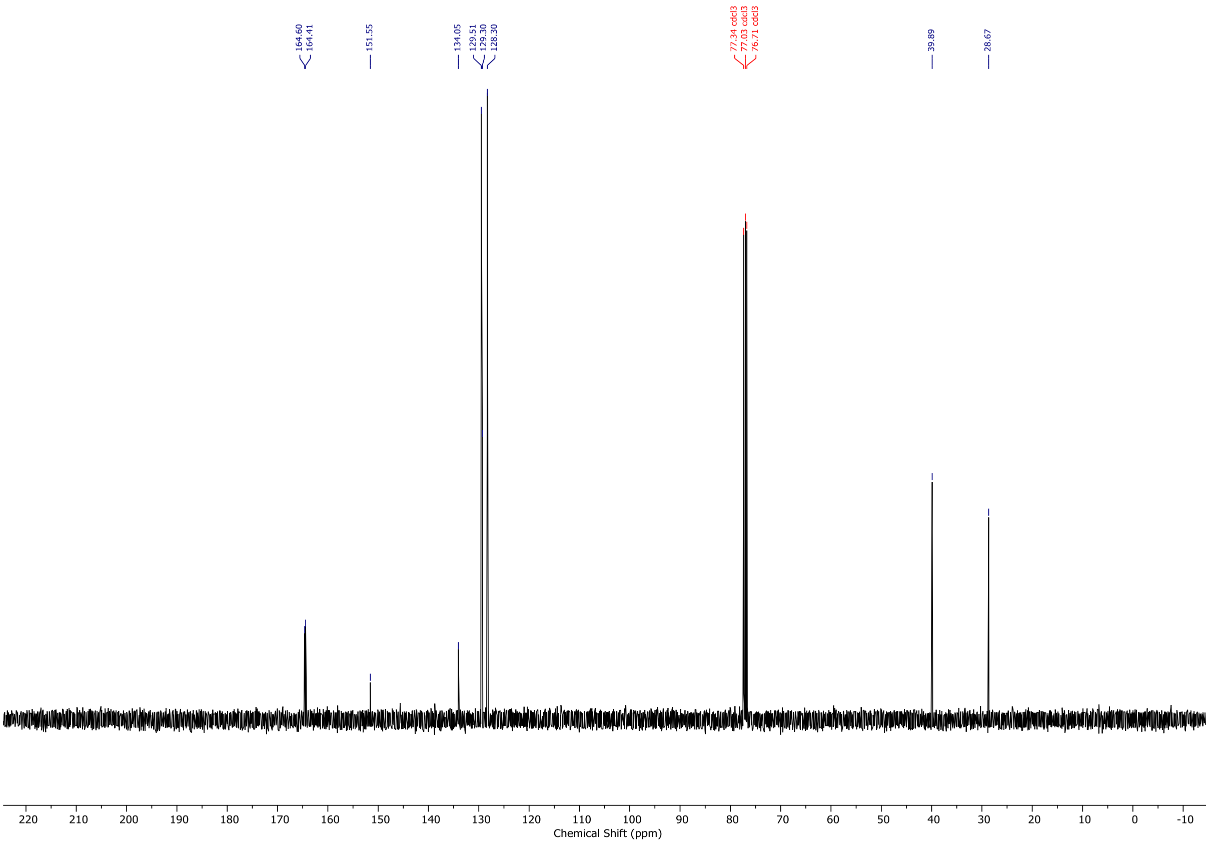
164.60 164.41
151.55
134.05 129.51 129.30 128.30
77.34 cdcl3 77.03 cdcl3 76.71 cdcl3
39.89
28.67
220 210 200 190 180 170 160 150 140 130 120 110 100 90 80 70 60 50 40 30 20 10 0 -10
Chemical Shift (ppm)

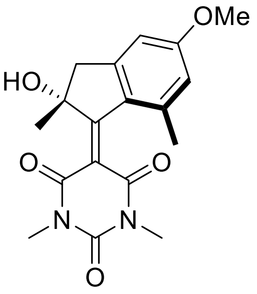

OMe
HO
O
O
N
N
O

1s

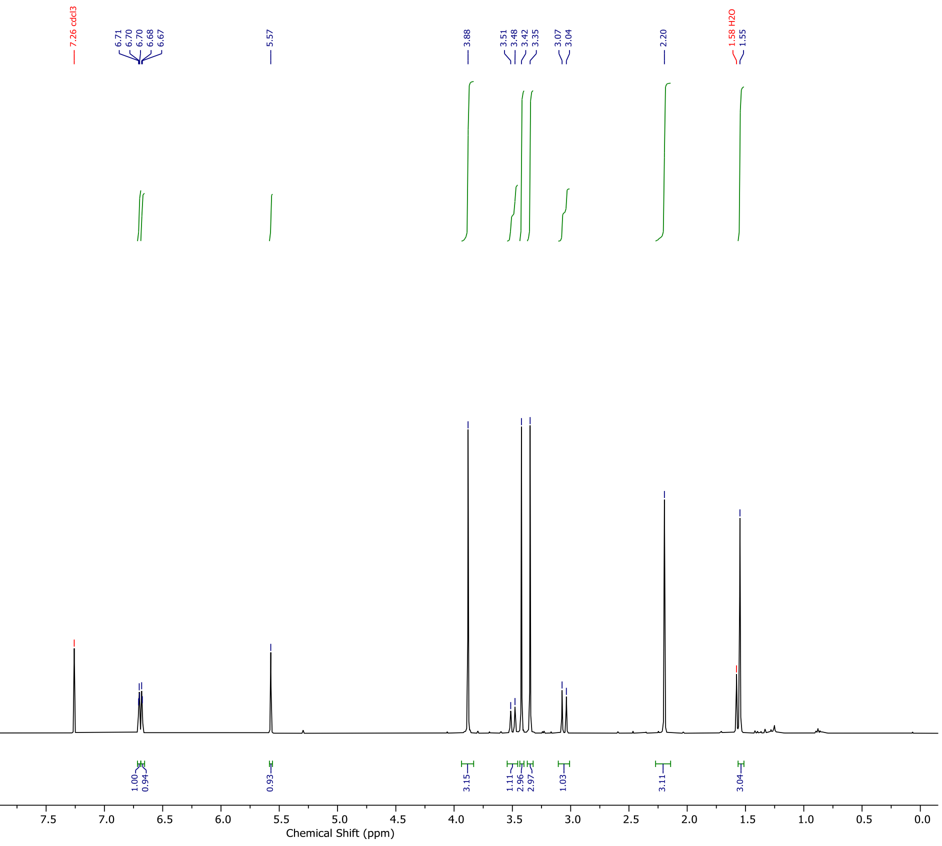

7.26 cdcl3
6.71
6.70
6.70
6.68
6.67
5.57
3.88
3.51
3.48
3.42
3.35
3.07
3.04
2.20
1.58 H2O
1.55
1.00
0.94
0.93
3.15
1.11
2.96
2.97
1.03
3.11
3.04
10.0 9.5 9.0 8.5 8.0 7.5 7.0 6.5 6.0 5.5 5.0 4.5 4.0 3.5 3.0 2.5 2.0 1.5 1.0 0.5 0.0
Chemical Shift (ppm)

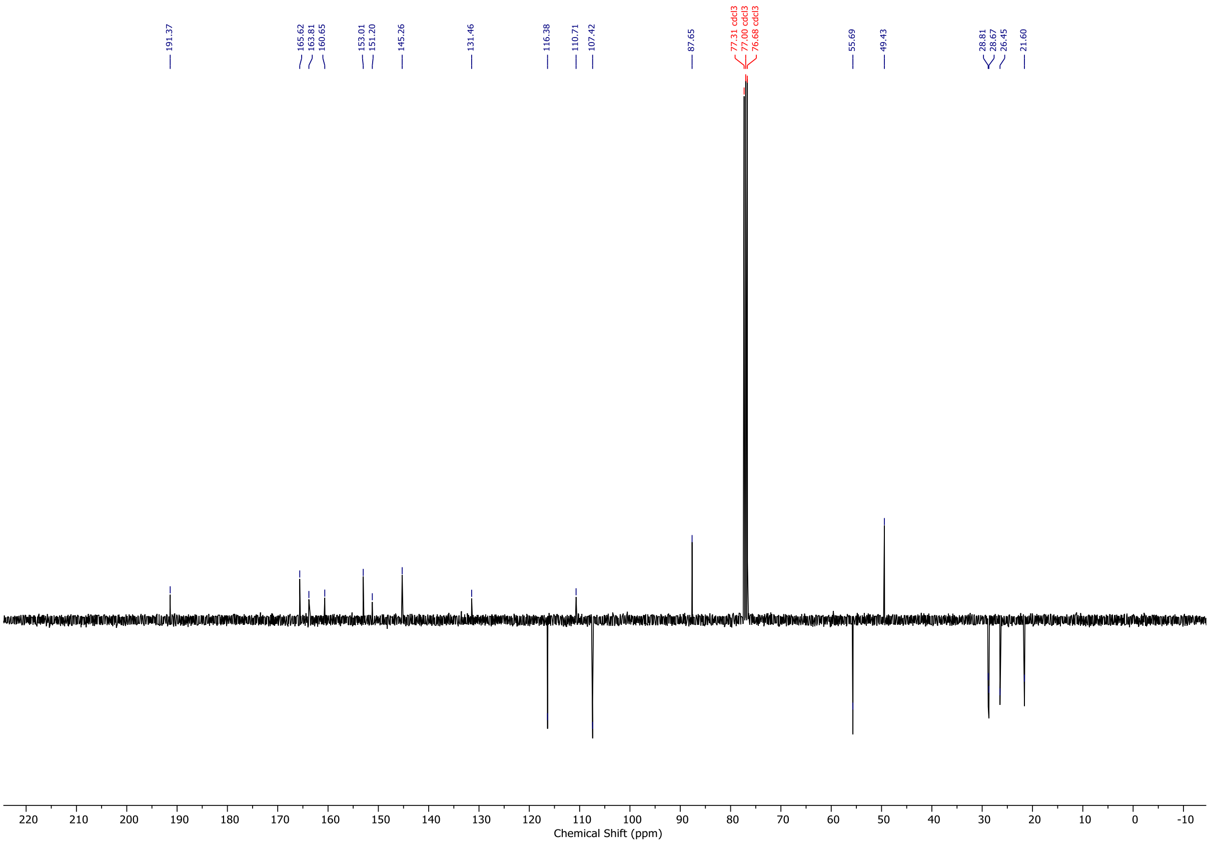

191.37
165.62
163.81
160.65
153.01
151.20
145.26
131.46
116.38
110.71
107.42
87.65
77.31 cdcl3
77.00 cdcl3
76.68 cdcl3
55.69
49.43
28.81
28.67
26.45
21.60
220 210 200 190 180 170 160 150 140 130 120 110 100 90 80 70 60 50 40 30 20 10 0 -10
Chemical Shift (ppm)

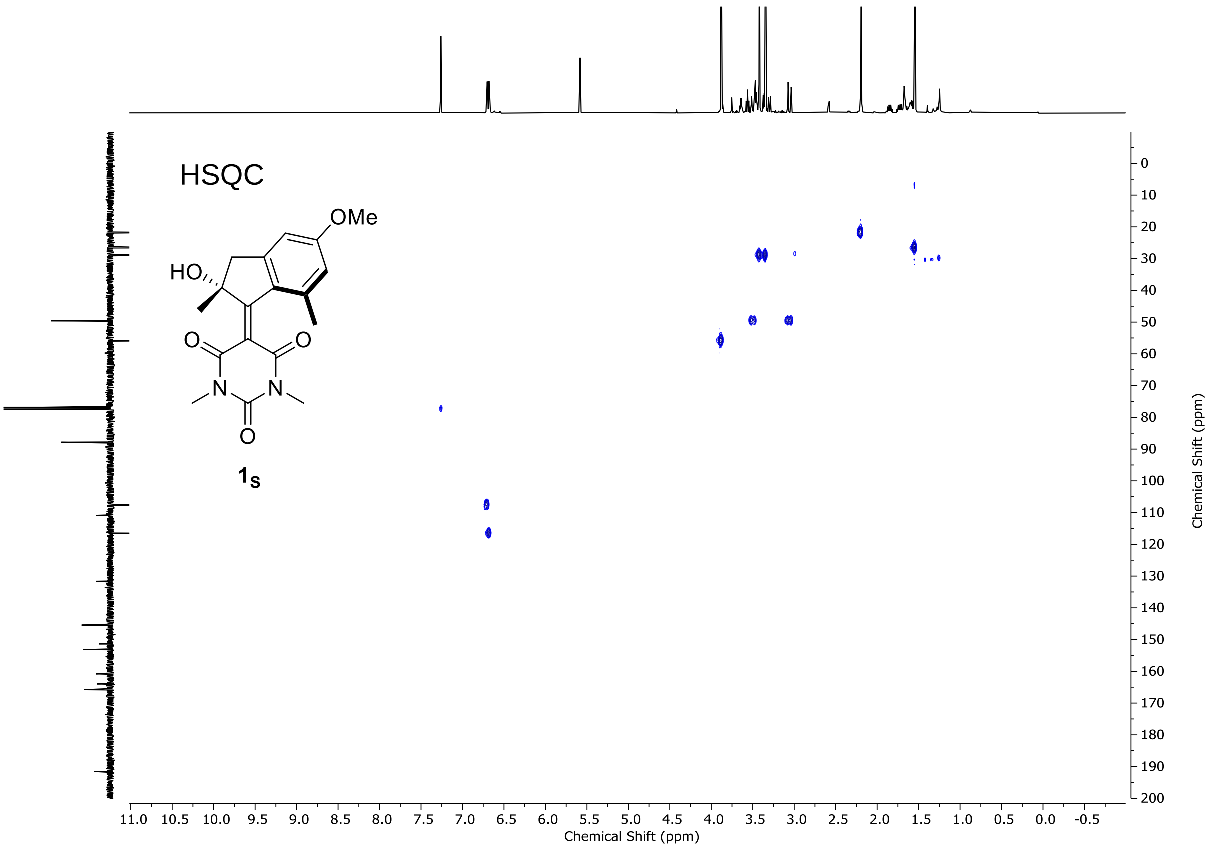
HSQC
OMe
HO
O
N
O
N
O
1s
Chemical Shift (ppm)
Chemical Shift (ppm)

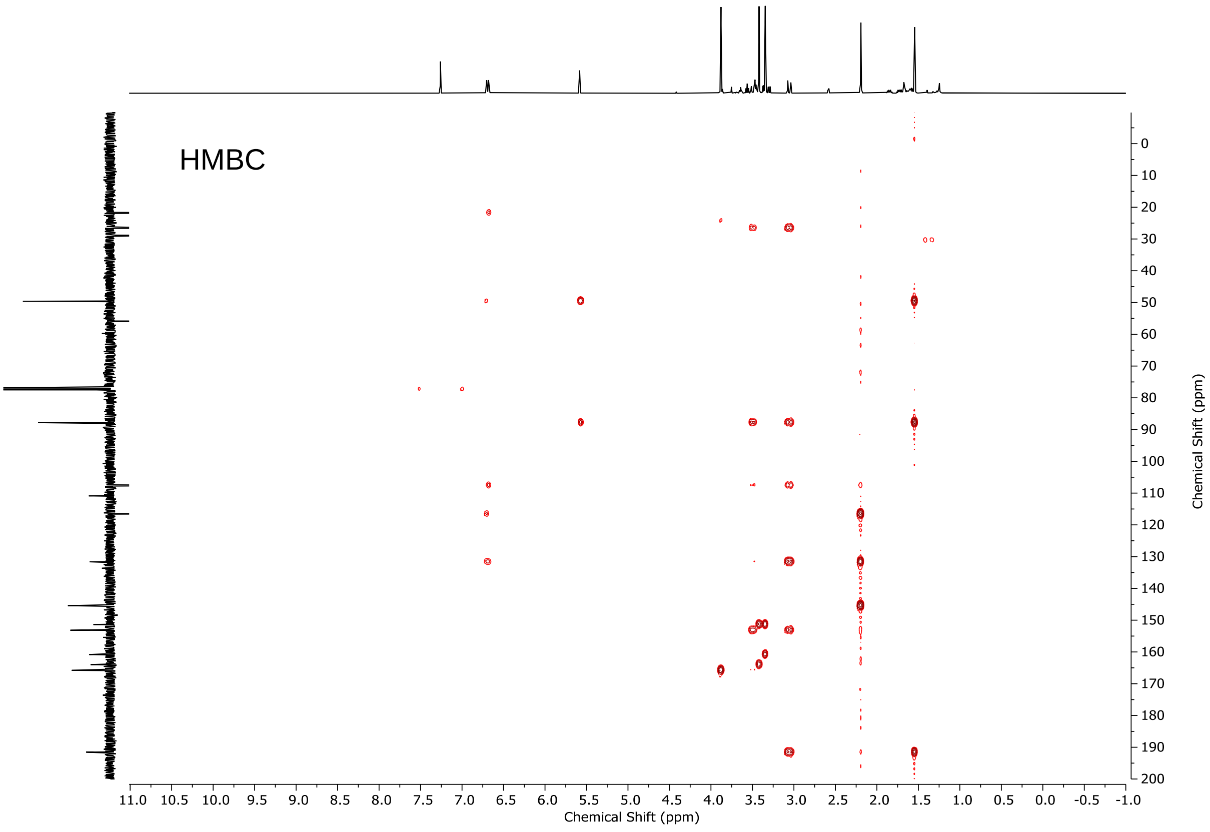
HMBC
Chemical Shift (ppm)
Chemical Shift (ppm)

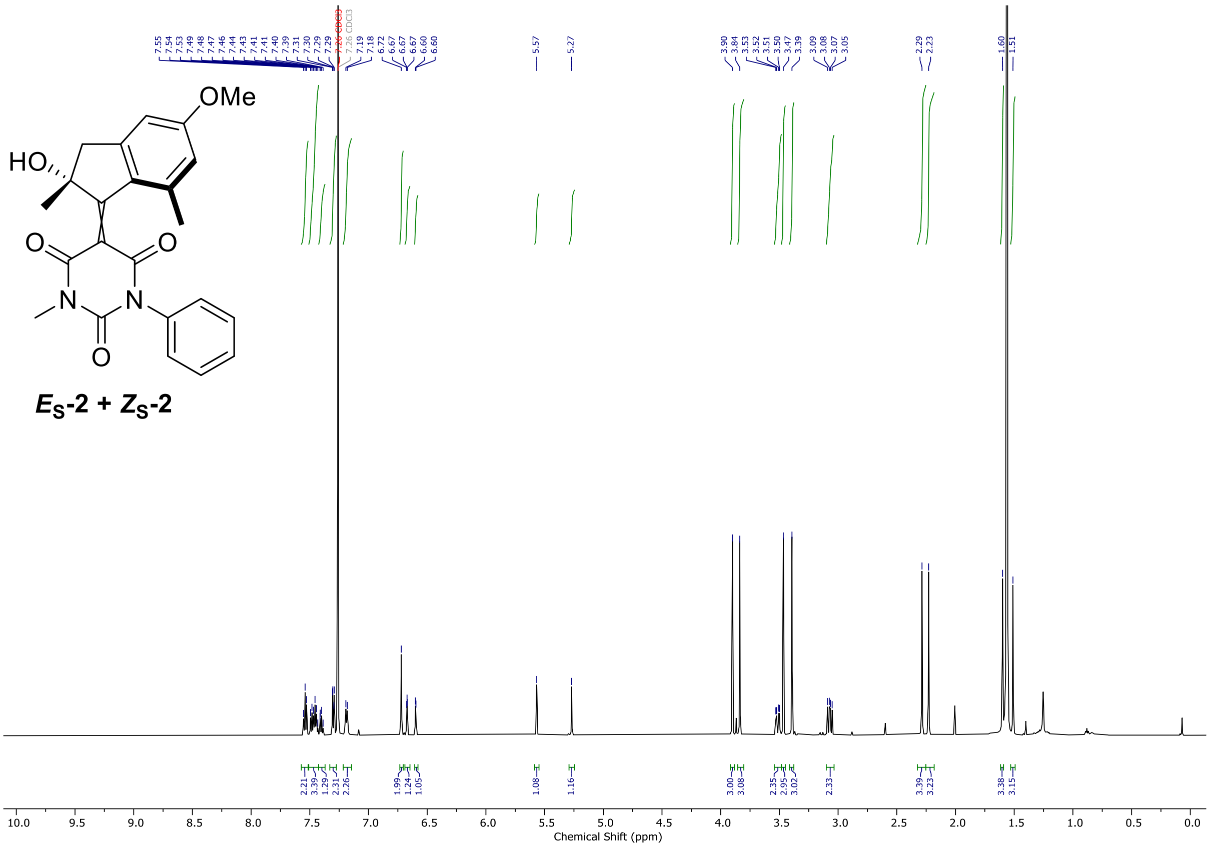

***E*$_S$-2 + *Z*$_S$-2**

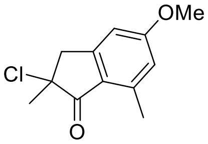
OMe
Cl
O

Cl-S1

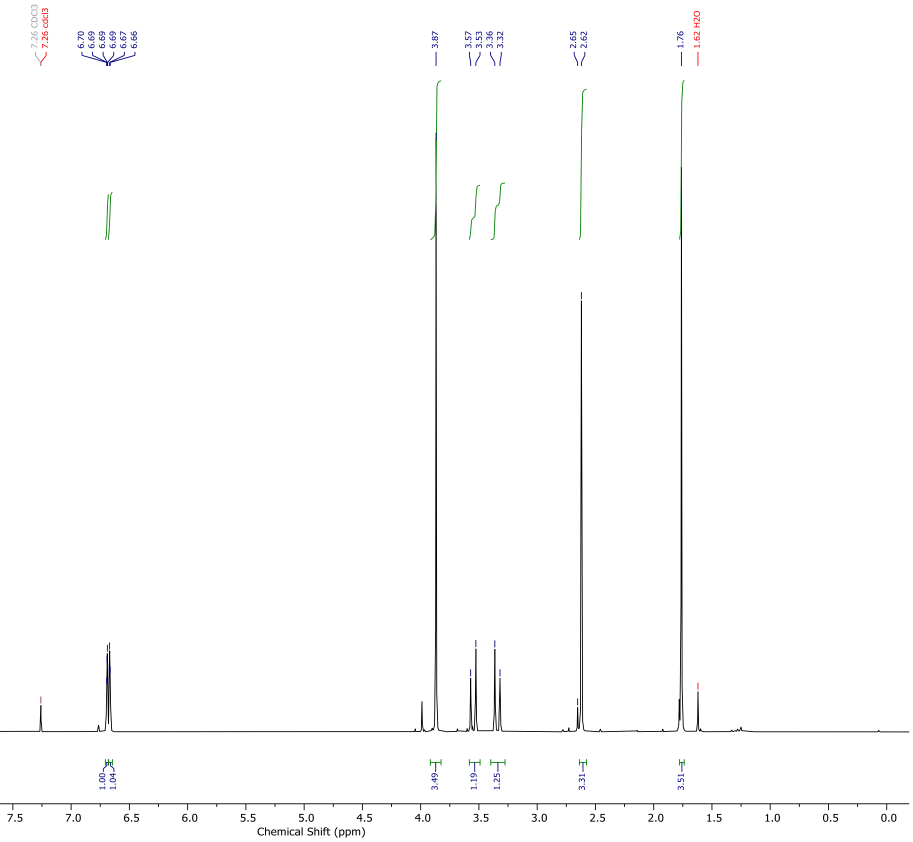
7.26 CDCl3
7.26 cdcl3
6.70
6.69
6.69
6.69
6.67
6.66
3.87
3.57
3.53
3.36
3.32
2.65
2.62
1.76
1.62 H2O
1.00
1.04
3.49
1.19
1.25
3.31
3.51
10.0 9.5 9.0 8.5 8.0 7.5 7.0 6.5 6.0 5.5 5.0 4.5 4.0 3.5 3.0 2.5 2.0 1.5 1.0 0.5 0.0
Chemical Shift (ppm)

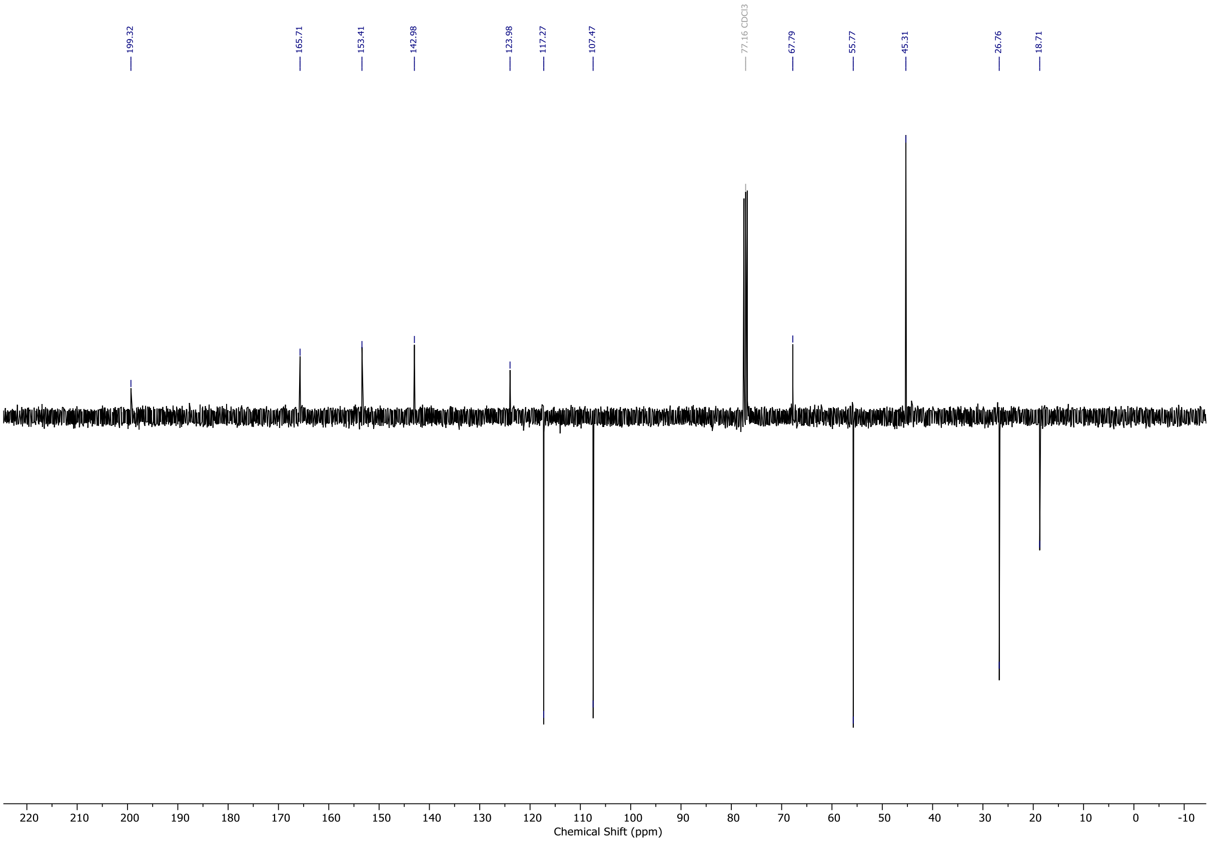
199.32
165.71
153.41
142.98
123.98
117.27
107.47
77.16 CDCl3
67.79
55.77
45.31
26.76
18.71
220 210 200 190 180 170 160 150 140 130 120 110 100 90 80 70 60 50 40 30 20 10 0 -10
Chemical Shift (ppm)

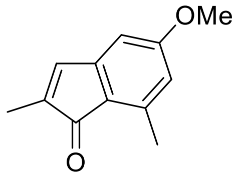
OMe
O

E1

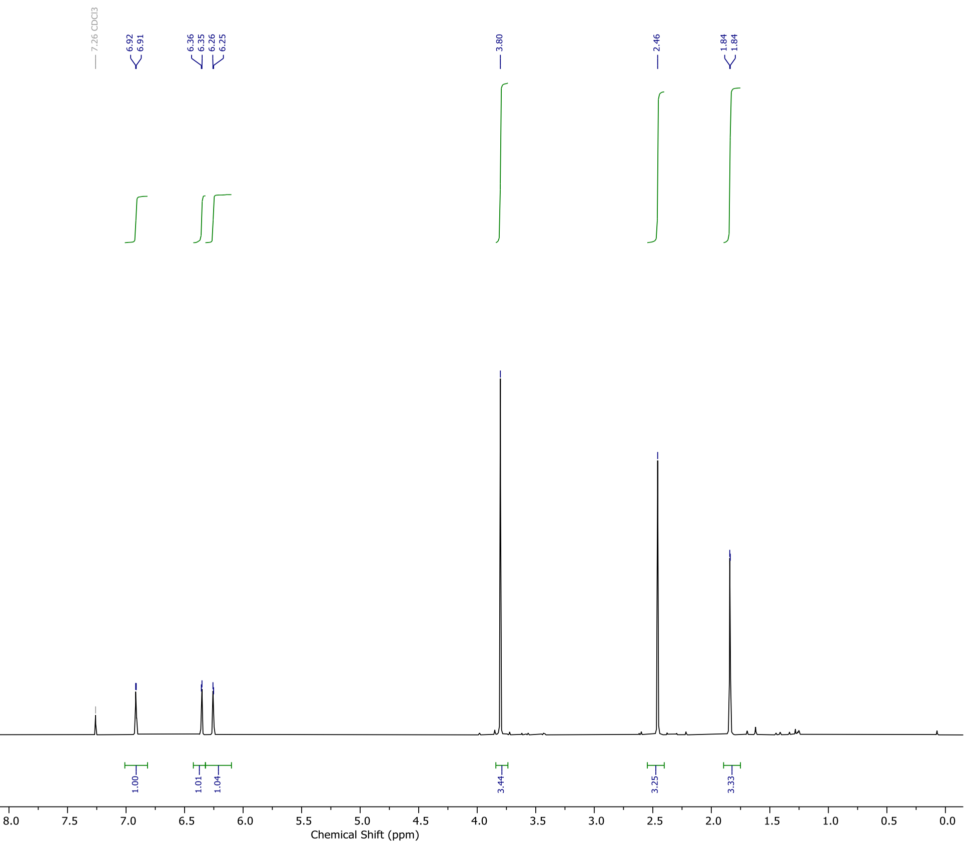
7.26 CDCl3
6.92
6.91
6.36
6.35
6.26
6.25
3.80
2.46
1.84
1.84
1.00
1.01
1.04
3.44
3.25
3.33
10.0 9.5 9.0 8.5 8.0 7.5 7.0 6.5 6.0 5.5 5.0 4.5 4.0 3.5 3.0 2.5 2.0 1.5 1.0 0.5 0.0
Chemical Shift (ppm)

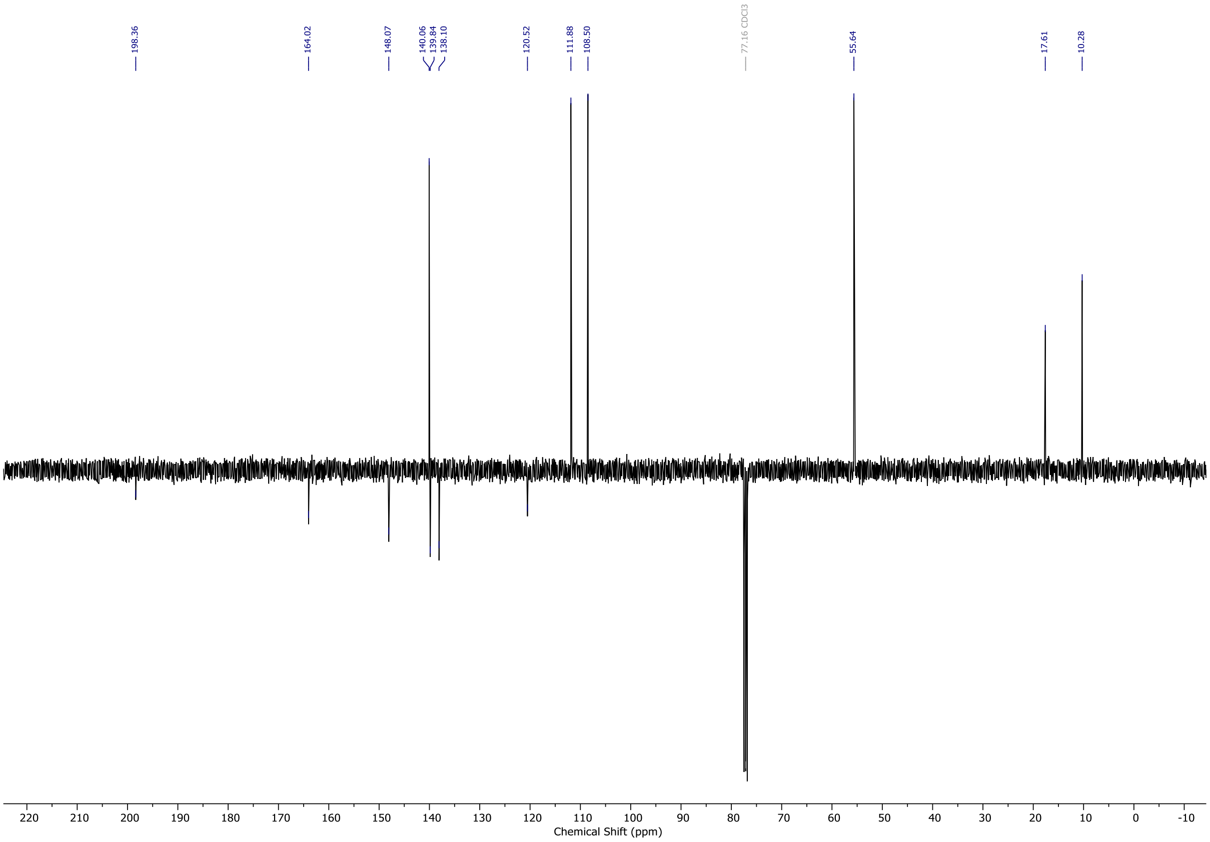
198.36
164.02
148.07
140.06
139.84
138.10
120.52
111.88
108.50
77.16 CDCl3
55.64
17.61
10.28
220 210 200 190 180 170 160 150 140 130 120 110 100 90 80 70 60 50 40 30 20 10 0 -10
Chemical Shift (ppm)
